# Supplementary figures and images for: Analysis of the SNARE Stx8 recycling reveals that the retromer-sorting motif has undergone evolutionary divergence
Source: PLoS Genet. 2021 Mar 31;17(3):e1009463. doi: 10.1371/journal.pgen.1009463 (PMC8041195; doi:10.1371/journal.pgen.1009463)

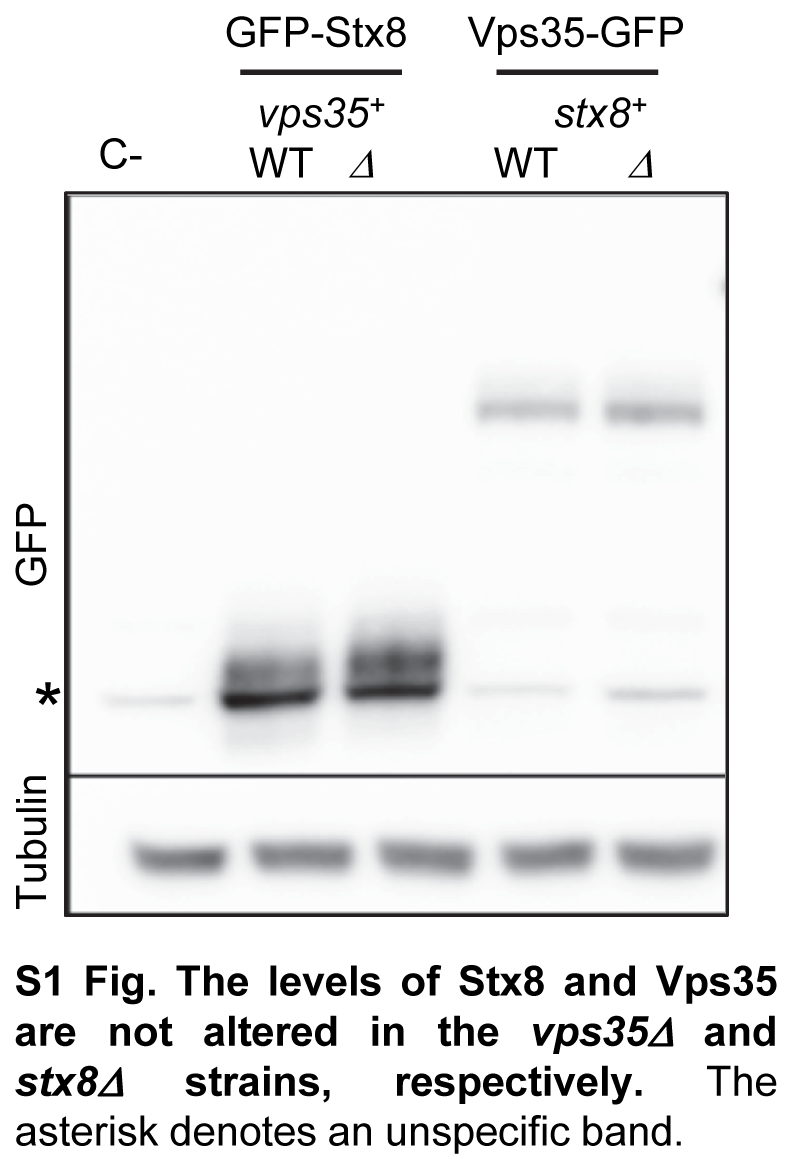

Supplement: S1 Fig — The asterisk denotes an unspecific band. (TIF) [file pgen.1009463.s001.tif]

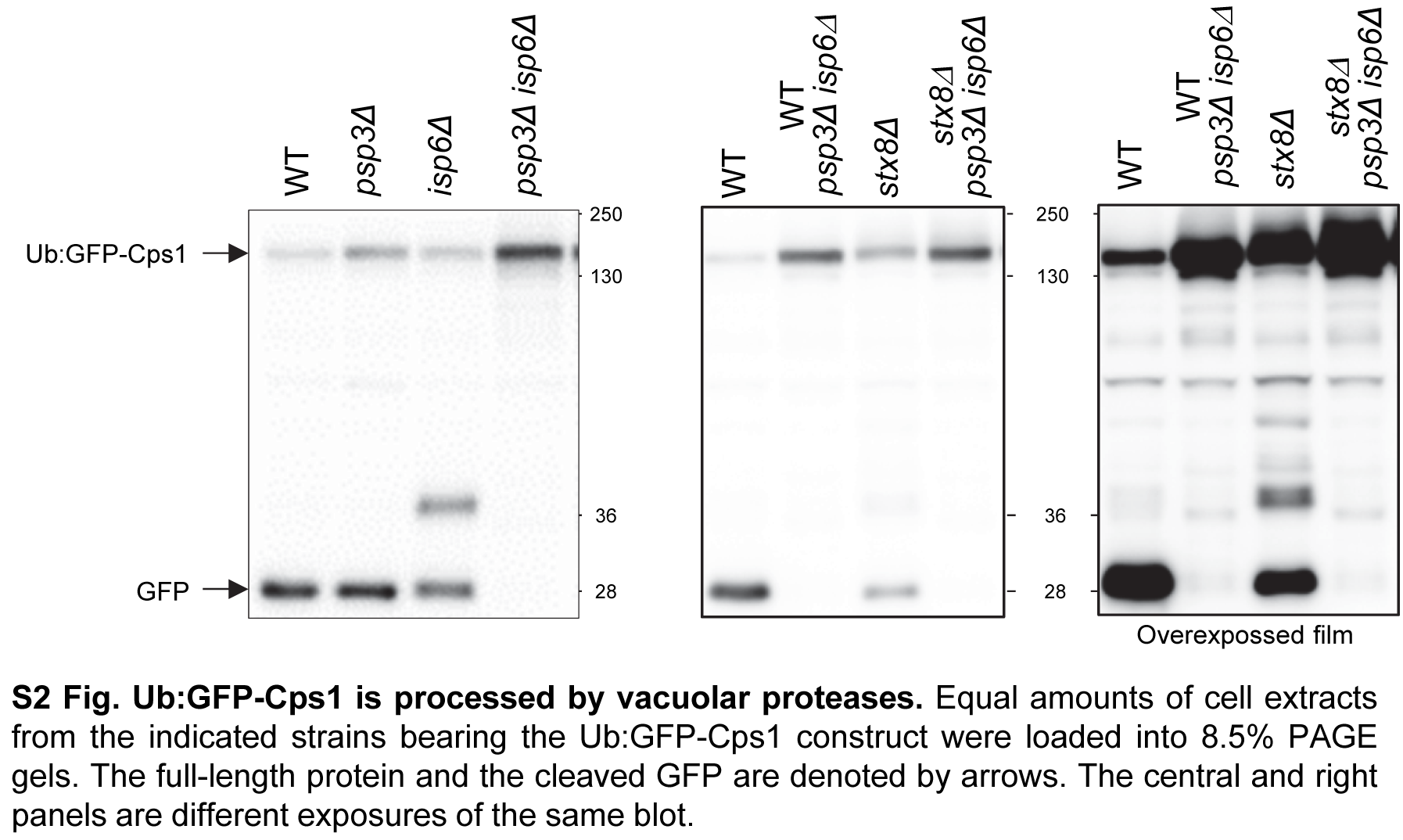

Supplement: S2 Fig — Equal amounts of cell extracts from the indicated strains bearing the Ub:GFP-Cps1 construct were loaded into 8.5% PAGE gels. The full-length protein and the cleaved GFP are denoted by arrows. The central and right panels are different exposures of the same blot. (TIF) [file pgen.1009463.s002.tif]

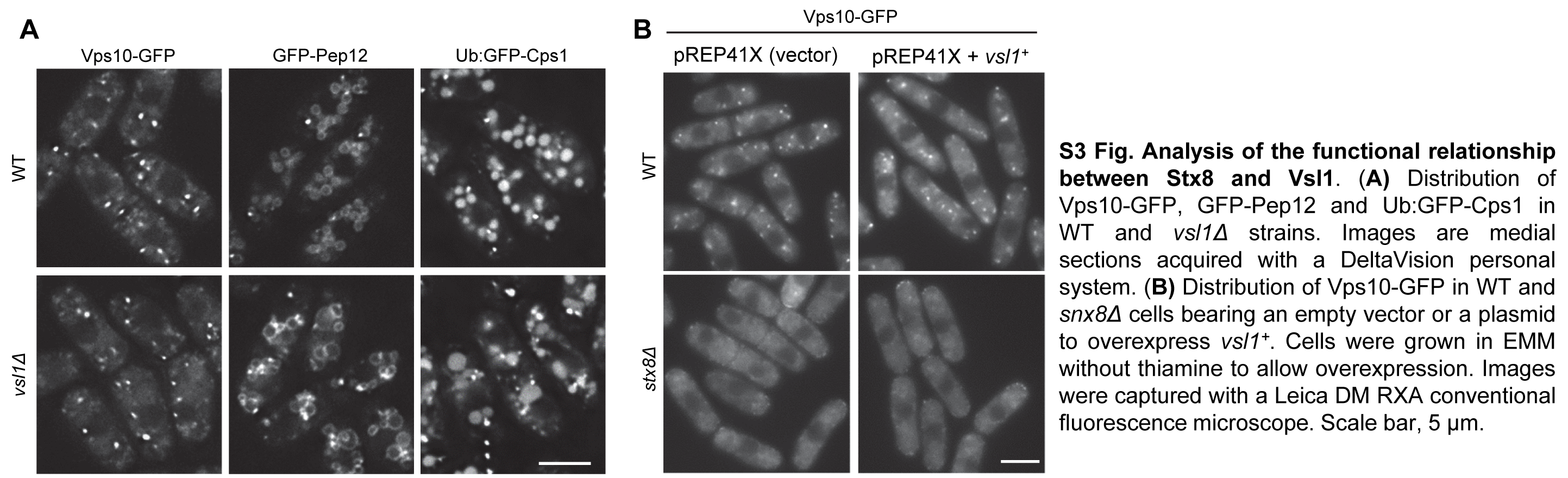

Supplement: S3 Fig — (A) Distribution of Vps10-GFP, GFP-Pep12 and Ub:GFP-Cps1 in WT and vsl1Δ strains. Images are medial sections acquired with a DeltaVision personal system. (B) Distribution of Vps10-GFP in WT and snx8Δ cells bearing an empty vector or a plasmid to overexpress vsl1+. Cells were grown in EMM without thiamine to allow overexpression. Images were captured with a Leica DM RXA conventional fluorescence microscope. Scale bar, 5 μm. (TIF) [file pgen.1009463.s003.tif]

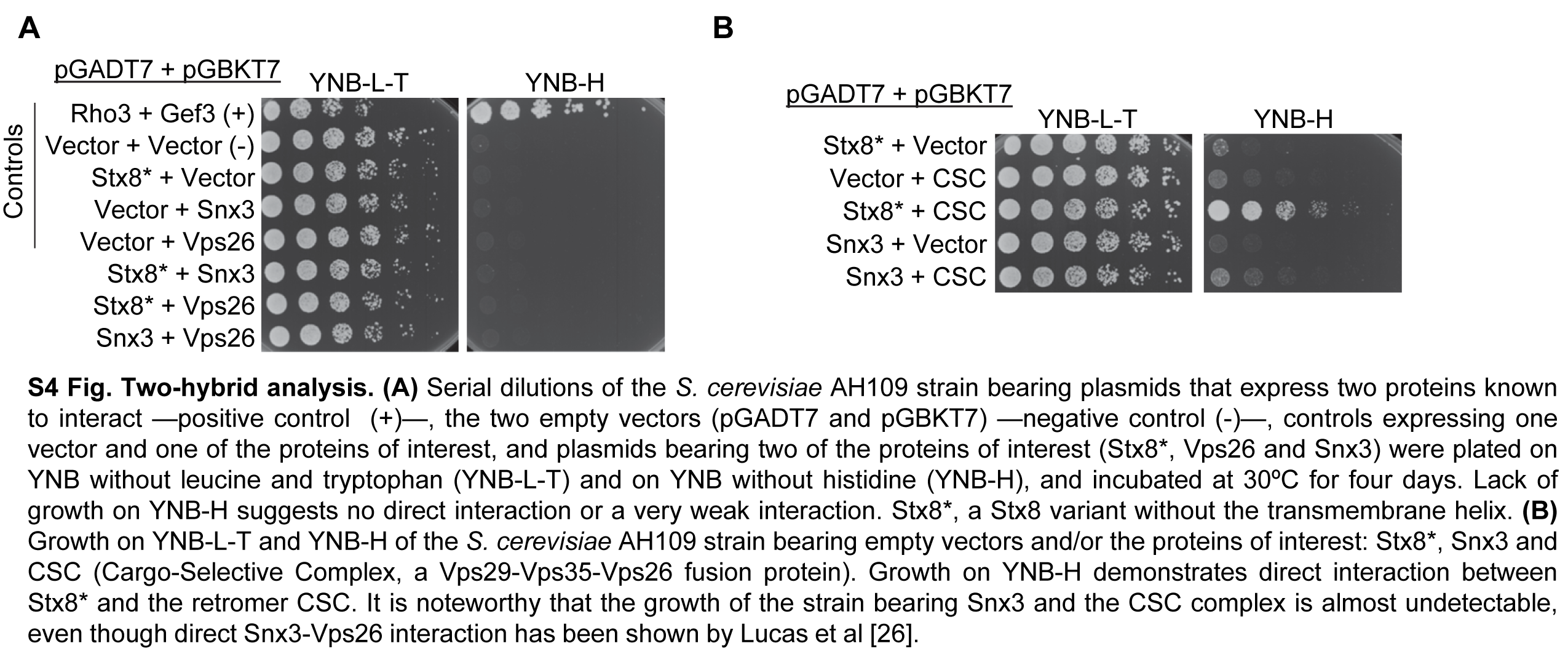

Supplement: S4 Fig — (A) Serial dilutions of the S. cerevisiae AH109 strain bearing plasmids that express two proteins known to interact—positive control (+)—, the two empty vectors (pGADT7 and pGBKT7)—negative control (-)—, controls expressing one vector and one of the proteins of interest, and plasmids bearing two of the proteins of interest (Stx8*, Vps26 and Snx3) were plated on YNB without leucine and tryptophan (YNB-L-T) and on YNB without histidine (YNB-H), and incubated at 30°C for four days. Lack of growth on YNB-H suggests no direct interaction or a very weak interaction. Stx8*, a Stx8 variant without the transmembrane helix. (B) Growth on YNB-L-T and YNB-H of the S. cerevisiae AH109 strain bearing empty vectors and/or the proteins of interest: Stx8*, Snx3 and CSC (Cargo-Selective Complex, a Vps29-Vps35-Vps26 fusion protein). Growth on YNB-H demonstrates direct interaction between Stx8* and the retromer CSC. It is noteworthy that the growth of the strain bearing Snx3 and the CSC complex is almost undetectable, even though direct Snx3-Vps26 interaction has been shown by Lucas et al [26]. (TIF) [file pgen.1009463.s004.tif]

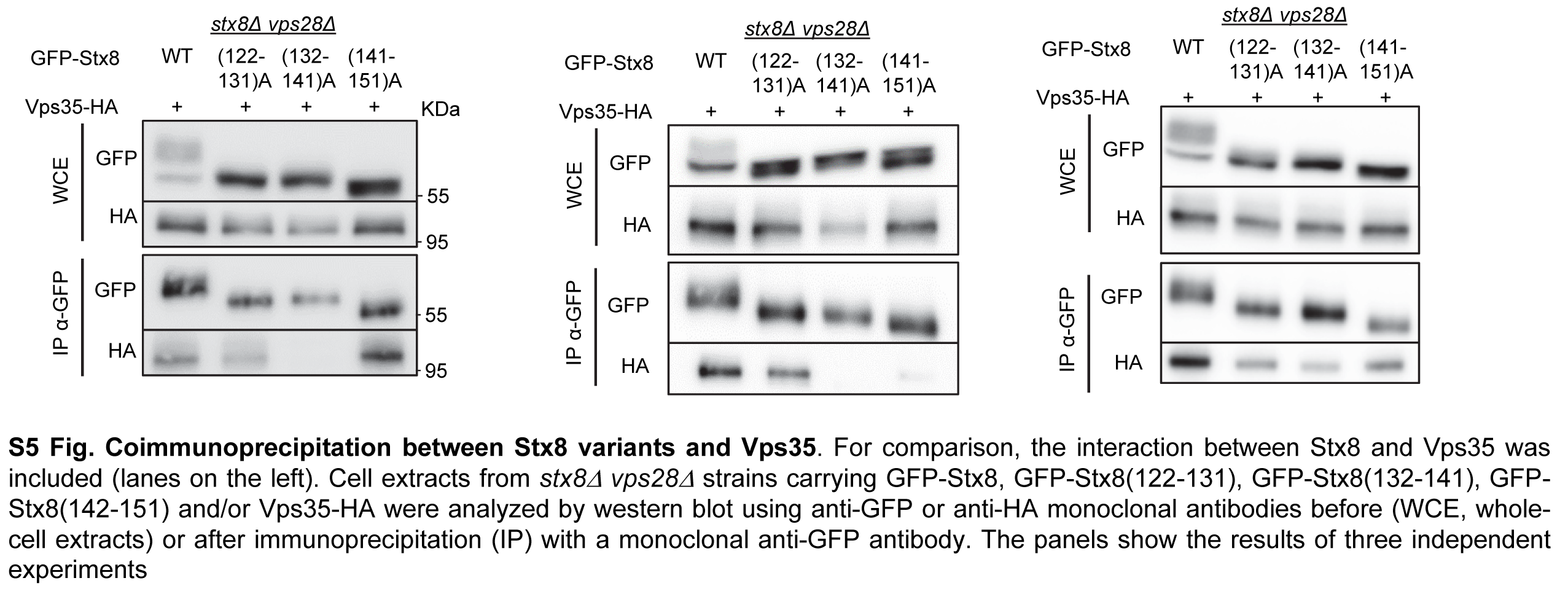

Supplement: S5 Fig — For comparison, the interaction between Stx8 and Vps35 was included (lanes on the left). Cell extracts from stx8Δ vps28Δ strains carrying GFP-Stx8, GFP-Stx8(122–131), GFP-Stx8(132–141), GFP-Stx8(142–151) and/or Vps35-HA were analyzed by western blot using anti-GFP or anti-HA monoclonal antibodies before (WCE, whole-cell extracts) or after immunoprecipitation (IP) with a monoclonal anti-GFP antibody. The panels show the results of three independent experiments. (TIF) [file pgen.1009463.s005.tif]

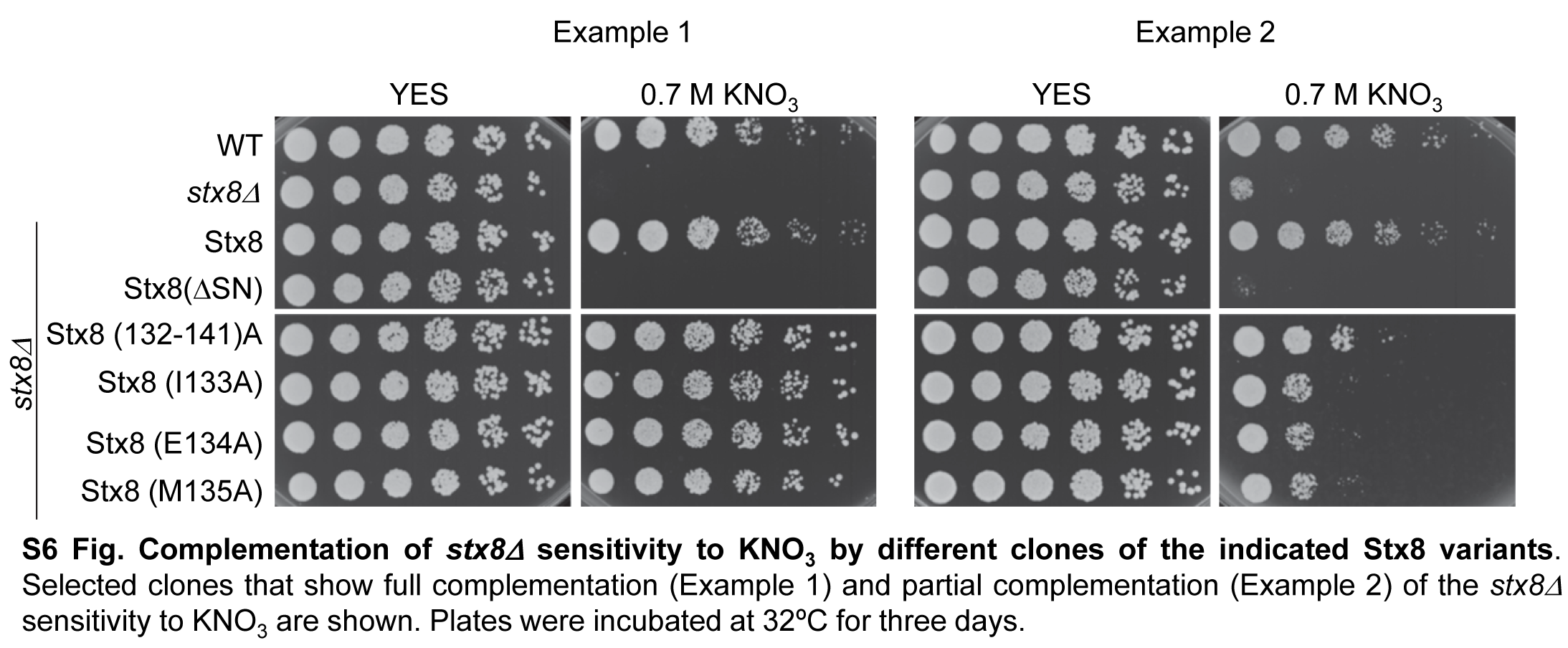

Supplement: S6 Fig — Selected clones that show full complementation (Example 1) and partial complementation (Example 2) of the stx8Δ sensitivity to KNO3 are shown. Plates were incubated at 32°C for three days. (TIF) [file pgen.1009463.s006.tif]

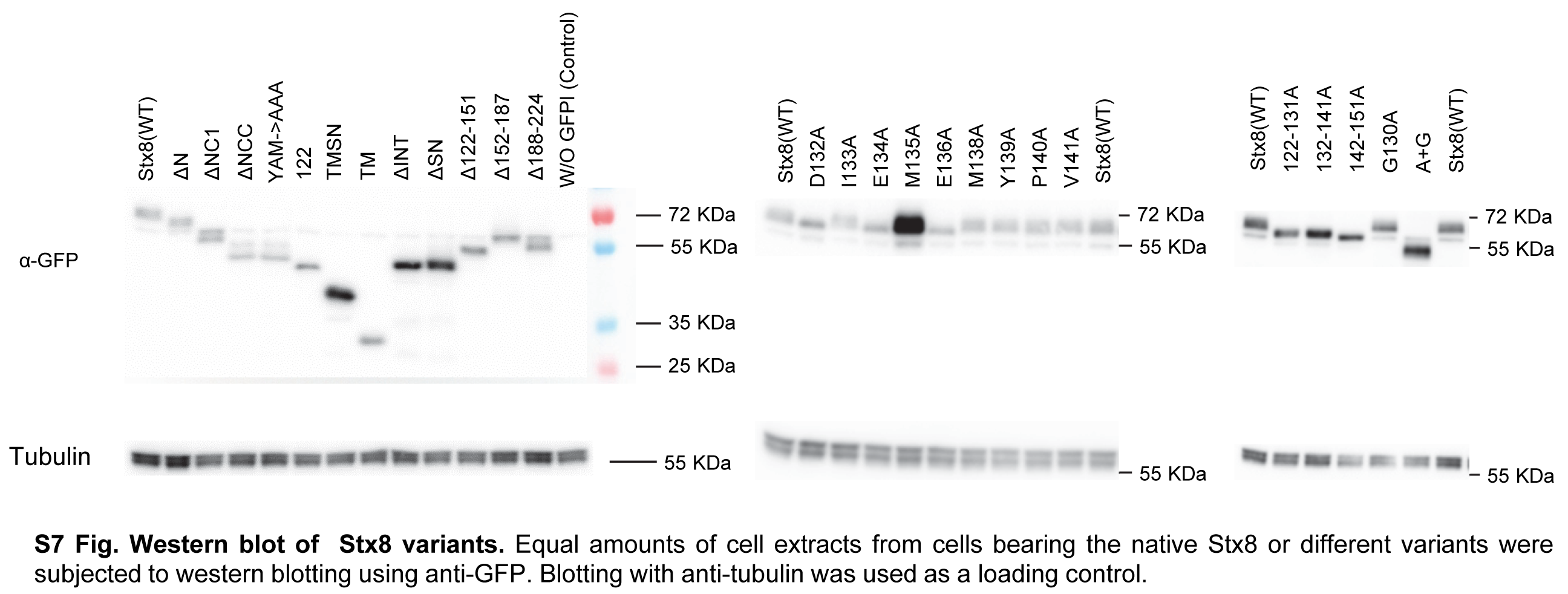

Supplement: S7 Fig — Equal amounts of cell extracts from cells bearing the native Stx8 or different variants were subjected to western blotting using anti-GFP. Blotting with anti-tubulin was used as a loading control. (TIF) [file pgen.1009463.s007.tif]

**S2 File. Coiled-coil predictions (NCOILS version 1.0. MTIDK)**

**Stx8 Stx8(ΔSN)**


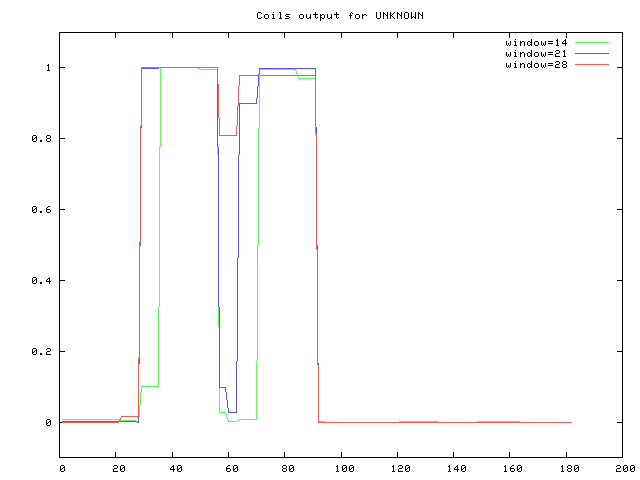

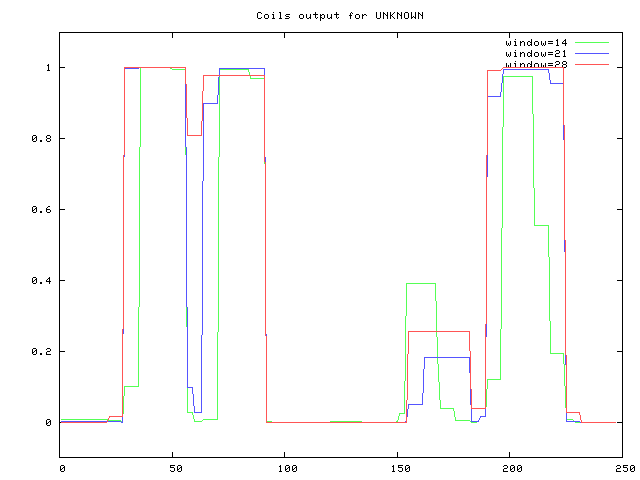


**Stx8(Δ152-187) Stx8(Δ188-224)**


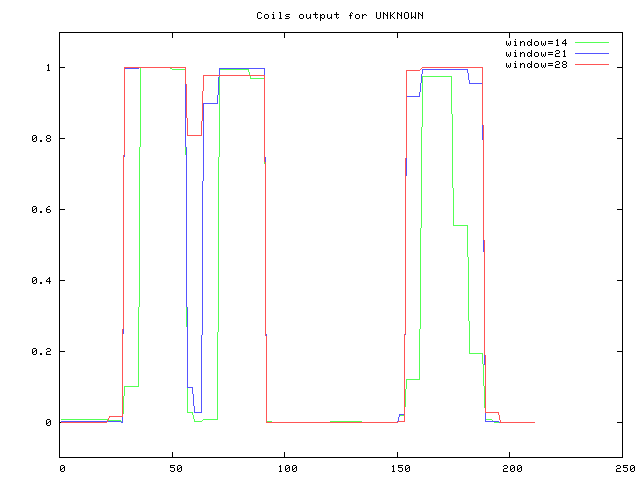


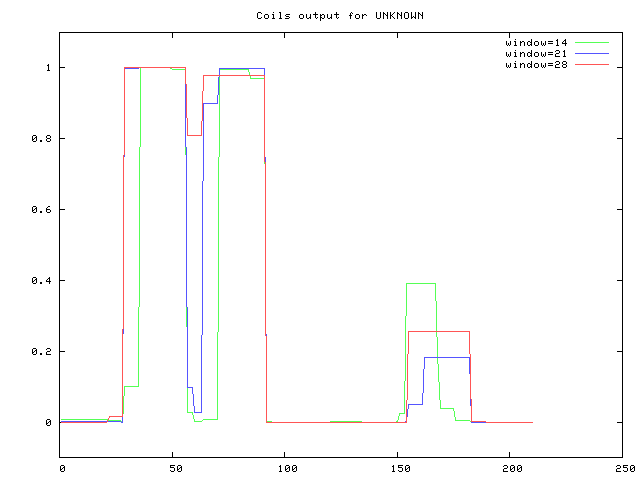

Supplement: S2 File — NCOILS prediction of coiled-coils in Stx8 variants (DOCX) [file pgen.1009463.s010.docx]
