# Supplementary material for: Analysis of the SNARE Stx8 recycling reveals that the retromer-sorting motif has undergone evolutionary divergence
Source: PLoS Genet. 2021 Mar 31;17(3):e1009463. doi: 10.1371/journal.pgen.1009463 (PMC8041195; doi:10.1371/journal.pgen.1009463)
Supplement: S1 Table — Genotype and source of the strains used in this work. (DOCX) [file pgen.1009463.s008.docx]

**S1 Table. List of yeast strains used in this work**

| **STRAIN** | **GENOTYPE** | **SOURCE** |
| --- | --- | --- |
| HVP30 | *leu1-32 his3-∆1 ura4-∆18 ade6 h-* | Lab stock |
| HVP117 | *leu1-32 his3-∆1 ura4-∆18 ade6 h+* | Lab stock |
| HVP2034 | *972 h-* (WT) | Lab stock |
| HVP2273 | *GFP-syb1 leu1-32 h-* | Y Toyoshima |
| HVP3664 | *vps27::KAN leu1-32 ura4-Δ18 ade6 h+* | P Perez |
| HVP3902 | *GFP-Sec72::ura4 leu1-32 his3-∆1 ura4 h-* | Lab stock |
| HVP3945 | *pku70::his3+ leu1-32 his3-∆1 ura4-∆18 ade6 h+* | I Hagan/ YGRC |
| HVP3953 | *hph.171 h-* | I Hagan/ YGRC |
| HVP4068 | *hph.171 leu1-32 his3-∆1 ura4-∆18 h+* | This work |
| HVP4694 | *vps35::NAT leu1-32 his3-∆1 ura4-∆18 ade6 h+* | Lab stock |
| HVP4695 | *vps35::NAT leu1-32 his3-∆1 ura4-∆18 ade6 h-* | Lab stock |
| HVP4708 | *vps10-GFP:KAN leu1-32 his3-∆1 ura4-∆18 ade6 h-* | Lab stock |
| HVP4709 | *vps10-GFP:KAN leu1-32 his3-∆1 ura4-∆18 ade6 h+* | Lab stock |
| HVP4715 | *vps27-RFP:KAN leu1-32 his3-∆1 ura4-∆18 ade6 h-* | Lab stock |
| HVP4821 | *vps10-GFP:KAN vps35::NAT leu1-32 his3-Δ1 ura4-∆18 ade6 h-* | Lab stock |
| HVP4857 | *Pnda2:GFP-vti1:Tnda2:NAT leu1-32 his3-∆1 ura4-∆18 h+* | This work |
| HVP4876 | *Pnda2:GFP-Pep12:Tnda2:NAT leu1-32 his3-∆1 ura4-∆18 h+* | Lab stock |
| HVP4877 | *Pnda2:GFP-stx8:Tnda2:NAT leu1-32 his3-∆1 ura4-∆18 h+* | This work |
| HVP4878 | *Pnda2:GFP-ykt6:Tnda2:NAT leu1-32 his3-∆1 ura4-∆18 h+* | This work |
| HVP4941 | *stx8::HPH leu1-32 his3-∆1 ura4-∆18 ade6 h-* | This work |
| HVP4942 | *stx8::HPH leu1-32 his3-∆1 ura4-∆18 ade6 h+* | This work |
| HVP5002 | *vps10-GFP:KAN stx8::HPH leu1-32 his3-∆1 ura4-∆18 ade6 h-* | This work |
| HVP5003 | *vps10-GFP:KAN stx8::HPH leu1-32 his3-∆1 ura4-∆18 ade6 h+* | This work |
| HVP5021 | *Pnda2:GFP-pep12:Tnda2:NAT vsl1::KAN leu1-32 his3-∆1 ura4-∆18 h?* | This work |
| HVP5029 | *vps10-GFP:KAN stx8::HPH vps35::NAT leu1-32 his3-∆1 ura4-∆18 ade6 h-* | This work |
| HVP5033 | *vps27-RFP::KAN stx8::HPH leu1-32 his3-∆1 ura4-∆18 ade6 h+* | This work |
| HVP5038 | *vps35-GFP:NAT leu1-32 his3-∆1 ura4-∆18 ade6 h+* | Lab stock |
| HVP5039 | *vps35-GFP:NAT leu1-32 his3-∆1 ura4-∆18 ade6 h-* | Lab stock |
| HVP5053 | *Pnda2:GFP-stx8:Tnda2:NAT vps35::NAT leu1-32 his3-∆1 ura4-∆18 h?* | This work |
| HVP5061 | *Pnda2:GFP-pep12:Tnda2:NAT stx8::HPH leu1-32 his3-∆1 ura4-∆18 h-* | This work |
| HVP5062 | *Pnda2:GFP-pep12:Tnda2:NAT stx8::HPH leu1-32 his3-∆1 ura4-∆18 h+* | This work |
| HVP5074 | *Pnda2:GFP-ykt6Tnda2:NAT vps35::NAT leu1-32 his3-∆1 ura4-∆18 h+* | This work |
| HVP5076 | *Pnda2:GFP-vti1:Tnda2:NAT vps35::NAT leu1-32 his3-∆1 ura4-∆18 h+* | This work |
| HVP5077 | *Pnda2:GFP-pep12:Tnda2:NAT vps35::NAT leu1-32 his3-∆1 ura4-∆18 h-* | This work |
| HVP5080 | *Pnda2:GFP-pep12:Tnda2:NAT apm3::KAN leu1-32 his3-∆1 ura4-∆18 h+* | This work |
| HVP5084 | *GFP-syb1::leu1 leu1-32 vps35::NAT h-* | This work |
| HVP5094 | *Pnda2:GFP-pep12:Tnda2:NAT apm3::KAN stx8::HPH h+* | This work |
| HVP5096 | *cpy1-mCherry:KAN leu1-32 his3-∆1 h-* | L L Du |
| HVP5099 | *vps35-GFP::NAT stx8::HPH leu1-32 his3-∆1 ura4-∆18 ade6 h?* | This work |
| HVP5104 | *cpy1-mCherry:KAN stx8::HPH leu1-32 his3-∆1 h-* | This work |
| HVP5128 | *Pnda2:ub:GFP-cps1:Tnmt1:NAT leu1-32 his3-∆1 ura4-∆18 ade6 h-* | Lab stock |
| HVP5129 | *Pnda2:ub:GFP-Cps1:Tnmt1:NAT stx8::HPH leu1-32 his3-∆1 ura4-∆18 h-* | This work |
| HVP5130 | *Pnda2:ub:GFP-Cps1:Tnmt1:NAT stx8::HPH leu1-32 his3-∆1 ura4-∆18 h+* | This work |
| HVP5150 | *Pnda2:GFP-stx8:Tnda2:NAT cfr1-RFP:ura4 cfr1::his3 leu1-32 his3-∆1 ura4-∆18 h?* | This work |
| HVP5162 | *Pnda2:GFP-stx8:Tnda2:NAT vps27-RFP:KAN leu1-32 his3-∆1 ura4-∆18 h?* | This work |
| HVP5168 | *Pnda2:ub:GFP-cps1:Tnmt1:NAT vps27::KAN leu1-32 his3-∆1 ura4-∆18 ade6 h-* | Lab stock |
| HVP5234 | *Pnda2:GFP-stx8TM:Tnda2:NAT leu1-32 his3-∆1 ura4-∆18 h+* | This work |
| HVP5235 | *Pnda2:GFP-stx8SNTM:Tnda2:NAT leu1-32 his3-∆1 ura4-∆18 h+* | This work |
| HVP5236 | *Pnda2:GFP-stx8ΔNCC:Tnda2:NAT leu1-32 his3-∆1 ura4-∆18 h+* | This work |
| HVP5237 | *Pnda2:GFP-stx8ΔNC1:Tnda2:NAT leu1-32 his3-∆1 ura4-∆18 h+* | This work |
| HVP5238 | *Pnda2:GFP-stx8ΔN:Tnda2:NAT leu1-32 his3-∆1 ura4-∆18 h+* | This work |
| HVP5246 | *Pnda2:GFP-stx8 stx8ΔNCC:Tnda2:NAT vps35::NAT leu1-32 his3-∆1 ura4-∆18 h+* | This work |
| HVP5247 | *GFP-sec72::ura4 stx8::HPH leu1-32 his3-∆1 ura4 h?* | This work |
| HVP5263 | *pREP41x vps10-GFP:KAN leu1-32 his3-∆1 ura4-∆18 ade6 h+* | This work |
| HVP5264 | *pREP41x+vsl1^+^ vps10-GFP:KAN leu1-32 his3-∆1 ura4-∆18 ade6 h+* | This work |
| HVP5265 | *pREP41x vps10-GFP:KAN stx8::HPH leu1-32 his3-∆1 ura4-∆18 ade6 h+* | This work |
| HVP5266 | *pREP41x+vsl1+ vps10-GFP:KAN stx8::HPH leu1-32 his3-∆1 ura4-∆18 ade6 h+* | This work |
| HVP5276 | *vps10-GFP:KAN vsl1::KAN leu1-32 his3-∆1 ura4-∆18 ade6 h-* | This work |
| HVP5287 | *vsl1::KAN leu1-32 his3-∆1 ura4-∆18 ade6 h-* | This work |
| HVP5288 | *vsl1::KAN leu1-32 his3-∆1 ura4-∆18 ade6 h+* | This work |
| HVP5289 | *Pnda2:GFP-stx8(Δ1-121):Tnda2:NAT leu1-32 his3-∆1 ura4-∆18 h+* | This work |
| HVP5297 | *Pnda2:GFP-stx8:Tnda2:NAT snx4::KAN leu1-32 his3-∆1 ura4-∆18 h?* | This work |
| HVP5326 | *vps28::KAN stx8::HPH vps35-HA:KAN leu1-32 his3-∆1 ura4-∆18 h-* | This work |
| HVP5329 | *Pnda2:GFP-stx8:Tnda2:NAT vps28::KAN stx8::HPH leu1-32 his3-∆1 ura4-∆18 h-* | This work |
| HVP5346 | *vps10-GFP:KAN stx8::HPH vps35::NAT apm3::KAN leu1-32 his3-∆1 ura4-∆18 h?* | This work |
| HVP5368 | *Pnda2:ub:GFP-Cps1:Tnmt1:NAT vsl1::KAN leu1-32 his3-∆1 ura4-∆18 h?* | This work |
| HVP5370 | *Pnda2:GFP-stx8ΔSN:Tnda2:NAT leu1-32 his3-∆1 ura4-∆18 h+* | This work |
| HVP5408 | *Pnda2:GFP-stx8:Tnda2:NAT vps28::KAN stx8::HPH vps35-HA:KAN leu1-32 his3-∆1 ura4-∆18 h?* | This work |
| HVP5409 | *Pnda2:GFP-stx8(Δ1-121):Tnda2:NAT vps35::NAT leu1-32 his3-∆1 ura4-∆18 h?* | This work |
| HVP5419 | *Pnda2:GFP-stx8ΔNC1:Tnda2:NAT vps35::NAT leu1-32 his3-∆1 ura4-∆18 h+* | This work |
| HVP5420 | *Pnda2:GFP-stx8ΔN:Tnda2:NAT vps35::NAT leu1-32 his3-∆1 ura4-∆18 h?* | This work |
| HVP5421 | *Pnda2:GFP-stx8:Tnda2:NAT vps5::KAN leu1-32 his3-∆1 ura4-∆18 h?* | This work |
| HVP5422 | *Pnda2:GFP-stx8:Tnda2:NAT vps17::KAN leu1-32 his3-∆1 ura4-∆18 h?* | This work |
| HVP5428 | *Pnda2:GFP-stx8(Δ122-151):Tnda2:NAT leu1-32 his3-∆1 ura4-∆18 h+* | This work |
| HVP5429 | *Pnda2:GFP-stx8(Δ152-187):Tnda2:NAT leu1-32 his3-∆1 ura4-∆18 h+* | This work |
| HVP5430 | *Pnda2:GFP-stx8(Δ188-224):Tnda2:NAT leu1-32 his3-∆1 ura4-∆18 h+* | This work |
| HVP5440 | *Pnda2:GFP-stx8(Δ122-151):Tnda2:NAT vps28::KAN stx8::HPH leu1-32 his3-∆1 ura4-∆18 h+* | This work |
| HVP5441 | *Pnda2:GFP-stx8(Δ152-187):Tnda2:NAT vps35::NAT leu1-32 his3-∆1 ura4-∆18 h?* | This work |
| HVP5442 | *Pnda2:GFP-stx8(Δ188-224):Tnda2:NAT vps35::NAT leu1-32 his3-∆1 ura4-∆18 h?* | This work |
| HVP5443 | *Pnda2:Cherry-stx8:Tnda2:NAT vps35-GFP:NAT leu1-32 his3-∆1 ura4-∆18 h?* | This work |
| HVP5450 | *Pnda2:GFP-stx8(122-131A):Tnda2:NAT leu1-32 his3-∆1 ura4-∆18 h+* | This work |
| HVP5451 | *Pnda2:GFP-stx8(132-141A):Tnda2:NAT leu1-32 his3-∆1 ura4-∆18 h+* | This work |
| HVP5452 | *Pnda2:GFP-stx8(142-151A):Tnda2:NAT leu1-32 his3-∆1 ura4-∆18 h+* | This work |
| HVP5454 | *Pnda2:GFP-stx8(Δ122-151):Tnda2:NAT vps28::KAN stx8::HPH vps35-HA:KAN leu1-32 his3-∆1 ura4-∆18 h+* | This work |
| HVP5455 | *Pnda2:GFP-stx8:Tnda2:NAT snx3::KAN leu1-32 his3-∆1 ura4-∆18 h?* | This work |
| HVP5459 | *Pnda2:GFP-stx8(D132A):Tnda2:NAT leu1-32 his3-∆1 ura4-∆18 h+* | This work |
| HVP5460 | *Pnda2:GFP-stx8(I133A):Tnda2:NAT leu1-32 his3-∆1 ura4-∆18 h+* | This work |
| HVP5461 | *Pnda2:GFP-stx8(E134A):Tnda2:NAT leu1-32 his3-∆1 ura4-∆18 h+* | This work |
| HVP5462 | *Pnda2:GFP-stx8(M135A):Tnda2:NAT leu1-32 his3-∆1 ura4-∆18 h+* | This work |
| HVP5463 | *Pnda2:GFP-stx8(E136A):Tnda2:NAT leu1-32 his3-∆1 ura4-∆18 h+* | This work |
| HVP5464 | *Pnda2:GFP-stx8(M138A):Tnda2:NAT leu1-32 his3-∆1 ura4-∆18 h+* | This work |
| HVP5465 | *Pnda2:GFP-stx8(Y139A):Tnda2:NAT leu1-32 his3-∆1 ura4-∆18 h+* | This work |
| HVP5466 | *Pnda2:GFP-stx8(V140A):Tnda2:NAT leu1-32 his3-∆1 ura4-∆18 h+* | This work |
| HVP5467 | *Pnda2:GFP-stx8(P141A):Tnda2:NAT leu1-32 his3-∆1 ura4-∆18 h+* | This work |
| HVP5473 | *972 stx8::HPH h-* | This work |
| HVP5474 | *972 stx8(*Δ*SN):HPH h-* | This work |
| HVP5476 | *Pnda2:GFP-stx8(A+G):Tnda2:NAT leu1-32 his3-∆1 ura4-∆18 h+* | This work |
| HVP5477 | *Pnda2:GFP-stx8(A+G):Tnda2:NAT vps35::NAT leu1-32 his3-∆1 ura4-∆18 h+* | This work |
| HVP5478 | *vps10-GFP:KAN snx3::KAN leu1-32 his3-∆1 ura4-∆18 ade6 h-* | This work |
| HVP5481 | *stx8(*Δ*SN):HPH Cherry-FIVE:NAT leu1-32 ura4-∆18 h^?^* | This work |
| HVP5484 | *Pnda2:GFP-stx8(122-131A):Tnda2:NAT vps28::KAN stx8::HPH vps35-HA:KAN leu1-32 his3-∆1 ura4-∆18 h?* | This work |
| HVP5485 | *Pnda2:GFP-stx8(132-141A):Tnda2:NAT vps28::KAN stx8::HPH vps35-HA:KAN leu1-32 his3-∆1 ura4-∆18 h?* | This work |
| HVP5486 | *Pnda2:GFP-stx8(142-151A):Tnda2:NAT vps28::KAN stx8::HPH vps35-HA:KAN leu1-32 his3-∆1 ura4-∆18 h?* | This work |
| HVP5492 | *vps10-GFP:KAN stx8(ΔSN):KAN leu1-32 his3-∆1 ura4-∆18 ade6 h?* | This work |
| HVP5495 | *Pnda2:GFP-stx8(G130A):Tnda2:NAT leu1-32 his3-∆1 ura4-∆18 h+* | This work |
| HVP5525 | *Pnda2:ub:GFP-Cps1:Tnmt1:NAT psp3::NAT isp6::ura4+ leu1-32 his3-∆1 ura4-∆18 h-* | This work |
| HVP5526 | *Pnda2:ub:GFP-Cps1:Tnmt1:NAT stx8::HPH psp3::NAT isp6::ura4+ leu1-32 his3-∆1 ura4-∆18 h-* | This work |
| HVP5527 | *vps10-GFP:KAN stx8::HPH Cherry-stx8:NAT* | This work |
| HVP5528 | *Pnda2:GFP-stx8:Tnda2:NAT cfr1-RFP:ura4 cfr1::his3 vps35::NAT leu1-32 his3-∆1 ura4-∆18 h?* | This work |
| HVP5536 | *Pnda2:VN_YFP_-stx8:Tnda2:NAT leu1-32 his3-∆1 ura4-∆18 h-* | This work |
| HVP5537 | *Snx3-VC_YFP_:KAN leu1-32 his3-∆1 ura4-∆18 h+* | This work |
| HVP5538 | *Pnda2:VN_YFP_-stx8:Tnda2:NAT Snx3-VC_YFP_:KAN leu1-32 his3-∆1 ura4-∆18* | This work |
|  |  |  |
| *Saccharomyces*  *cerevisiae*  AH109 | *MATa, trp1-901, leu2-3, 112, ura3-52, his3-200, gal4Δ, gal80Δ, LYS2::GAL1_UAS_-GAL1_TATA_-HIS3, MEL1, GAL2_UAS_-GAL2_TATA_-ADE2, URA3::MEL1_UAS_-MEL1_TATA_-LacZ* | Clontech |
