# Supplementary material for: Analysis of the SNARE Stx8 recycling reveals that the retromer-sorting motif has undergone evolutionary divergence
Source: PLoS Genet. 2021 Mar 31;17(3):e1009463. doi: 10.1371/journal.pgen.1009463 (PMC8041195; doi:10.1371/journal.pgen.1009463)
Supplement: S1 File — Amino acidic sequence and information about the construction of the Stx8 variant (DOCX) [file pgen.1009463.s009.docx]

**S1 File. Amino acidic sequence and information about the construction of Stx8 variants**

**-Related to Fig. 2**

***stx8(ΔN)***

Schematic, not-to scale representation of the cassette constructed to integrate *stx8(ΔSN)* mutation into the *stx8^+^* locus under the control of its endogenous promoter. The light-blue line corresponds to the Stx8 variant (the yellow line denotes the deleted region). The red, black and green lines correspond to the *nda2^+^* terminator, KANMX6 and *stx8^+^* 3’ UTR sequences, respectively.

Three DNA fragments were PCR-amplified using the following primers:

**1. Fragment Fsv1:KAN**. Amplified from the pINTH:P*nda2*^+^:*stx8(ΔSN)*:T*nda2*^+^:*NATMX6* plasmid

**Fsv1_Mlu-F:** tatatataACGCGT**ATG**TCAAATTTGCTTCTGATCATTG

**KN:Tnda-R:**  caagctaaacagatctggcgcgccttaattaacCCACAGAAAGGAGAAATTGAAATTTTACTGGTGG

**2. Fragment Fsv1:KAN:3’**. Amplified from a plasmid bearing *KANMX6*

**Tnda:KN-F**: CCACCAGTAAAATTTCAATTTCTCCTTTCTGTGGgttaattaaggcgcgccagatctgtttagcttg

**3’:KN-R:** GTTTATTGTTTACTGCCCTGAATCTAAAAATTTTcataggccactagtggatctgatatcatcg

**3. Fragment KAN:3’UTR**. Amplified from genomic DNA

**KN:3’-F:** cgatgatatcagatccactagtggcctatgAAAATTTTTAGATTCAGGGCAGTAAACAATAAAC

**Fsv1-R2:** CTATTTGTTTCTTAATGAAATGCATC

These fragments were fused in a PCR reaction using primers Fsv1_Mlu-F and Fsv1-R2

**-Related to Fig. 4**

Sequences in red correspond to the restriction sites. Sequences in blue were introduced to facilitate enzymatic digestion and are lost in the final construct.

**Stx8**

MSNLLLIIDSVSQKIRDRRKLEEFGQNPDEEIESSLKDVRQELQKLNEEQSRLEKNAQIPEYRVRESEAFLIRMQRRLESAEEEFEKQRRASSIPADGTSAFSANPQVASTNNKLTPLPSLQKTTSSSEGSDIEMEAMYPVDGNDPDPINVNVLAQMHQQMLNEQEESLGGIEASVQRQKRMGYAMNTELSEQNVLLDNMNNDADRIERRFDHAKNRLNKVSRKAKQYPRCFIILLLCALLLLVASI

Primers used for amplification

**Fsv1_Mlu-F**: tatatataACGCGT**ATG**TCAAATTTGCTTCTGATCATTG

**Fsv1_Sal-R**: tatatataGTCGAC**CTA**AATGGAAGCCACAAGTAATAAC

**Stx8(ΔN)**

NPDEEIESSLKDVRQELQKLNEEQSRLEKNAQIPEYRVRESEAFLIRMQRRLESAEEEFEKQRRASSIPADGTSAFSANPQVASTNNKLTPLPSLQKTTSSSEGSDIEMEAMYPVDGNDPDPINVNVLAQMHQQMLNEQEESLGGIEASVQRQKRMGYAMNTELSEQNVLLDNMNNDADRIERRFDHAKNRLNKVSRKAKQYPRCFIILLLCALLLLVASI

Primers used for PCR amplification

**Fsv1DN-F**: tatatataACGCGT**AATCCCGATGAAGAAATTGAGTCT**

**Fsv1_Sal-R**: tatatataGTCGAC**CTA**AATGGAAGCCACAAGTAATAAC

**Stx8(ΔNC1)**

YRVRESEAFLIRMQRRLESAEEEFEKQRRASSIPADGTSAFSANPQVASTNNKLTPLPSLQKTTSSSEGSDIEMEAMYPVDGNDPDPINVNVLAQMHQQMLNEQEESLGGIEASVQRQKRMGYAMNTELSEQNVLLDNMNNDADRIERRFDHAKNRLNKVSRKAKQYPRCFIILLLCALLLLVASI

Primers used for amplification

**Fsv1DNC1-F**: tatatataACGCGT**TACAGAGTACGAGAATCCGAAGCA**

**Fsv1_Sal-R**: tatatataGTCGAC**CTA**AATGGAAGCCACAAGTAATAAC

**Stx8(ΔNCC)**

IPADGTSAFSANPQVASTNNKLTPLPSLQKTTSSSEGSDIEMEAMYPVDGNDPDPINVNVLAQMHQQMLNEQEESLGGIEASVQRQKRMGYAMNTELSEQNVLLDNMNNDADRIERRFDHAKNRLNKVSRKAKQYPRCFIILLLCALLLLVASI

Primers used for amplification

**Fsv1DNCC-F**: tatatataACGCGT**ATTCCTGCTGACGGAACAAGTGCC**

**Fsv1_Sal-R**: tatatataGTCGAC**CTA**AATGGAAGCCACAAGTAATAAC

**Stx8(Δ1-121)**

QKTTSSSEGSDIEMEAMYPVDGNDPDPINVNVLAQMHQQMLNEQEESLGGIEASVQRQKRMGYAMNTELSEQNVLLDNMNNDADRIERRFDHAKNRLNKVSRKAKQYPRCFIILLLCALLLLVASI

Primers used for amplification

**Fsv1_122-F**: tatatataACGCGT**CAAAAAACGACTTCGAGTAGTGAG**

**Fsv1_Sal-R**: tatatataGTCGAC**CTA**AATGGAAGCCACAAGTAATAAC

**Stx8(SNTM)**

NVLAQMHQQMLNEQEESLGGIEASVQRQKRMGYAMNTELSEQNVLLDNMNNDADRIERRFDHAKNRLNKVSRKAKQYPRCFIILLLCALLLLVASI

Primers used for amplification

**Fsv1_TMSN-F**: tatatataACGCGT**AACGTTTTGGCTCAAATGCATCAG**

**Fsv1_Sal-R**: tatatataGTCGAC**CTA**AATGGAAGCCACAAGTAATAAC

**Stx8(TM)**

AKQYPRCFIILLLCALLLLVASI

Primers used for amplification

**Fsv1_TM-F**: tatatataACGCGT**GCTAAACAGTATCCCAGATGCTTT**

**Fsv1_Sal-R**: tatatataGTCGAC**CTA**AATGGAAGCCACAAGTAATAAC

**-Related to Fig. 5**

**Stx8(Δ122-151)**

MSNLLLIIDSVSQKIRDRRKLEEFGQNPDEEIESSLKDVRQELQKLNEEQSRLEKNAQIPEYRVRESEAFLIRMQRRLESAEEEFEKQRRASSIPADGTSAFSANPQVASTNNKLTPLPSLNVLAQMHQQMLNEQEESLGGIEASVQRQKRMGYAMNTELSEQNVLLDNMNNDADRIERRFDHAKNRLNKVSRKAKQYPRCFIILLLCALLLLVASI

Two DNA fragments were obtained by PCR with the following primers:

5’Frafgment:

**Fsv1_Mlu-F**: tatatataACGCGT**ATG**TCAAATTTGCTTCTGATCATTG

Fsv1_L2-Int1-R: GCTGATGCATTTGAGCCAAAACGTT**TAAAGAAGGAAGGGGAGTTAAC**

3’Fragment:

Fsv1_L3-Int1-F: **GTTAACTCCCCTTCCTTCTTTA**AACGTTTTGGCTCAAATGCATCAGC

**Fsv1_Sal-R**: tatatataGTCGAC**CTA**AATGGAAGCCACAAGTAATAAC

These 2 fragments were fused in a PCR reaction with primers Fsv1_Mlu-F and Fsv1_Sal-R

**Stx8(122-131)A**

MSNLLLIIDSVSQKIRDRRKLEEFGQNPDEEIESSLKDVRQELQKLNEEQSRLEKNAQIPEYRVRESEAFLIRMQRRLESAEEEFEKQRRASSIPADGTSAFSANPQVASTNNKLTPLPSL**AAAAAAAAAA**DIEMEAMYPVDGNDPDPINVNVLAQMHQQMLNEQEESLGGIEASVQRQKRMGYAMNTELSEQNVLLDNMNNDADRIERRFDHAKNRLNKVSRKAKQYPRCFIILLLCALLLLVASI

Two DNA fragments were obtained by PCR with the following primers:

5’Fragment:

**Fsv1_Mlu-F**: tatatataACGCGT**ATG**TCAAATTTGCTTCTGATCATTG

**Fsv1A(122-131)-R**: atggcctccatctcaatatctgccgctgcggcagctgccgctgcggcagctaaagaaggaaggggagttaacttattatt

3’ Fragment:

**Fsv1A(122-131)-F**:

taactccccttccttctttagctgccgcagcggcagctgccgcagcggcagatattgagatggaggccatgtatccggtt

**Fsv1_Sal-R**: tatatataGTCGAC**CTA**AATGGAAGCCACAAGTAATAAC

These 2 fragments were fused in a PCR reaction with primers Fsv1_Mlu-F and Fsv1_Sal-R

**Stx8(132-141)A**

MSNLLLIIDSVSQKIRDRRKLEEFGQNPDEEIESSLKDVRQELQKLNEEQSRLEKNAQIPEYRVRESEAFLIRMQRRLESAEEEFEKQRRASSIPADGTSAFSANPQVASTNNKLTPLPSLQKTTSSSEGS**AAAAAAAAAA**DGNDPDPINVNVLAQMHQQMLNEQEESLGGIEASVQRQKRMGYAMNTELSEQNVLLDNMNNDADRIERRFDHAKNRLNKVSRKAKQYPRCFIILLLCALLLLVASI

Two DNA fragments were obtained by PCR with the following primers:

5’Fragment:

**Fsv1_Mlu-F**: tatatataACGCGT**ATG**TCAAATTTGCTTCTGATCATTG

**Fsv1A(132-141)-R: ggatcagggtcattgccatcagccgctgcggcagcagccgctgcggcagcagatccctcactactcgaagtcgttttttg**

3’ Fragment:

**Fsv1A(132-141)-F**:

cttcgagtagtgagggatctgctgccgcagcggctgctgccgcagcggctgatggcaatgaccctgatcccataaacgta

**Fsv1_Sal-R**: tatatataGTCGAC**CTA**AATGGAAGCCACAAGTAATAAC

These 2 fragments were fused in a PCR reaction with primers Fsv1_Mlu-F and Fsv1_Sal-R

**Stx8(142-151)A**

MSNLLLIIDSVSQKIRDRRKLEEFGQNPDEEIESSLKDVRQELQKLNEEQSRLEKNAQIPEYRVRESEAFLIRMQRRLESAEEEFEKQRRASSIPADGTSAFSANPQVASTNNKLTPLPSLQKTTSSSEGSDIEMEAMYPV**AAAAAAAAAA**NVLAQMHQQMLNEQEESLGGIEASVQRQKRMGYAMNTELSEQNVLLDNMNNDADRIERRFDHAKNRLNKVSRKAKQYPRCFIILLLCALLLLVASI

Two DNA fragments were obtained by PCR with the following primers:

5’Fragment:

**Fsv1_Mlu-F**: tatatataACGCGT**ATG**TCAAATTTGCTTCTGATCATTG

**Fsv1A(142-151)-R:** tgcatttgagccaaaacgtttgcagcagctgcagccgctgcggctgcagcaaccggatacatggcctccatctcaatatc

3’ Fragment:

**Fsv1A(142-151)-F**: **tggaggccatgtatccggttgctgcagccgcagcggctgCAGCTGctgcaAACGTTTTGGCTCAAATGCATCAGCAAATG**

**Fsv1_Sal-R**: tatatataGTCGAC**CTA**AATGGAAGCCACAAGTAATAAC

These 2 fragments were fused in a PCR reaction with primers Fsv1_Mlu-F and Fsv1_Sal-R

**-Related to Fig. 6**

**Stx8(D132A)**

MSNLLLIIDSVSQKIRDRRKLEEFGQNPDEEIESSLKDVRQELQKLNEEQSRLEKNAQIPEYRVRESEAFLIRMQRRLESAEEEFEKQRRASSIPADGTSAFSANPQVASTNNKLTPLPSLQKTTSSSEGS**A**IEMEAMYPVDGNDPDPINVNVLAQMHQQMLNEQEESLGGIEASVQRQKRMGYAMNTELSEQNVLLDNMNNDADRIERRFDHAKNRLNKVSRKAKQYPRCFIILLLCALLLLVASI

Two DNA fragments were obtained by PCR with the following primers:

5’Fragment:

**Fsv1_Mlu-F**: tatatataACGCGT**ATG**TCAAATTTGCTTCTGATCATTG

**Fsv1A132-R:** GGATACATGGCCTCCATCTCAATagcAGATCCCTCACTACTCGAAGTCG

3’ Fragment:

**Fsv1A132-F**: CGACTTCGAGTAGTGAGGGATCTgctATTGAGATGGAGGCCATGTATCC

**Fsv1_Sal-R**: tatatataGTCGAC**CTA**AATGGAAGCCACAAGTAATAAC

These 2 fragments were fused in a PCR reaction with primers Fsv1_Mlu-F and Fsv1_Sal-R

**Stx8(I133A)**

MSNLLLIIDSVSQKIRDRRKLEEFGQNPDEEIESSLKDVRQELQKLNEEQSRLEKNAQIPEYRVRESEAFLIRMQRRLESAEEEFEKQRRASSIPADGTSAFSANPQVASTNNKLTPLPSLQKTTSSSEGSD**A**EMEAMYPVDGNDPDPINVNVLAQMHQQMLNEQEESLGGIEASVQRQKRMGYAMNTELSEQNVLLDNMNNDADRIERRFDHAKNRLNKVSRKAKQYPRCFIILLLCALLLLVASI

Two DNA fragments were obtained by PCR with the following primers:

5’Fragment:

**Fsv1_Mlu-F**: tatatataACGCGT**ATG**TCAAATTTGCTTCTGATCATTG

**Fsv1A133-R:** ACCGGATACATGGCCTCCATCTCagcATCAGATCCCTCACTACTCGAAG

3’ Fragment:

**Fsv1A133-F**: CTTCGAGTAGTGAGGGATCTGATgctGAGATGGAGGCCATGTATCCGGT

**Fsv1_Sal-R**: tatatataGTCGAC**CTA**AATGGAAGCCACAAGTAATAAC

These 2 fragments were fused in a PCR reaction with primers Fsv1_Mlu-F and Fsv1_Sal-R

**Stx8(E134A)**

MSNLLLIIDSVSQKIRDRRKLEEFGQNPDEEIESSLKDVRQELQKLNEEQSRLEKNAQIPEYRVRESEAFLIRMQRRLESAEEEFEKQRRASSIPADGTSAFSANPQVASTNNKLTPLPSLQKTTSSSEGSDI**A**MEAMYPVDGNDPDPINVNVLAQMHQQMLNEQEESLGGIEASVQRQKRMGYAMNTELSEQNVLLDNMNNDADRIERRFDHAKNRLNKVSRKAKQYPRCFIILLLCALLLLVASI

Two DNA fragments were obtained by PCR with the following primers:

5’Fragment:

**Fsv1_Mlu-F**: tatatataACGCGT**ATG**TCAAATTTGCTTCTGATCATTG

**Fsv1A34-R:** TCAACCGGATACATGGCCTCCATcgcAATATCAGATCCCTCACTACTCG

3’ Fragment:

**Fsv1A134-F**: CGAGTAGTGAGGGATCTGATATTgcgATGGAGGCCATGTATCCGGTTGA

**Fsv1_Sal-R**: tatatataGTCGAC**CTA**AATGGAAGCCACAAGTAATAAC

These 2 fragments were fused in a PCR reaction with primers Fsv1_Mlu-F and Fsv1_Sal-R

**Stx8(M135A)**

MSNLLLIIDSVSQKIRDRRKLEEFGQNPDEEIESSLKDVRQELQKLNEEQSRLEKNAQIPEYRVRESEAFLIRMQRRLESAEEEFEKQRRASSIPADGTSAFSANPQVASTNNKLTPLPSLQKTTSSSEGSDIE**A**EAMYPVDGNDPDPINVNVLAQMHQQMLNEQEESLGGIEASVQRQKRMGYAMNTELSEQNVLLDNMNNDADRIERRFDHAKNRLNKVSRKAKQYPRCFIILLLCALLLLVASI

Two DNA fragments were obtained by PCR with the following primers:

5’Fragment:

**Fsv1_Mlu-F**: tatatataACGCGT**ATG**TCAAATTTGCTTCTGATCATTG

**Fsv1A135-R:** CCATCAACCGGATACATGGCCTCcgcCTCAATATCAGATCCCTCACTAC

3’ Fragment:

**Fsv1A135-F**: GTAGTGAGGGATCTGATATTGAGgcgGAGGCCATGTATCCGGTTGATGG

**Fsv1_Sal-R**: tatatataGTCGAC**CTA**AATGGAAGCCACAAGTAATAAC

These 2 fragments were fused in a PCR reaction with primers Fsv1_Mlu-F and Fsv1_Sal-R

**Stx8(E136A)**

MSNLLLIIDSVSQKIRDRRKLEEFGQNPDEEIESSLKDVRQELQKLNEEQSRLEKNAQIPEYRVRESEAFLIRMQRRLESAEEEFEKQRRASSIPADGTSAFSANPQVASTNNKLTPLPSLQKTTSSSEGSDIEM**A**AMYPVDGNDPDPINVNVLAQMHQQMLNEQEESLGGIEASVQRQKRMGYAMNTELSEQNVLLDNMNNDADRIERRFDHAKNRLNKVSRKAKQYPRCFIILLLCALLLLVASI

Two DNA fragments were obtained by PCR with the following primers:

5’Fragment:

**Fsv1_Mlu-F**: tatatataACGCGT**ATG**TCAAATTTGCTTCTGATCATTG

**Fsv1A136-R:** TTGCCATCAACCGGATACATGGCcgcCATCTCAATATCAGATCCCTCAC

3’ Fragment:

**Fsv1A136-F**: GTGAGGGATCTGATATTGAGATGgcgGCCATGTATCCGGTTGATGGCAA

**Fsv1_Sal-R**: tatatataGTCGAC**CTA**AATGGAAGCCACAAGTAATAAC

These 2 fragments were fused in a PCR reaction with primers Fsv1_Mlu-F and Fsv1_Sal-R

**Stx8(M138A)**

MSNLLLIIDSVSQKIRDRRKLEEFGQNPDEEIESSLKDVRQELQKLNEEQSRLEKNAQIPEYRVRESEAFLIRMQRRLESAEEEFEKQRRASSIPADGTSAFSANPQVASTNNKLTPLPSLQKTTSSSEGSDIEMEA**A**YPVDGNDPDPINVNVLAQMHQQMLNEQEESLGGIEASVQRQKRMGYAMNTELSEQNVLLDNMNNDADRIERRFDHAKNRLNKVSRKAKQYPRCFIILLLCALLLLVASI

Two DNA fragments were obtained by PCR with the following primers:

5’Fragment:

**Fsv1_Mlu-F**: tatatataACGCGT**ATG**TCAAATTTGCTTCTGATCATTG

**Fsv1A138-R:** GGGTCATTGCCATCAACCGGATAcgcGGCCTCCATCTCAATATCAGATC

3’ Fragment:

**Fsv1A138-F**: GATCTGATATTGAGATGGAGGCCgcgTATCCGGTTGATGGCAATGACCC

**Fsv1_Sal-R**: tatatataGTCGAC**CTA**AATGGAAGCCACAAGTAATAAC

These 2 fragments were fused in a PCR reaction with primers Fsv1_Mlu-F and Fsv1_Sal-R

**Stx8(Y139A)**

MSNLLLIIDSVSQKIRDRRKLEEFGQNPDEEIESSLKDVRQELQKLNEEQSRLEKNAQIPEYRVRESEAFLIRMQRRLESAEEEFEKQRRASSIPADGTSAFSANPQVASTNNKLTPLPSLQKTTSSSEGSDIEMEAM**A**PVDGNDPDPINVNVLAQMHQQMLNEQEESLGGIEASVQRQKRMGYAMNTELSEQNVLLDNMNNDADRIERRFDHAKNRLNKVSRKAKQYPRCFIILLLCALLLLVASI

Two DNA fragments were obtained by PCR with the following primers:

5’Fragment:

**Fsv1_Mlu-F**: tatatataACGCGT**ATG**TCAAATTTGCTTCTGATCATTG

**Fsv1A139-R:** TCAGGGTCATTGCCATCAACCGGagcCATGGCCTCCATCTCAATATCAG

3’ Fragment:

**Fsv1A139-F**: CTGATATTGAGATGGAGGCCATGgctCCGGTTGATGGCAATGACCCTGA

**Fsv1_Sal-R**: tatatataGTCGAC**CTA**AATGGAAGCCACAAGTAATAAC

These 2 fragments were fused in a PCR reaction with primers Fsv1_Mlu-F and Fsv1_Sal-R

**Stx8(P140A)**

MSNLLLIIDSVSQKIRDRRKLEEFGQNPDEEIESSLKDVRQELQKLNEEQSRLEKNAQIPEYRVRESEAFLIRMQRRLESAEEEFEKQRRASSIPADGTSAFSANPQVASTNNKLTPLPSLQKTTSSSEGSDIEMEAMY**A**VDGNDPDPINVNVLAQMHQQMLNEQEESLGGIEASVQRQKRMGYAMNTELSEQNVLLDNMNNDADRIERRFDHAKNRLNKVSRKAKQYPRCFIILLLCALLLLVASI

Two DNA fragments were obtained by PCR with the following primers:

5’Fragment:

**Fsv1_Mlu-F**: tatatataACGCGT**ATG**TCAAATTTGCTTCTGATCATTG

**Fsv1A140-R:** GGATCAGGGTCATTGCCATCAACcgcATACATGGCCTCCATCTCAATAT

3’ Fragment:

**Fsv1A140-F**: ATATTGAGATGGAGGCCATGTATgcgGTTGATGGCAATGACCCTGATCC

**Fsv1_Sal-R**: tatatataGTCGAC**CTA**AATGGAAGCCACAAGTAATAAC

These 2 fragments were fused in a PCR reaction with primers Fsv1_Mlu-F and Fsv1_Sal-R

**Stx8(V141A)**

MSNLLLIIDSVSQKIRDRRKLEEFGQNPDEEIESSLKDVRQELQKLNEEQSRLEKNAQIPEYRVRESEAFLIRMQRRLESAEEEFEKQRRASSIPADGTSAFSANPQVASTNNKLTPLPSLQKTTSSSEGSDIEMEAMYP**A**DGNDPDPINVNVLAQMHQQMLNEQEESLGGIEASVQRQKRMGYAMNTELSEQNVLLDNMNNDADRIERRFDHAKNRLNKVSRKAKQYPRCFIILLLCALLLLVASI

Two DNA fragments were obtained by PCR with the following primers:

5’Fragment:

**Fsv1_Mlu-F**: tatatataACGCGT**ATG**TCAAATTTGCTTCTGATCATTG

**Fsv1A141-R:** ATGGGATCAGGGTCATTGCCATCagcCGGATACATGGCCTCCATCTCAA

3’ Fragment:

**Fsv1A141-F**: TTGAGATGGAGGCCATGTATCCGgctGATGGCAATGACCCTGATCCCAT

**Fsv1_Sal-R**: tatatataGTCGAC**CTA**AATGGAAGCCACAAGTAATAAC

These 2 fragments were fused in a PCR reaction with primers Fsv1_Mlu-F and Fsv1_Sal-R

**Stx8(G130A)**

MSNLLLIIDSVSQKIRDRRKLEEFGQNPDEEIESSLKDVRQELQKLNEEQSRLEKNAQIPEYRVRESEAFLIRMQRRLESAEEEFEKQRRASSIPADGTSAFSANPQVASTNNKLTPLPSLQKTTSSSE**A**SDIEMEAMYPVDGNDPDPINVNVLAQMHQQMLNEQEESLGGIEASVQRQKRMGYAMNTELSEQNVLLDNMNNDADRIERRFDHAKNRLNKVSRKAKQYPRCFIILLLCALLLLVASI

Two DNA fragments were obtained by PCR with the following primers:

5’Fragment:

**Fsv1_Mlu-F**: tatatataACGCGT**ATG**TCAAATTTGCTTCTGATCATTG

**Fsv1A130-R:** CGGATACATGGCCTCCATCTCAATATCAGATgCCTCACTACTCGAAGTCGTTTTTTG

3’ Fragment:

**Fsv1A130-F**: CAAAAAACGACTTCGAGTAGTGAGGcATCTGATATTGAGATGGAGGCCATGTATCCG

**Fsv1_Sal-R**: tatatataGTCGAC**CTA**AATGGAAGCCACAAGTAATAAC

These 2 fragments were fused in a PCR reaction with primers Fsv1_Mlu-F and Fsv1_Sal-R

**-Related to Fig. 7**

**Stx8(ΔSN)**

MSNLLLIIDSVSQKIRDRRKLEEFGQNPDEEIESSLKDVRQELQKLNEEQSRLEKNAQIPEYRVRESEAFLIRMQRRLESAEEEFEKQRRASSIPADGTSAFSANPQVASTNNKLTPLPSLQKTTSSSEGSDIEMEAMYPVDGNDPDPINVRLNKVSRKAKQYPRCFIILLLCALLLLVASI

Primer used for site-directed mutagenesis

CTTTCGGCTGACCTTGTTTAATCGCTGCAGTACGTTTATGGGATCAGGGTCATTGCC

**Stx8(Δ152-187)**

MSNLLLIIDSVSQKIRDRRKLEEFGQNPDEEIESSLKDVRQELQKLNEEQSRLEKNAQIPEYRVRESEAFLIRMQRRLESAEEEFEKQRRASSIPADGTSAFSANPQVASTNNKLTPLPSLQKTTSSSEGSDIEMEAMYPVDGNDPDPINVTELSEQNVLLDNMNNDADRIERRFDHAKNRLNKVSRKAKQYPRCFIILLLCALLLLVASI

Two DNA fragments were obtained by PCR with the following primers:

5’Fragment:

**Fsv1_Mlu-F**: tatatataACGCGT**ATG**TCAAATTTGCTTCTGATCATTG

**Fsv1D152-187-R**: AACATTTTGTTCGGACAATTCTGTTACGTTTATGGGATCAGGGTC

3’ Fragment:

**Fsv1D152-187-F**: AATGACCCTGATCCCATAAACGTAACAGAATTGTCCGAACAAAATGTT

**Fsv1_Sal-R**: tatatataGTCGAC**CTA**AATGGAAGCCACAAGTAATAAC

These 2 fragments were fused in a PCR reaction with primers Fsv1_Mlu-F and Fsv1_Sal-R

**Stx8(Δ188-224)**

MSNLLLIIDSVSQKIRDRRKLEEFGQNPDEEIESSLKDVRQELQKLNEEQSRLEKNAQIPEYRVRESEAFLIRMQRRLESAEEEFEKQRRASSIPADGTSAFSANPQVASTNNKLTPLPSLQKTTSSSEGSDIEMEAMYPVDGNDPDPINVNVLAQMHQQMLNEQEESLGGIEASVQRQKRMGYAMNAKQYPRCFIILLLCALLLLVASI

Two DNA fragments were obtained by PCR with the following primers:

5’Fragment:

**Fsv1_Mlu-F**: tatatataACGCGT**ATG**TCAAATTTGCTTCTGATCATTG

**Fsv1D188-224-R:** AAAGCATCTGGGATACTGTTTAGCATTCATTGCATATCCCATCCGC

3’ Fragment:

**Fsv1D188-224-F**: AAGCGGATGGGATATGCAATGAATGCTAAACAGTATCCCAGATGC

**Fsv1_Sal-R**: tatatataGTCGAC**CTA**AATGGAAGCCACAAGTAATAAC

These 2 fragments were fused in a PCR reaction with primers Fsv1_Mlu-F and Fsv1_Sal-R

**Stx8(A+G)**

MSNLLLIIDSVSQKIRDRRKLEEFGQNPDEEIESSLKDVRQELQKLNEEQSRLEKNAQIPEYRVRESEAFLIRMQRRLESAEEEFEKQRRASSIPADGTSAFSANPQVASTNNKLTPLPSLQKTTSSSEGSDIEMEAMYPVDGNDPDPINV**AAGGAAGAGGAAAAGAGAAAGGAAAAGGAAAGAGAA**AKQYPRCFIILLLCALLLLVASI

The following DNA fragment was purchased from IDT Technologies, as a Custom Gene, and cloned as an *Mlu*I/*Sal*I insert:

actaattcaACGCGT**ATG**TCAAATTTGCTTCTGATCATTGACTCGGTTTCACAAAAAATAAGAGACAGGAGGAAGTTGGAAGAGTTTGGACAGAATCCCGATGAAGAAATTGAGTCTTCATTGAAAGATGTTCGGCAAGAATTGCAAAAACTTAATGAGGAGCAATCGCGATTAGAGAAAAATGCACAAATCCCAGAGTACAGAGTACGAGAATCCGAAGCATTTCTTATTAGAATGCAAAGGAGGTTAGAGTCTGCTGAGGAAGAGTTTGAGAAGCAGCGGCGTGCATCCTCAATTCCTGCTGACGGAACAAGTGCCTTTTCTGCAAACCCGCAAGTTGCAAGTACGAATAATAAGTTAACTCCCCTTCCTTCTTTACAAAAAACGACTTCGAGTAGTGAGGGATCTGATATTGAGATGGAGGCCATGTATCCGGTTGATGGCAATGACCCTGATCCCATAAACGTAgctgcaggaggagctgctggagcaggtggagctgcagctgcaggagcaggtgccgctgcaggcggagctgctgcagctggtggagctgctgccggagctggagctgcaGCTAAACAGTATCCCAGATGCTTTATTATTTTGCTCCTTTGTGCGTTGTTATTACTTGTGGCTTCCATT**TAG**GTCGACtattcatga

Sequences in red correspond to the restriction sites. Sequences in blue were introduced to facilitate enzymatic digestion and are lost in the final construct.
