# Supplementary material for: Analysis of the SNARE Stx8 recycling reveals that the retromer-sorting motif has undergone evolutionary divergence
Source: PLoS Genet. 2021 Mar 31;17(3):e1009463. doi: 10.1371/journal.pgen.1009463 (PMC8041195; doi:10.1371/journal.pgen.1009463)
Supplement: S3 File — The indicated sequence alignments were used to generate weblogos (DOCX) [file pgen.1009463.s011.docx]

**S3 File. Sequence alignments used to generate graphics in** [**http://weblogo.threeplusone.com/**](http://weblogo.threeplusone.com/)**. Related to Fig. 8**

1. ClustalW alignment of Stx8 from different *Schizosaccharomyces* strains.

*S. japonicus* NTSAVTSISNTYGATMPDNDSAIELGTFTPNHAGTADSNAAQDIDVEALHNIHTQMLLEQ 180

*S. pombe* TNNKLTPLPSLQKTTSSSEGSD**IEM**EA**M**Y-----PVDGNDPDPINVNVLAQMHQQMLNEQ 165

*S. octosporus* KQA-RAPLPS----DVTSNYSDIEMESIH-----QFDGNDPNPVDINLLSQMHEQMLNEQ 154

*S. cryophilus* KQAAAAPLTS----DATSNKSDIEMESIY-----QFEGNDPNPIDVNLLTQMHQQMLNEQ 155

. : : . .: * **: :: :.* : :::: * ::* *** **

1. Sequences from other fungi. ClustalW alignment of the Stx8 residues that were aligned to the *S. pombe* GSDIEMEAMYPV sequence when the whole protein sequences were submitted

*Sch. octosporus* YSDIEMESIHQF-

*Sch. cryophilus* KSDIEMESIYQF-

*Sch. pombe* GSDIEMEAMYPV-

*Sch. japonicus* DSAIELGTFTPN-

*Neurospora crassa* NSDLEAQASAAN-

*Neurospora tetrasperma* NSDLEAQASTAN-

*Aspergillus oryzae* EADLERQNLFQP-

*Aspergillus flavus* EADLERQNLFQP-

*Aspergillus clavatus* EADLNRRNLFQP-

*Arthroderma otae* EHDPNREALLRP-

*Arthroderma gypseum* EHDPNRDALLRP-

*Botryotinia fuckeliana* ASDPARAALFPY-

*Fomitiporia mediterranea* EPPQETEQTRPL-

*Schizophyllum commune* AKPQETEETRPL-

*Fomitopsis pinicola* -VRVFGAAVKPQE

*Yarrowia lypolitica* GSGRKGRVLGE--

*Lachancea thermotolerans* LPTTNSQALKPI-

*Myceliophthora thermophila* AAHIAAQGLSNA-

*Schizophyllum commune2* VPSRSPEPMTPY-
