# Supplementary material for: Analysis of the SNARE Stx8 recycling reveals that the retromer-sorting motif has undergone evolutionary divergence
Source: PLoS Genet. 2021 Mar 31;17(3):e1009463. doi: 10.1371/journal.pgen.1009463 (PMC8041195; doi:10.1371/journal.pgen.1009463)
Supplement: S4 File — The indicated sequences were used to screen for the presence of potential Snx3-retromer sorting motifs using ScanProsite (DOCX) [file pgen.1009463.s012.docx]

**S4 File. Syntaxin8 sequences used to screen for putative motifs recognized by the Snx3-retromer using ScanProsite** (https://prosite.expasy.org/scanprosite/). **Related to Fig. 8**

**Input sequences: Syntaxin 8 genes**

>*Schizosaccharomyces pombe*

MSNLLLIIDSVSQKIRDRRKLEEFGQNPDEEIESSLKDVRQELQKLNEEQSRLEKNAQIPEYRVRESEAFLIRMQRRLESAEEEFEKQRRASSIPADGTSAFSANPQVASTNNKLTPLPSLQKTTSSSEGSDIEMEAMYPVDGNDPDPINVNVLAQMHQQMLNEQEESLGGIEASVQRQKRMGYAMNTELSEQNVLLDNMNNDADRIERRFDHAKNRLNKVSRKAKQYPRCFIILLLCALLLLVASI

>*Schizosaccharomyces octosporus*

MSKLLLLVDSTTKKINDYERLVEFGNSPDEEIDVSLRDIHKQLQQLNEEQTRLEQNAQIPEYKVRESEAFLIRIQRRVESAEEMLLKKRNDATQGPKIHSSYASKQARAPLPSDVTSNYSDIEMESIHQFDGNDPNPVDINLLSQMHEQMLNEQEESLGGIESSVRRQKQMGYLMNNELEEHNDLIDRMGEDADRVDKRLGLARNRLQKVTRKAKQYPRCCIILFLCFLLILVCFI

>*Schizosaccharomyces cryophilus*

MSKLLLLVDSTTKKINDYEKLIEFGNSPDAEIDSSLQDIRRQLQSLSQEQTRLEQNAQIPEYRLRESEAYLIRMQRRVESAEETLLRKRNAASQGPSFQSSYSSKQAAAAPLTSDATSNKSDIEMESIYQFEGNDPNPIDVNLLTQMHQQMLNEQEESLGGIEASVRKQKQLGYLMNDELEEHNQLIDHMDDDVDRVDKRLNLARNRLRKVTRKAKQYPRCCIILFLSLLLILVCFI

>*Schizosaccharomyces japonicus*

MSEVLLSIDSVQQMIKDRKRLKTSASPDLDAEIKSKLNEIQLKLKELEKKQNDMENDPQIPEFQLKQKEQQLVRYQQRFSRVEEMYREIIQRKNIDIIAGGSNVQSISSQDEDRSMNQSGNTSAVTSISNTYGATMPDNDSAIELGTFTPNHAGTADSNAAQDIDVEALHNIHTQMLLEQEESLVGLEQSVQNQRTLGLQMNSELDEHNTLLNSLSGSVDDTGNRLRRARGRLQRVTKKAEQYPHCFIIFLLLLVLFLVISV

>*Drosophila melanogaster*

MALVDHDSWDIEYEGCERLRHQLLVYLNQRQQLNPRTSQFVQLTSSIQTGIEQLAKDMKHLKVVLDNAITWETSPEEELQQRRIDWDRLTSQLREIREKFANSSRSNVPAASGSAWQDQDLGPGHSNSSRNTALDVEALKQKKTEMLAQQNEGLEVLSATLSRQRQLATQLGNEVEDQNNILDNLANAMDRVETGVQRETQSIGQVNRRDSTWGYWLVIIALFVAIIVVVFV

>*Homo sapiens*

MAPDPWFSTYDSTCQIAQEIAEKIQQRNQYERKGEKAPKLTVTIRALLQNLKEKIALLKDLLLRAVSTHQITQLEGDQRQNLLDDLVTRERLLLASFKNEGAEPDLIRSSLMSEEAKRGAPNPWLFEEPEETRGLGFDEIRQQQQKIIQEQDAGLDALSSIISRQKQMGQEIGNELDEQNEIIDDLANLVENTDEKLRNETRRVNMVDRKSASCGMIMVILLLLVAIVVVAVWPTN

>*Ratus norvegicus*

MAPDPWFSTYDSTCQIAQEIAEKIQERNQCERRGEKTPKLTLTIRTLLKNLKVKIDLLKDLLLRAVSTRQITQLEGDRRQNLLDDLVTRERLLLASFKNEGSEPDLIRSSLMSEEAKRGTPNPWLCEEPEETRGLGFDEIRQQQQKIIQEQDAGLDALSSIISRQKQMGQEIGNELDEQNEIIDDLANLVENTDEKLRTEARRVTLVDRKSASCGMIMVILLLLVAIVVVAVWPTN

>*Mus musculus*

MAPDPWFSTYDSTCQIAQEIAEKIQERNQCERRGEKTPKLTLTIRTLLKNLKVKIDLLKDLLLRAVSTRQITQLEGDRRQNLLDDLVTRERLLLASFKNEGAEPDLIRSSLMSEEAKRGTPNPWLCEEPEETRGLGFDEIRQQQQKIIQEQDAGLDALSSIISRQKQMGQEIGNELDEQNEIIDDLANLVENTDEKLRTEARRVTLVDRKSTSCGMIMVILLLLVAIVVVAVWPTN

>*Gallirallus okinawae*

MAGGVKRGVTNPWLLEESEETRGLGFDDLRKQQRRIIEEQDAGLDALSSIISRQKQMGQEIGNELDEQNEIIDDLTNLVENTDDKLRNQTRHVKMVDQKSTSCGMLVVIVLLLIAIAVVAVWPTH

>*Pan troglodites*

MAPDPWFSTYDSTCQIAQEIAEKIQQRNQYERKGEKAPKLTVTIRALLQNLKEKIALLKDLLLRAVSTHQITQLEGDRRQNLLDDLVTRERLLLASFKNEGAEPDLIRSSLMSEEAKRGAPNPWLFEEPEETRGLGFDEIRQQQQKIIQEQDAGLDALSSIISRQKQMGQEIGNELDEQNEIIDDLANLVENTDEKLRNETRRVNMVDRKSASCGMIMVILLLLVAIVVVAVWPTN

>*Bos taurus*

MAPDPWFSTYDSTCQIAQEIAEKIQQRNQYERNGENTTKLTVTIRALLQKLKEKIALLKDLLLRAVATHQITQLEGDRRQNLLDDLVTRERLLLASFKNEGAEPDLIRSSLMTGGAKRGAPNPWLLEEPEETRGLGFDEIRQQQQKIIQEQDAGLDALSSIISRQKQMGQEIGNELDEQNEIIDDLANLVENTDEKLRTETRRVNLVDRKSTSCGMIMVILLLLVAIVVVAVWPTK

>*Danio rerio*

MSKDLWLENYDAACRLAQEIAENIHERNRQQRTGGNPAKINMTLRASLQKLKQNIAQLRETLNRAAVQRHIMQAEADRRQSLVDDLASRETRLNASFKGDITEAEPSRSTLMAGGNGSGSAVNPWLINESEETKGLSFGEIKNQQQQIIEAQDAGLDALASVLSRQKQMGQEIGNELDEQNEIIDDLAQLVDKTDGRIKNETKRVKLLDSKSASCGMMVVIVLLLIAIIVVACWQ

>*Epinephelus coioides*

MSQDPWLQNYDATCRLAQEIAENIHERNRQQRTGGNPAKINMTLRASLQKLKQNIAQLKEGLLRASSSRRIMQSEADRRQNLIDDLLTREKQLNATFKGDITEPEPSRSTLMGAGAGTSGGVAANPWLVNESEETRGLTFGEIKQQQQRIIEAQDAGLDALSAVISRQKIMGQEIGNELDEQNEIIDDLAHLVDKTDDRIRNETRRVKLVETKSASCGMLVVIVLLLIAIIVVAVWPV

>*Xenopus tropicalis*

MQNLSGKIGQLKESLLRSVSTRQITQLEGDRRQNLVDELLTKERQLQTSFQREGAEPDLVRSSLMAGGARSSSRNNPWVLEEPEETRGFTFQEIKQQQHQIIREQDAGLDALSSILARQKQMGQDIGNELDEQNEIIDDVSALVDTTDSKIRNQTRHIKLVDGKSGSCAMMVVIVLLLVAIVVVAVWPTH

>*Aphyosemion striatum*

MSHDPWLQNYDATCRLAQEIAENIHERNRQQRTGGNPAKINMTLRASLQKLKQNISQLREGLLRISSSRRIMQPEADRRQNLIDELLTREKKLNATFKGDITEPESARSTLMSGGPAASGGSVANPWLVNESEETKGLTFGEIKQHQQRIIQAQDAGLDALAAVISRQKIMGQDIGNELEEHNEIIDDLAQLVDTTDSRIRNETRRVKLVETKSTSCGMLVVIILLLIAIIVIGVWPV

>*Nothobranchius furzeri*

MSQDPWLQNYDATCRLAQEVAENIHERNRQQRTGGNPAKINMTLRASLQKLKQNISQLREGLLRISSSRRIMQSEADRRQNLIDDLLTREKQLNATFKGDATEPEPTRSTLMSGGTAASGGSANPWLVNESEETKGLTFGEIKQQHQRIIQAQDAGLDALAAVISRQKIMGQDIGNELDEHNEIIDDLAQLVDTTDSRIRNETRRVKLVETKSASCGMLVVIILLLIAIVVIGVWPM

>*Arabidopsis thaliana*

MSSAQDPFYIVKEEIQDSIDKLQSTFHKWERISPDMGDQAHVAKELVATCGSIEWQVDELEKAITVAAKDPSWYGIDEAELEKRRRWTSNARTQVRNVKSGVLAGKVSSGAGHASEVRRELMRMPNSGEASRYDQYGGRDDDGFVQSESDRQMLLIKQQDEELDELSKSVQRIGGVGLTIHDELVAQERIIDELDTEMDSTKNRLEFVQKKVGMVMKKAGAKGQMMMICFLLVLFIILFVLVFLT

>*Metschnikowia*

MATSTKSIQAQLAKAAVYIEQLDELLEERERLVSVLHLTPSNADNLDIINLLAKTKAGLEYAQGDLAGGISKELSQELLQTANSYNEKISQLANDPYINVDEYQFHALETSENELNGPKKSVRFKDFDAEESADDSTQMRNQLMGTQGQFRPYTDDFEETEDRNTLLSVDTSNEELFALHQQQMVQQDAHLDALHASIRTQHSMGVNIHDELDEHLILLNDLELGVDGSHTRVRRATRGIALFRRKVRENGSLATIVVLTVILILLLVVLN

>*Saccharomyces cerevisiae*

MDVLKLGYELDQLSDLVEERTRLVSVLKLAPTSNDNVTLKRQLGSILELLQKCAPNDELISRYNTILDKIPDTAVDKELYRFQQQVARNTDEVSKESLKKVRFKNDDELTVMYKDDDEQDEESPLPSTHTPYKDEPLQSQLQSQSQPQPPQPMVSNQELFINQQQQLLEQDSHLGALSQSIGRTHDISLDLNNEIVSQNDSLLVDLENLIDNNGRNLNRASRSMHGFNNSRFKDNGNCVIILVLIVVLLLLLLVL

>*Kluyveromyces marxianus*

MNILKIEYEIDTLLEIVDERIRLIDVLHMQPSKNDNMKLKKHLNQCIDLLKEYENDVMSSEQEKFEELVSKYNEAVESLPDGVVDRMIYHFELKPHFKEQKKVRFKEQLLEEYEQHEPSKSFKPYKDHEEDTDDSLDAKKQELLGNQEAGTPQLSISKHVSNHDIFIQQQQQLMEQETHLSGLSDSISRTHGISLEINQEVGHQNEGLLTDLERQVDRSEGNLQRAGRRLDAYRANSREKNTCFIIVILTVILFILLII

>*Aspergillus luchuensis*

MESWVAGGETVEAQPCQGPDHGFQITSNGYGGNQGGYGQYNPYGQQDANPYSDANAMEQGNGSYEMGSYNQPADATTLLNKCREINDGIADLRAKREGQLAAAQNALLDSSTGKEDQVARQTLDYIEDEVNNGFRYLRDLLKKVKQTPGSGDSRVQTQIDVTSRNLRREIEQYQRCQSDFQKRLREQVRRRYEIANPEASPEEIEQGVDNVLLGQEQSFQVTGSRTRQANDARQAALERSAAIRKIEQDMMELGRLYQEVAELVHQQEPAVEQINQDADNVAQNVSNANNQITEAIASARRARKWKWYALLVVILIIAIVVGVAVGVTEANKSSK

>*Aspergillus clavatus*

MTNPSQLLLLADHIKLSLLERQRAISLDPEPNSQDGEISRSLESLREGIESVEAEVARLEDVHDDGAADLRDQLGHLQSQYQDLSAQFRGQDDFLDSEFANVKNSSPDLKQPVPQHPSSKSVRFMDSATEEADLNRRNLFQPYRDSPSPPGLDATSMDNEQIYDHHANVLREQDEQLDRLGESIGRQHQLSIQIGDELEGHVALLDGMDGDVERHQRRLDGARRRLDKIRKSAGENWSMMTIIGLIIILVILIVILK

>*Aspergillus fumigatus*

MDGNRVTTSDCHYYCGAQMMRKWQINPSPAEKASSSPSIIVNHYPRPSTTAGCPGLIFQASHGRYTCHIPRPNRGHNCCFKMPNPSQLFLLADHIKLSLLERQRAISLNLEPNSQDGEISRSLESLREGIESVESEVSRLGEANDVAAIDLKDQLHYLQSQYRDLSSQFHEQDDSVLDSDFSNVKKSSPDLKQPIPQHPPSKSVRFMDSAAEDADENRRNLFQPYRDSPSPPSPDHSNMSNQQIYDHHAETLREQDEQLDRLGESIGRQHQLSIQIGDELEGHIALLDGMDGDVDRHQRRLDGARKRLDKIRKRAGENWSMMTIIGLIIILVILIVILK

>*Aspergillus oryzae*

MPNPSQLFLLADHIKLSLLERQRAISLDLEPNSQDGEISRSLESLHDGIEDVERDLSQLEQTNDDGAAELKDQLFHLKSQYQDLSSQFSGHSTSAGASGSSPSPEFANVKSSPDLKQPVPQHPPSKSVRFMNSATEEADLERQNLFQPYRDSPSPTGVDQSDLSNEQIYDRHNEIMRDQDEQLDRLGESIGRQHQLSIQIGDELEGHVALLDGMDGDIDRHQHRLDGARKRLDKIRRSAGDNWSLMTIVGLIIILVILIVILK

>*Aspergillus flavus*

MPNPSQLFLLADHIKLSLLERQRAISLDLEPNSQDGEISRSLESLHDGIEDVERDLSQLEQTNDDGAAELKDQLFHLKSQYQDLSSQFSGHSTSAGASGSSPSPEFANVKSSPDLKQPVPQHPPSKSVRFMNSATEEADLERQNLFQPYRDSPSPTGVDQSDLSNEQIYDRHNEIMRDQDEQLDRLGESIGRQHQLSIQIGDELEGHVALLDGMDGDIDRHQHRLDGARKRLDKIRRSAGDNWSLMTIVGLIIILVILIVILK

>*Aspergillus fumigatus*

MPNPSQLFLLADHIKLSLLERERAISLSLEPNSQDGEISRSLESLREGIEGVEANVKRLEESNDEEAADIKDQLMHLRSQYSDLSSQFRGPTSSSAEGESNDRSDSASPQFTNVKGRSPDLKQPVPQHPSSKSVRFMDDSVAAEEDLNRRTLFQPYRDSPSPEGVDTSDMSNQHIYDHHERVMREQDEQLDRLGESIGRQHQLSIQIGDELDGHVALLDEMDGTVDRHQSRLDNARRRIDKIRRSAGENWSMMTIIGLIIILVILIVLLK

>*Botryotinia fuckeliana*

MSTNPNQYFLLADHIKLSLLERQRAISLNLEPTSQDAHISRSLESLREGLENITNERIRLEEAGETSQSLTLQETEHTLQTQYDDLASQFLGHPTASTSSTLSHPNDPSLSNDFTRAAERPRVGSSSSFLKKSLRNSAVAPSPKSVRFSDSPSIQEASDPARAALFPYRDDPSGPPDQSHLDNQQIHAYHSQVLAEQDEALDRLGESIGRQRELSIQIGDELDEHVQMLDEVDRHVDRHQSRLDKARKNLGTVARKAKDNMQMTIILILIIILVLLIIILKBOTRYOTINIAFUCKELIANA

>*Peniclillium digitatum*

MSSPSQLFLLADHIKLSLLERERAISLDLEPNSQDGEISWSLESLRESIEGLVAEQTRLANSHDSAGAAALKDQLTPLQAQYKELSTQFYGAGGEPSDNNMTQASKPATPDLKQPIPQHPPSKSVRFMDSSAAAAVQDEIDEENERNRSNLFRPYRDEPSPRPDQSNMDNQQIYDHHAQTMRDQDDQLDRLGESIGRQHQLSIQIGDELDGHVQLLDGMDGDVERHQTRLDGARRRIDRIRRKTGDNWSMMTIVGLIIILVILIVILK

>*Coccidioides posadasii*

MSNPGQLFLLADHVKLSLLERQRAISLSLEPNSQDGEISRSLESLRDGIESVEKEARRLEEDGDSSFVDLKEEASNLRQQLHDLESQFYENPSSSSKDTISSPNDPSLAQDFVKASSASASTTLKHPTPQHPVSKSVRFTDTLTAQAEEEDPNRRELLQPYRDSPSPSLDHSQLSNEEIHAHHTQIMQEQDDQLDRLGESIGRQHQLSIQIGDELEGQVALLDEVDGHVDRHIGRLDGARRRLGKFKRNARESRGIMWIIGLIILLVILIVILK

>*Aureobasidium pullulans*

MSTSPHQLFLLADHIKLSLLERQRALSLKLPGNNTNEGPITRSLESLRSGIETLSAQAEEDGGDVSELQRLKKQYEELNMQFQGYTSTTATTTPNDSALAPDFAAAKATPTAQRSKSVRFDKPYRDSSPPDADRGKLFNNTTGGQGPGQEEAYTDDPSPPDHTDLSNEQIHAYHKNVLGEQDAQLDTLSQSIGRQRMLGIQMGDEMDDQNAILDDVERGVDRFQGNLDRARGRLGKVARKAKDNWSWVTIGVLILILVLLLVILN

>*Zygosaccharomyces parabailii*

MAMEALQLSYELDKLGDIVEERKRLVTVLKIEPSANDNIKLKKQLSKTLDLLTESKSFDTVGVQEFESRYSELLNDISDEALDLELYQLPKLDKSKKRISKVDDSNTIRGTSPSRDLTKKVRFKDEDLVSYEDQVQQFEPYTDNPEGLSALQDSEEETQRLFNSPAADGGTATIISPQVSNQELFIQQQQQILEQDSHLERLSSSIRTTHNISLSINDEVTDQNDQVLNDLENLLDNGGRNLDRAKRRLMSFERSAKENAPCFIIFVLILILILLLAVL

> *Trichophyton violaceum*

MSNPSQLFLLADHIKLSLLERQRAISLDLEPNAQDGEISRSLESLREGLESAEREAQRLVSAGDASSNDFKEQTSSLRTQYEDLSRQFHGDNAPSLSTQPNYEDLAQDFIRASSTPSILKHPTPQHPVSKSVRFTDTLTASAEEHDPNRDALLRPYSDSPQPSFDPSEATNQEIHDYHSQVMQEQDDHLDRLGESIGRQHQLSIQIGNELEGQVALLDDMDGHVERHQGRLDGARRSLGRFREKSKGSKGMMTIIGLIIVLVILIVILK

**Input sequences: other SNARE genes**

>SpPep12

MSFVDLEQGRHKIEQNGDFPALASSIAQEIHALRGNTAAIHRYLVNNLTKNLHEVLEQSRELSQKVRSDLVRLANIKDTKYGEEASSFALSKLTRDFNTVLAELQRVQQKCAQQESDSVAAAQAALNQDVGQHFIEEEERNVSLSNNSSGQRQPLTESKISNSQLEYQQRLINERQGEIENLTQGINELNEIFRDLSTIINEQGELVTNIEYNVGNTSTNTKNASRQLQIANEHSRKARKRSFCFLVILVVILGVILTALIMG

>SpVti1

METYEQEYRLLRADIEEKLNDLSKSGENSVIQSCQRLLNEIDEVIGQMEIEITGIPTSERGLVNGRIRSYRSTLEEWRRHLKEEIGKSDRKALFGNRDETSGDYIASDQDYDQRTRLLQGTNRLEQSSQRLLESQRIANETEGIGASILRDLHGQRNQLEHSLEMLGDTSGHLDRSLRTLKTMARRLAMNRFFTTAIIAILVILILLVLYSKFR

>SpYkt6

MKLYSVSILRFDPKPVQLLCTASDLSSFSFFQRSSIGEFMNFFTKTVAERTNPGQRQDVEQSNYVFHVYNRSDGLCGVIASDKEYPLRVAYTLLNKILDEFLTKNPRTKWESGAVTLSFPELDTYLSKYQDPKQADTIMRVQQELDETKDVLHKTIESVLARGEKLDDLIQRSDNLSTQSRMFYKSAKKQNSCCIIA

>SpGos1

MKSMLLRDSVKKASQFQRSLHSDPNQAKILLEERRKLLEEANSSADENDSHSMATIKSHFERLKRDEQLLNGVLKKYDAKQEVLSPEELRDAQNFLEMQEANSLDNSIRGTNELLERAYATREDFDYQNSVLGNVTNRINGAAMSIPFINQILRKTSIRRRRDSIILALLISVLMLLFLFFH

>ScPep12.

MSEDEFFGGDNEAVWNGSRFSDSPEFQTLKEEVAAELFEINGQISTLQQFTATLKSFIDRGDVSAKVVERINKRSVAKIEEIGGLIKKVNTSVKKMDAIEEASLDKTQIIAREKLVRDVSYSFQEFQGIQRQFTQVMKQVNERAKESLEASEMANDAALLDEEQRQNSSKSTRIPGSQIVIERDPINNEEFAYQQNLIEQRDQEISNIERGITELNEVFKDLGSVVQQQGVLVDNIEANIYTTSDNTQLASDELRKAMRYQKRTSRWRVYLLIVLLVMLLFIFLIMKL
