# Supplementary material for: Analysis of the SNARE Stx8 recycling reveals that the retromer-sorting motif has undergone evolutionary divergence
Source: PLoS Genet. 2021 Mar 31;17(3):e1009463. doi: 10.1371/journal.pgen.1009463 (PMC8041195; doi:10.1371/journal.pgen.1009463)
Supplement: S5 File — The indicated sequences were used to screen for the presence of potential Snx3-retromer sorting motifs using ScanProsite. (DOCX) [file pgen.1009463.s013.docx]

**S5 File. PC-2 (Pkd2) sequences used to screen for putative motifs recognized by the Snx3-retromer using ScanProsite** (<https://prosite.expasy.org/scanprosite/>).  **Related to Fig. 9**

**Input sequences: Mammal**

>*Homo sapiens*

MVNSSRVQPQQPGDAKRPPAPRAPDPGRLMAGCAAVGASLAAPGGLCEQRGLEIEMQRIRQAAARDPPAGAAASPSPPLSSCSRQAWSRDNPGFEAEEEEEEVEGEEGGMVVEMDVEWRPGSRRSAASSAVSSVGARSRGLGGYHGAGHPSGRRRRREDQGPPCPSPVGGGDPLHRHLPLEGQPPRVAWAERLVRGLRGLWGTRLMEESSTNREKYLKSVLRELVTYLLFLIVLCILTYGMMSSNVYYYTRMMSQLFLDTPVSKTEKTNFKTLSSMEDFWKFTEGSLLDGLYWKMQPSNQTEADNRSFIFYENLLLGVPRIRQLRVRNGSCSIPQDLRDEIKECYDVYSVSSEDRAPFGPRNGTAWIYTSEKDLNGSSHWGIIATYSGAGYYLDLSRTREETAAQVASLKKNVWLDRGTRATFIDFSVYNANINLFCVVRLLVEFPATGGVIPSWQFQPLKLIRYVTTFDFFLAACEIIFCFFIFYYVVEEILEIRIHKLHYFRSFWNCLDVVIVVLSVVAIGINIYRTSNVEVLLQFLEDQNTFPNFEHLAYWQIQFNNIAAVTVFFVWIKLFKFINFNRTMSQLSTTMSRCAKDLFGFAIMFFIIFLAYAQLAYLVFGTQVDDFSTFQECIFTQFRIILGDINFAEIEEANRVLGPIYFTTFVFFMFFILLNMFLAIINDTYSEVKSDLAQQKAEMELSDLIRKGYHKALVKLKLKKNTVDDISESLRQGGGKLNFDELRQDLKGKGHTDAEIEAIFTKYDQDGDQELTEHEHQQMRDDLEKEREDLDLDHSSLPRPMSSRSFPRSLDDSEEDDDEDSGHSSRRRGSISSGVSYEEFQVLVRRVDRMEHSIGSIVSKIDAVIVKLEIMERAKLKRREVLGRLLDGVAEDERLGRDSEIHREQMERLVREELERWESDDAASQISHGLGTPVGLNGQPRPRSSRPSSSQSTEGMEGAGGNGSSNVHV

>*Gorilla gorilla gorilla* OX=9595 GN=PKD2 PE=3 SV=2

MVNSSRVQPQQPGDAKRPPAPRAPDPGRLMAGCAAVGASLASPGGLREQRGLEIEMQRIRQAAARDPPAGAAASPSPPLSSCSRQAWSRDNPGFEAEEEEEEVEGEEGGMVVEMDVEWRPGSRRSAASSAVSSVGARSRGLGGYHGAGHPSGRRRRREDQGPPCPSPVGGGDPLHRHLPLEGQPPRVAWAERLVRGLRGLWGTRLMEESSTNREKYLKSVLRELVTYLLFLIVLCILTYGMMSSNVYYYTRMMSQLFLDTPVSKTEKTNFKTLSSMEDFWKFTEGSLLDGLYWKMQPSNQTEADNRSFIFYENLLLGVPRIRQLRVRNGSCSIPQDLRDEIKECYDVYSVSSEDRAPFGPRNGTAWIYTSEKDLNGSSHWGIIATYSGAGYYLDLSRTREETAAQVASLKKNVWLDRGTRATFIDFSVYNANINLFCVVRLLVEFPATGGVIPSWQFQPLKLIRYVTTFDFFLAACEIIFCFFILYYVVEEILEIRIHKLHYFRSFWNCLDVVIVVLSVVAIGINIYRTSNVEVLLQFLEDQNTFPNFEHLAYWQIQFNNIAAVTVFFVWIKLFKFINFNRTMSQLSTTMSRCAKDLFGFAIMFFIIFLAYAQLAYLVFGTQVDDFSTFQECIFTQFRIILGDINFAEIEEANRVLGPIYFTTFVFFMFFILLNMFLAIINDTYSEVKSDLAQQKAEMELSDLIRKGYHKALVKLKLKKNTVDDISESLRQGGGKLNFDELRQDLKGKGHTDAEIEAIFTKYDQDGDQELTEHEHQQMRDDLEKEREDLDLDHSSLPRPMSSRSFPRSLDDSEEDDDEDSGHSSRRRGSISSGVSYEEFQVLVRRVDRMEHSIGSIVSKIDAVIVKLEIMERAKLKRREVLGRLLDGVAEDERLGRDSEIHREQMERLVREELERWESDDAASQISHGLGTPVGLNGQPRPRSSRPSSSQSTEGMEGAGGNGSSNVHV

>*Pan troglodytes* OX=9598 GN=PKD2 PE=2 SV=1

MVNSSRVQPQQPGDAKRPPAPRAPDPGRLMAGCAAVGASLAAPGGLREQRGLEIEMQRIRQAAARDAPAGAAASPSPPLSSCSRQAWSRDNPGFEAEEEEEEVEGEEGGMVVEMDVEWRPGSRRSAASSAVSSVGARSRGLGGYHGAGHPSGRRRRREDQGPPCPSPVGGGDPLHRHLPLEGQPPRVAWAERLVRGLRGLWGTRLMEESSTNREKYLKSVLRELVTYLLFLIVLCILTYGMMSSNVYYYTRMMSQLFLDTPVSKTEKTNFKTLSSMEDFWKFTEGSLLDGLYWKMQPSNQTEADNRSFIFYENLLLGVPRIRQLRVRNGSCSIPQDLRDEIKECYDVYSVSSEDRAPFGPRNGTAWIYTSEKDLNGSSHWGIIATYSGAGYYLDLSRTREETAAQVASLKKNVWLDRGTRATFIDFSVYNANINLFCVVRLLVEFPATGGVIPSWQFQPLKLIRYVTTFDFFLAACEIIFCFFIFYYVVEEILEIRIHKLHYFRSFWNCLDVVIIVLSVVAIGINIYRTSNVEVLLQFLEDQNTFPNFEHLAYWQIQFNNIAAVTVFFVWIKLFKFINFNRTMSQLSTTMSRCAKDLFGFAIMFFIIFLAYAQLAYLVFGTQVDDFSTFQECIFTQFRIILGDINFAEIEEANRVLGPIYFTTFVFFMFFILLNMFLAIINDTYSEVKSDLAQQKAEMELSDLIRKGYHKALVKLKLKKNTVDDISESLRQGGGKLNFDELRQDLKGKGHTDAEIEAIFTKYDQDGDQELTEHEHQQMRDDLEKEREDLDLDHSSLPRPMSSRSFPRSLDDSEEDDDEDSGHSSRRRGSISSGVSYEEFQVLVRRVDRMEHSIGSIVSKIDAVIVKLEIMERAKLKRREVLGRLLDGVAEDERLGRDSEIHREQMERLVREELERWESDDAASQISHGLGTPVGLNGQPRPRSSRPSSSQSTEGMEGAGGNGSSNVHV

>*Pongo abelii* OX=9601 GN=PKD2 PE=3 SV=1

MVNSSRVQPQQPGDAKRPPAPRAPDPGRLMAGCTAVGASLAAPGGLREQRGLEIEMQRIRQAAARDPPAGASASPSPPLSSCSRQAWSRDNPGFEAEEEEEEVEGEEGGMVVEMDVEWRPGSRRSAASSAVSSAGARGRGLGGYHGAGHPSGRRRRREDQGPPCPSPVGGGDPLHRHLPLDGQPPRVAWAERLGTGLLGLWGTRLMEESSTNREKYLKSVLRELVTYLLFLIVLCILTYGMMSSNVYYYTRMMSQLFLDTPVSKTEKTNFKTLSSMEDFWKFTEGSLLDGLYWKMQPSNQTEADNRSFIFYENLLLGVPRIRQLRVRNGSCSIPQDLRDEIKECYDVYSVSSEDRAPFGPRNGTAWIYTSEKDLNGSSHWGIIATYSGAGYYLDLSRTREETAAQVASLKKNVWLDRGTRATFIDFSVYNANINLFCVVRLLVEFPATGGVIPSWQFQPLKLIRYVTTFDFFLAACEIIFCFFILYYVVEEILEIRIHKLHYFRSFWNCLDVVIVVLSVVAIGIHIYRTSNVEVLLQFLEDQNTFPNFEHLAYWQIQFNNIAAVTVFFVWIKLFKFINFNRTMSQLSTTMSRCAKDLFGFAIMFFIIFLAYAQLAYLVFGTQVDDFSTFQECIFTQFRIILGDINFAEIEEANRVLGPIYFTTFVFFMFFILLNMFLAIINDTYSEVKSDLAQQKAEMELSDLIRKGYHKALVKLKLKKNTVDDISESLRQGGGKLNFDELRQDLKGKGHTDAEIEAIFTKYDQDGDQELTEHEHQQMRDDLEKEREDLDLDHSSLPRPMSSRSFPRSLDDSEEDDDEDSGHSSRRRGSISSGVSYEEFQVLVRRVDRMEHSIGSIVSKIDAVIVKLEIMERAKLKRREVLGRLLDGVAEDERLGRDSEIHREQMERLVREELERWESDDAASQISHGLGTPVGLNGQPRPRSSRPSSSQSTEGMEGAGGNGSSNVHV

>*Papio anubis* OX=9555 GN=PKD2 PE=3 SV=1

MVNSSRVQPQQPGDAKRPPAPRAPDPGRLMAGCAAVGASLAAPGGLRDQRGLEIEMQRIRQAAARDPPAGASASPSPPLSSCSRQAWSRDNPGFEAEEEEEEEVEGEEGGMVVEMDVEWRPGSRRSAASSAVSSAGARGRGLGGYHSAGHPSGRRRRREDQGPPCPSPAGGGDPLHRHLPLDGQAPRVAWAERLVRGLRGLWGTRLMEESSTNREKYLKSVLRELVTYLLFLIVLCILTYGMMSSNVYYYTRMMSQLFLDTPVSKTEKTNFKTLSSMEDFWKFTEGSLLDGLYWKMQPSNQTEADNRSFIFYENLLLGVPRIRQLRVRNGSCSIPQDLRDEIKECYDVYSVSSEDRAPFGPRNGTAWIYTSEKDLNGSSHWGIIATYSGAGYYLDLSRTREETAAQVASLKKNVWLDRGTRATFIDFSVYNANINLFCVVRLLVEFPATGGVIPSWQFQPLKLIRYVTTFDFFLAACEIIFCFFILYYVVEEILEIRIHKLHYFRSFWNCLDVVIVVLSVVAIGISIYRTSNVEVLLQFLEDQNTFPNFEHLAYWQIQFNNIAAVTVFFVWIKLFKFINFNRTMSQLSTTMSRCAKDLFGFAIMFFIIFLAYAQLAYLVFGTQVDDFSTFQECIFTQFRIILGDINFAEIEEANRVLGPIYFTTFVFFMFFILLNMFLAIINDTYSEVKSDLAQQKAEMELSDLIRKGYHRALVKLKLKKNTVDDISESLRQGGGKLNFDELRQDLKGKGHTDAEIEAIFTKYDQDGDQELTEHEHQQMRDDLEKEREDLDLDHSSLPRPMSSRSFPRSLDDSEEDDDEDSGHSSRRRGSISSGVSYEEFQVLVRRVDRMEHSIGSIVSKIDAVIVKLEIMERAKLKRREVLGRLLDGVAEDERLGRDSEIHREQMERLVREELERWESDDAASQISHGLGTPVGLNGQPRPRSSRPSSSQSTEGMEGAGGNGSSNVHV

>*Macaca nemestrina* OX=9545 GN=PKD2 PE=3 SV=1

MVNSSRVQPQQPGDAKRPPAPRAPDPGRLMAGCAAVGASLAAPGGLRDQRGLEIEMQRIRQAAARDPPAGASASPSPPLSSCSRQAWSRDNPGFEAEEEEEEEVEGEEGGMVVEMDVEWRPGSRRSAASSAVSSAGARGRGLGGYHSAGHPSGRRRRREDQGPPCPSPAGGGDPLHRHLPLDGQAPRVAWAERLVRGLRGLWGTRLMEESSTNREKYLKSVLRELVTYLLFLIVLCILTYGMMSSNVYYYTRMMSQLFLDTPVSKTEKTNFKTLSSMEDFWKFTEGSLLDGLYWKMQPSNQTEADNRSFIFYENLLLGVPRIRQLRVRNGSCSIPQDLRDEIKECYDVYSVSSEDRAPFGPRNGTAWIYTSEKDLNGSSHWGIIATYSGAGYYLDLSRTREETAAQVASLKKNVWLDRGTRATFIDFSVYNANINLFCVVRLLVEFPATGGVIPSWQFQPLKLIRYVTTFDFFLAACEIIFCFFILYYVVEEILEIRIHKLHYFRSFWNCLDVVIVVLSVVAIGISIYRTSNVEVLLQFLEDQNTFPNFEHLAYWQIQFNNIAAVTVFFVWIKLFKFINFNRTMSQLSTTMSRCAKDLFGFAIMFFIIFLAYAQLAYLVFGTQVDDFSTFQECIFTQFRIILGDINFAEIEEANRVLGPIYFTTFVFFMFFILLNMFLAIINDTYSEVKSDLAQQKAEMELSDLIRKGYHKALVKLKLKKNTVDDISESLRQGGGKLNFDELRQDLKGKGHTDAEIEAIFTKYDQDGDQELTEHEHQQMRDDLEKEREDLDLDHSSLPRPMSSRSFPRSLDDSEEDDDEDSGHSSRRRGSISSGVSYEEFQVLVRRVDRMEHSIGSIVSKIDAVIVKLEIMERAKLKRREVLGRLLDGVAEDERLGRDSEIHREQMERLVREELERWESDDAASQISHGLGTPVGLNGQPRPRSSHPSSSQSTEGMEGAGGNGSSNVHV

>*Cercocebus atys* OX=9531 GN=PKD2 PE=3 SV=1

MVNSSRVQPQQPGDAKRPPAPRAPDPGRLMAGCAAVGTSLAAPGGLRDQRGLEIEMQRIRQAAARDPPAGASASPSPPLSSCSRQAWSRDNPGFEAEEEEEEEVEGEEGGMVVEMDVEWRPGSRRSAASSAVSSAGARGRGLGGYHSAGHPSGRRRRREDQGPPCPSPAGGGDPLHRHLPLDGQAPRVAWAERLVRGLRGLWGTRLMEESSTNREKYLKSVLRELVTYLLFLIVLCILTYGMMSSNVYYYTRMMSQLFLDTPVSKTEKTNFKTLSSMEDFWKFTEGSLLDGLYWKMQPSNQTEADNRSFIFYENLLLGVPRIRQLRVRNGSCSIPQDLRDEIKECYDVYSVSSEDRAPFGPRNGTAWIYTSEKDLNGSSHWGIIATYSGAGYYLDLSRTREETAAQVASLKKNVWLDRGTRATFIDFSVYNANINLFCVVRLLVEFPATGGVIPSWQFQPLKLIRYVTTFDFFLAACEIIFCFFILYYVVEEILEIRIHKLHYFRSFWNCLDVVIVVLSVVAIGISIYRTSNVEVLLQFLEDQNTFPNFEHLAYWQIQFNNIAAVTVFFVWIKLFKFINFNRTMSQLSTTMSRCAKDLFGFAIMFFIIFLAYAQLAYLVFGTQVDDFSTFQECIFTQFRIILGDINFAEIEEANRVLGPIYFTTFVFFMFFILLNMFLAIINDTYSEVKSDLAQQKAEMELSDLIRKGYHKALVKLKLKKNTVDDISESLRQGGGKLNFDELRQDLKGKGHTDAEIEAIFTKYDQDGDQELTEHEHQQMRDDLEKEREDLDLDHSSLPRPMSSRSFPRSLDDSEEDDDEDSGHSSRRRGSISSGVSYEEFQVLVRRVDRMEHSIGSIVSKIDAVIVKLEIMERAKLKRREVLGRLLDGVAEDERLGRDSEIHREQMERLVREELERWESDDAASQISHGLGTPVGLNGQSRPRSSRPSSSQSTEGMEGAGGNGSSNVHV

>*Callithrix jacchus* OX=9483 GN=PKD2 PE=2 SV=2

MVNSRRVQPQQPGDAKQSPAPRAPGPGRLMAGGAAVGASLAARGGLREQRGLEIEMQRIRQAAARDPPAGASASPSPPLSSCSRQAWSRDNPGFEAEEEEEEEEEEEVEGEEGGMVVEMDVEWRPGSRRSAASSAVSSAGARSRGLGGYHGAGHPSGRRHRREDQGPPCPSPTGGGDPLHRHLPLDGQPPRVAWAVRLVRGLRGLWGTRLMEESSTNREKYLKSVLRELVTYLLFLIVLCILTYGMMSSNVYYYTRMMSQLFLDTPVSKTEKTNFKTLSSMEDFWKFTEGSLLDGLYWKMQPSNQTEADNRSFIFYENLLLGVPRIRQLRVRNGSCSIPQDLRDEIKECYDVYSVSSEDRAPFGPRNGTAWIYTSEKDLNGSSHWGIIATYSGAGYYLDLSRTREETAAQVASLKKNVWLDRGTRATFIDFSVYNANINLFCVVRLLVEFPATGGVIPSWQFQPLKLIRYVTTFDFFLAACEIIFCFFILYYLVEEILEIRIHKLHYFRSFWNCLDVLIIVLSVVAIGINIYRTSNVDVLLQFLEDQNTFPNFEHLAYWQIQFNNVAAVIVFFVWIKLFKFINFNRTMSQLSTTMSRCAKDLFGFAIMFFIIFLAYAQLAYLVFGTQVDDFSTFQECIFTQFRIILGDINFAEIEEANRVLGPIYFTTFVFFMFFILLNMFLAIINDTYSEVKSDLAQQKAEMELSDLIRKGYHKALVKLKLKKNTADDISESLRQGGGKLNFDELRQDLKGKGHTDAEIEAIFTKYDQDGDQELTEHEHQQMRDDLEKEREDLDLDHSSLPRPMSSRSFPRSLDDSEEDDDEDSGHSSRRRGSISSGVSYEEFQVLVRRVDRMEHSIGSIVSKIDAVIVKLEIMERAKLKRREVLGRLLDGVAEDERLGRDGEIHREQMERLVREELERWESDDAASQISHGLGTPVGLNGQPCPRSSRPSSSQSTEGMEGAGGNGNSNVHI

>*Felis catus* OX=9685 GN=PKD2 PE=3 SV=2

MVNSSRVQPQQPGDPKRPPAARAAGPGRLMTGGAAVGAGLAAPGGLREQRGLEIEMERIRQAAARDPPAGASASPSPPLSSCSRQAWSRDNPGFEAEEEEEEEEVEGEEGGMVVEMDVEWRPGSRRSAASSSVSSVGARGRGLGGYHGTGHPSGRRRRREDQGPPSPSPAGGGDPLHRHLPLDGQPPRVAWAERLVRGLRGLWGTRLMEESNTNREKYLKSVLRELATYLLFLIVLCILTYGMMSSSVYYYTRIMSQLFLDTPVSKTEKTNFKTLSSMEDFWKFTEGALLDGLYWKTQPSNNTDADNRSFIYYENLLLGVPRIRQLKVRNGSCSIPQDLRDEIKECYDVYSVSSEDRAPFGPRNGTAWIYTSEEDLNGSSHWGMIATYSGAGYYLDLSRTREETAAQVASLKNNVWLDRGTRATFIDFSVYNANINLFCVIRLLVEFPATGGVIPSWQFQPVKLIRYVTTFDFFLAACEIIFCFFIFYYVVEEILEIRIHKLHYFRSFWNCLDVVIVVLSVVAIGINIYRTSNVEMLLQFLEDQNTFPNFEHLAYWQIQFNNIAAVIVFFVWIKLFKFINFNRTMSQLSTTMSRCAKDLFGFAIMFFIIFLAYAQLAYLVFGTQVDDFSTFQECIFTQFRIILGDINFAEIEEANRVLGPIYFTTFVFFMFFILLNMFLAIINDTYSEVKSDLAQQKAEMELSDLIRKGYHKALVKLKLKKNTVDDISESLRQGGGKLNFDELRQDLKGKGHTDAEIEAIFTKYDQDGDQELTEHEHQQMRDDLEKEREDLDLDHSSLPRPMSSRSFPRSLDDSEEDDDEDSGHSSRRRGSISSGVSYEEFQVLVRRVDRMEHSIGSIVSKIDAVIVKLEIMERAKLKRREVLGRLLDGVAEDERLGRDSEIHREQMERLVREELERWESDDAASQISHGLGTPVGLNGQPRPRSSRPSSSQSADGIEGAGANGSSNLQV

>*Ursus arctos horribilis* OX=116960 GN=PKD2 PE=3 SV=1

MVNSSRVQPQQPGDPKRPPASRAAGPGRLMAGGAAVGAGLAAPGGLREQRGLEIEMERIRQAAARDPPAGASASPSPPLSSCSRQAWSRDNPGFEAEEEEEEEEVEGEEGGMVVEMDVEWRPGSRRSASSSAVSSVGARGRGLGGYHGSGHPSGRRRRREDQGPPSSSPAGGGDPLHRHLPLDGQPPRVAWAERLVRGLRGLWGTRLMEESNTNREKYLKSVLRELATYLLFLIVLCILTYGMMSSSVYYYTRIMSQLFLDTPVSKTEKTNFKTLSSMEDFWKFTEGALLDGLYWKTQPSNRSEADNRSFIYYENLLLGVPRIRQLKVRNGSCSIPQDLRDEIKECYDVYSARSEDRAPFGPRNGTAWIYTSEKDLNGSSHWGMIATYSGAGYYLDLSRTREETAAQVASLKKNGWLDRGTRATFIDFSVYNANINLFCVIRLLVEFPATGGVIPSWQFQPVKLIRSVTTFDFFLAACEIIFCFFILYYVVEEILEIRIHKLHYFRSFWNCLDVVIIVLSVVAIAINIYRTSNVEVLLQFLEDQNTFPNFEHLAYWQIQFNNIAAVIVFFVWIKLFKFINFNRTMSQLSTTMSRCAKDLFGFAIMFFIIFLAYAQLAYLVFGTQVDDFSTFQECIFTQFRIILGDINFAEIEEANRVLGPIYFTTFVFFMFFILLNMFLAIINDTYSEVKSDLAQQKAEMELSDLIRKGYQKALVKLKLKKNTVDDISESLRQGGGKLNFDELRQDLKGKGHTDAEIEAIFTKYDQDGDQELTEHEHQQMRDDLEKEREDLDLDHSSLPRPMSSRSFPRSLDDSEEDDDEDSGHSSRRRGSISSGVSYEEFQVLVRRVDRMEHSIGSIVSKIDAVIVKLEIMERAKLKRREVLGRLLDGVVEDERLGRDGEIHREQMERLVREELERWESDDAASQISHGLGTPVGLNGQPRSRSSRPSSSQSTEGIEGAGANGSSNIHV

>*Capra hircus* OX=9925 GN=PKD2 PE=3 SV=1

MVNSSRVQPQQPGDARRSPAPRAPGPGRLMAGGATAGAGLAAPGGLREQRGLEIEMERIRQAAARDPPAGASASPSPPLSSCSRQAWSRDNPGFEAEEEEEEEEVEGEEGGMVVEMDVEWRPGSRRSASSSAVSSAGARGRGLGGYHSAGHPSGRRRQREDQGPPSPSPAGGGDPLHRHLPLDGQHPRVAWAERLVRGLRGLWGTRLMEESSTDREKYLKSVLRELATYLLFLIVLCILTYGMMSSSVYYYTRIMSQLFLDTPVSKMEKTNFKTLSSMEDFWKFTEGALLDGLYWKTQPSNRTEADNRSFIYYENLLLGVPRIRQLRVRNGSCSIPLDLRDEIKECYDVYSVSSEDRAPFGPRNGTAWIYTSEKDLNGSSHWGMIATYSGAGYYLDLSRTREETAAQVANLKKNVWLDRGTRAIFIDFSVYNANVNLFCVIRLLIEFPATGGVIPSWQFQPVKLIRYVTTFDFFLAACEIIFCFFILYYVVEEILEIRIHKLHYFRSFWNCLDVVIIVLSVVAIGINIYRTSNVEVLLQFLEDQNTFPNFENLAYWQTQFNNIAAVIVFFVWIKLFKFISFNRTMSQLSTTMSRCAKDLFGFAMMFFIIFLAYAQLAYLVFGTQVDDFSTFQECIFTQFRIILGDINFAEIEEANRVLGPIYFTTFVFFMFFILLNMFLAIINDTYSEVKSDLAQQKAEMELSDLIRKGYHKALIKLKLKKNTVDDISESLRQGGGKLNFDELRQDLKGKGHTDAEIEAIFTKYDQDGDQELTEHEHQQMRDDLEKEREDLDLDHSSLPRPMSSRSFPRSLDDSEEEDDDDSGHSSRRRGSISSGVSYEEFQVLVRRVDRMEHSIGSIVSKIDAVIVKLEIMERAKLKRREVLGRLLDGVAEDERLGRDSEIHREQMERLVREELERWESDDAASQISHGLGTPLGLNGQPRPRSSRPSSSQSTEGMEGGGGNGSANIHV

>*Bos taurus* OX=9913 GN=PKD2 PE=2 SV=1

MVNSSRVQPQQPGDARRSPAPRAPGPGRLMAGGAIAGAGLAAPGGLREQRGLEIEMERIRQAAARDPPAGASASPSPPLSSCSRQAWSRDNPGFEAEEEEEEEEVEGEEGGMVVEMDVEWRPGSRRSASSSAVSSAGARGRGLGGYHGAGHPSGRRRQREDQGPPSPSPAGGGDPLHRHLPLDGQHPRVAWAERLVRGLRGLWGTRLMEESSTDREKYLKSVLRELATYLLFLIVLCILTYGMMSSSVYYYTRIMSQLFLDTPVSKMEKTNFKTLSSMEDFWKFTEGALLDGLYWKTQPSNRTEADNRSFIYYENLLLGVPRIRQLRVRNGSCSIPLDLRDEIKECYDVYSVSSEDRAPFGPRNGTAWIYTSEKDLNGSSHWGMIATYSGAGYYLDLSRTREETAAQVANLKKNVWLDRGTRAIFIDFTVYNANINLFCVIRLLIEFPATGGVIPSWQFQPVKLIRYVTTFDFFLAACEIIFCLFILYYVVEEILEIRIHKLHYFRSFWNCLDVVIIVLSVVAIGINIYRTSNVEALLQFLEDQNTFPNFENLAYWQTQFNNIAAVIVFFVWIKLFKFINFNRTMSQLSTTMSRCAKDLFGFAIMFFIIFLAYAQLAYLVFGTQVDDFSTFQECIFTQFRIILGDINFAEIEEANRVLGPIYFTTFVFFMFFILLNMFLAIINDTYSEVKSDLAQQKAEMELSDLIRKGYHKALIKLKLKKNTVDDISESLRQGGGKLNFDELRQDLKGKGHTDAEIEAIFTKYDQDGDQELTEHEHQQMRDDLEKEREDLDLDHSSLPRPMSSRSFPRSLDDSEEEDDDDSGHSSRRRGSISSGVSYEEFQVLVRRVDRMEHSIGSIVSKIDAVIVKLEIMERAKLKRREVLGRLLDGVAEDERLGRDNEIHREQMERLVREELERWESDDAASQISHGLGTPLGLNGQPRPRSSRPSSSQSTEGMEGGGGNGSANIHV

>*Equus caballus* OX=9796 GN=PKD2 PE=3 SV=2

MVHSSRVQPQQPGDAKRPPAPRAAGPGRLMAGGLREQRGLEIEMERIRQAAARDPPAGASASPSPPLSSCSRQAWSRDNPGFEAEEDEEEEEVEGEEGGMVVEMDVEWRPGSRRSAASSAVSSAGARGRGLGGYHGAGHPSGRRHRREDQGPPSPSPAGGGDPLHRHLPLDGQPPRVAWAERLVRGLRGLWGTRLMEESNTNREKYLRSVLRELATYLLFLIVLCILTYGMMSSSVYYYTRIMSQLFLDTPVSKTEKTNFKTLSSMEDFWKFTEGALLDGLYWKAQPSNGTEADNRSFIYYENLLLGVPRIRQLRVRNGSCSIPQDLRDEIKECYDVYSVSSEDRAPFGPRNGTAWIYTSEKDLNGSSHWGIIATYSGAGYYLDLSRTREETAAQVASLKRNVWLDRGTRATFIDFSVYNANVNLFCVIRLLVEFPATGGVVPSWQFQPVKLIRYVTTFDFFLAACEILFCFFILYYVVEEILEIRIHKLHYFRSFWNCLDVVIIVLSVVAIGINIYRTSNMEVLLQFLEDQNTFPNFEHLAYWQIQFNSIAAVIVFFVWIKLFKFINFNRTMSQLSTTMSRCAKDLFGFAIMFFIIFLAYAQLAYLVFGTQVDDFSTFQECIFTQFRIILGDINFAEIEEANRVLGPIYFTTFVFFMFFILLNMFLAIINDTYSEVKSDLAQQKAEMELSDLIRKGYHKALVKLKLKKNTVDDISESLRQGGGKLNFDELRQDLKGKGHTDAEIEAIFTKYDQDGDQELTEHEHQQMRDDLEKEREDLDLDHSSLPRPMSSRSFPRSLDDSEEDDDEDSGHSSRRRGSISSGVSYEEFQVLVRRVDRMEHSIGSIVSKIDAVIVKLEIMERAKLKRREVLGRLLDGVAEDERLGRDNEIHREQMERLVREELERWESDDAASQISHGLGTPVGLNGQPRPRSSRPSSSQSTEGMEGGGGNGSSNLHV

>*Enhydra lutris kenyoni* OX=391180 GN=LOC111156195 PE=3 SV=1

MVNSSRVQPQQPGDPKRPPASRAAGPGRLMAGGAAVGAGLAAPGGLREQRGLEIEMERIRQAAARDPPAGASASPSPPLSSCSRQAWSRDNPGFEAEEEEEEEEVEGEEGGMVVEMDVEWRPGSRRSAASSAVSSVGARGRGLGGYHDPGHPSGRRHRREDQGPPSPSPAGGGDPLHRHLPLDGQPPRVAWAERLVRGLRGLWGTRLMEESSTNREKYLKSVLRELTTYLLFLIVLCILTYGMMSSSAYYYTRIMSQLFLDTPVSKTEKTNFKTLSSMEDFWKFTEGALLDGLYWKTQPSNRTEADNRSFIYYENLLLGVPRIRQLKVRNGSCSIPQDLRDEIKECYDVYSVSSEDRAPFGPRNGTAWIYTSEKDLNGSSHWGMIATYSGAGYYLDLSRTREETAAQVASLKKNGWLDRGTRATFIDFSVYNANINLFCVIRLLVEFPATGGVIPSWQFQPVKLTRYVTTFDFFLAACEIIFCFLILYYVVEEILEIRIHKLHYFRSFWNCLDVVIVVLSMVAIGINIHRASNVEVLLQFLEDQNTFPNFEHPAYWQIQFNNIAAVIVFFVWIKLFKFINFNRTMSQLSTTMSRCAKDLFGFAIMFFIIFLAYAQLAYLVFGTQVDDFSTFQECIFTQFRIILGDINFAEIEEANRVLGPIYFTTFVFFMFFILLNMFLAIINDTYSEVKSDLAQQKAEMELSDLIRKGCHKALVKLKLKKNTVDDISESLRQGGGKLNFDELRQDLKGKGHTDAEIEAIFTKYDQDGDQELTEHEHQQMRDDLEREREDLDLDHSSLPRPMSSRSFPRSLDDSEEDDDEDSGHSSRRRGSISSGVSYEEFQVLVRRVDRMEHSIGSIVSKIDAVIVKLEIMERAKLKRREVLGRLLDGVAEDERLGRDSEVHREQMERLVREELERWESDDAASQISHGLGTPLGPNGPPRPRSSRPSSSQSAEGLEGAGANGSSSIHV

>*Neomonachus schauinslandi* OX=29088 GN=PKD2 PE=3 SV=1

MVNSSRVQPQQPGAPKRSPASRAAGPGRLMAGGAAVGAGLGAPGGLREQRGLEIEMERIRQAAARDPPAGASASPSPPLSSCSRQAWSRDNPGFEAEEEEEEEEVEGEEGGMVVEMDVEWRPGSRRSAASSAVSSVGSRGRGLGGYHGTGHPSGRRRRREDQGPPSPSPAGGGDPLHRHLPLDGQPPRVAWAERLVRGLRGLWGTRLMEESNTNREKYLKSVLRELATYLLFLIVLCILTYGMMSSSVYYYTRIMSQLFLDTPVSKTEKTNFKTLSSMEDFWKFTEGALLDGLYWKTQPSNRTEADNRSFIYYENLLLGVPRIRQLKVRNGSCSIPQDLRDEIKECYDVYSVSSEDRAPFGPRNGTAWIYTSEKDLNGSSHWGMIATYSGAGYYLDLSRTREETAAQVASLKKNGWLDRGTRATFIDFSVYNANINLFCVIRLLVEFPATGGVIPSWQFQPVKLIRYVTTFDFFLAACEIIFCFFILYYVVEEILEIRIHKLHYFRSFWNCLDVVIIVLSVVAIGINIYRTSNVEVLLQFREDQNTFPNFEHLAYWQIQFNNIAAVIVFFVWIKLFKFINFNRTMSQLSTTMSRCAKDLFGFAIMFFIIFLAYAQLAYLVFGTQVDDFSTFQECIFTQFRIILGDINFAEIEEANRVLGPIYFTTFVFFMFFILLNMFLAIINDTYSEVKSDLAQQKAEMELSDLIKKGYHKALVKLKLKKNAVDDISESLRQGGGKLNFDELRQDLKGKGHTDAEIEAIFTKYDQDGDQELTEHEHQQMRDDLEKEREDLDLDHSSLPRPMSSRSFPRSLDDSEEDDDEDSGHSSRRRGSISSGVSYEEFQVLVRRVDRMEHSIGSIVSKIDAVIVKLEIMERAKLKRREVLGRLLDGVAEDERLGRDSEIHREQMERLVREELGRCESDDAASQTSHGLGTPVGLNGQARTRSSRPSSSQSAEGIEGAGANGSSNIHV

>*Sus scrofa* OX=9823 GN=PKD2 PE=2 SV=1

MVNSSRVQPQQPGDAQRPAAPRTAGPGRLMAGGGAAGAGLAAPGGLREQRGLEIEMERIRQAAARDPPAGASASPSPPLSSCSRQAWSRDNPGFEAEEEEEEDEVEGEEGGMVVEMDVEWRPGSRRSAASSAVSSAGARGRGLGSFHGAGHPSGRRRRREDQGPPSPSPAGGGDPLHRHLPLDGQPPRVAWAERLVRGLRGLWGTRLMEESSTDRERYLKSVLRELATYLLFLIVLCVLTYGMMSSSVYYYTRIMSQLFLDTPVSRTEKTNFKTLSSVEDFWKFAEGALLDGLYWKTQPSNGTEAHNRSFIYYENLLLGVPRIRQLRVRNGSCSIPQDLRDEIKECYDVYSVSSEDRAPFGPRNGTAWIYTSEKDLNGSSHWGMLTTYSGAGYYLDLSRTREETAAQLAHLRKHAWLDRGTRATFIDFSVYNANINLFCVIRLLVEFPATGGVIPSWQFQPVKLIQYVTTFDFFLAACEIIFCFFILYYVVEEILEIRIHKLHYFRSFWNCLDVVIIVLSVAAIGINIHRTSNVEMLLQFLEDQNTYPNFENLAYWQIQFNNIAAVIVFFVWIKLFKFISFNRTMSQLSTTMSRCAKDLFGFAIMFFIIFLAYAQLAYLVFGTQVDDFSTFQECIFTQFRIILGDINFAEIEEANRVLGPIYFTTFVFFTFFILLNMFLAIINDTYSEVKSDLAQQKAEMELSDLIRKGYHKALVKLKLKKNTVDDISESLRQGGGKLNFDELRQDLKGKGHTDAEIEAIFTKYDQDGDQELTEREHQQMRDDLEKEREDLDLEHSSLPRPMSSRSFPRSLDDSEEDDDDDSGHSSRRRGSISSGVSYEEFQVLVRRVDRMEHSIGSIVSKIDAVIVKLEIMERNKLKRREVLGRLLDGVAEDERLGRDSEIHREQMERLVREELERWESDDAASQISHGLGTPVGLNGQPRPRSSRPSSSQSTEGMEGGGGNGSADIHV

**Used for the Weblogo**:

H.sapiens EQRGLEIEMQRIRQA

G.gorilla EQRGLEIEMQRIRQA

P.troglodites EQRGLEIEMQRIRQA

P.abelii EQRGLEIEMQRIRQA

C.jacchus EQRGLEIEMQRIRQA

N.schauinslandi EQRGLEIEMERIRQA

S.scrofa EQRGLEIEMERIRQA

E.lutris EQRGLEIEMERIRQA

E.caballus EQRGLEIEMERIRQA

B.taurus EQRGLEIEMERIRQA

C.hircus EQRGLEIEMERIRQA

U.arctos EQRGLEIEMERIRQA

F.catus EQRGLEIEMERIRQA

P.anubis DQRGLEIEMQRIRQA

M.nemestrina DQRGLEIEMQRIRQA

C.atis DQRGLEIEMQRIRQA

:********:*****

**Input sequences: Fish**

>*Danio rerio*

MSSSRVRPQAPQSPAASASASPPPHEGIEMEKMHHEEVGLGVPDETPSSPPTSSSRQAWSRDNPGFEPEEGMMEADWPPESQGRRSVSTTSSSSSGGVPGNFSGISARINRGLYPTPPAQEHRSCGKRILEKMRVLWDTRLLGESNSNREMYLKTVLREMITYILFLLTLCIITYGMVSTNMYYYTKVMSQLFLDTPLSSGEPTNFKSLSTMEDFWKFTEGPFLNGMYWELWYNNKSLPENQSLIYYENLLLGVPRLRQLRVRNESCSVHEDLRDEVYDCYNVYSPANEDKAPFGPKNGTAWRFKDESSLGESSYWGQVSTYGGGGYYQDLSRTREKSANQLQELKNNLWLDRGTRAVFLDFSIYNGNVNLFCIVRLLVEFPATGGAVPSWQFQTVRLLRYVSSWDYFVGMCEVSFCLFVLYYLVEEALEIRLHRLRYFKSLWNCLDVLIVALSVPAIIMNICRTSAVSHRLHFLLENHSTYPNFEPLARLQVHFNNLAAIIVFLSWVKLFKFINFNKTMNQLSTTMSRCAKDLMGFAIMFFIVFLAYAQLAYLVFGTQVNDFSTFQACIFTQFRIILGDFDFSEIEEADSVLGPIYFTTFVFFIFMILLNMFLAIINDTYSEVKADMAQQRSEMEITDLIKKSYNRAMVKLKLKKSSINDIPDSLQQAAGKLSFDELRQDLRGKGHSDAEIEAIFAKYDLDGDQELTEHEHQQMRDDLEKEREDLDLEHSSLPRPASGRSFSRSQDDSEEDDDEDSGHSSRRRGSSSGGVSYEEFQVLVRRVDRMEHSIGSIVSKIDAVIVKLEAMERAKMKRRDVLGRILDGVMEDERMGRDPELQREQMDRLVRDELERWESDDTMSQVSHHHHQATPIISSAQLRPRSSRPPSSLSNEGPDAAASGPAHL

>*Poeciliopsis prolífica*

MSSSRVRPQSQSSRSAQGPDSSEGIEMENIQHQDLGLGGVIGTPSPPSRQAWSRDNPVFEPEEEIMEADWPQASPGRRSVSTASSGSSCSSYNRGGSSAQIPRGGLYPTPTTSARLQDRHEHRSCLKQILQKIRILWGTELMEDSDSSRERYLRNVLREMLTYIGFLITVCVLTYGMVNSNMYYYTKVMSQLFLDTTLSPGDQTTFRSVSTMEDFWKFTQGPFLDGMYWDVWYNNNSLPENQSFIFYENLLLGVPRLRQVKVRNESCSVHEDLRDEVQDCYNIYTPSNEDTASFGPKNGTAWVYATQSEMNSSSHWGQVSKYGGGGYYQDLSRTKEDSANRLQFLKDHLWLDRGTRAVFLDFSVYNGNINLFCIARLLVEFPATGGAVTSWHFQTVRLIRYVSSWDYFVGVCEVAFCLFILYYVVEEVLEIHIHQLHYFKSMWNCLDVLIVVLSVVAIIMNITRTAMARNLLSGLLENYTAHPSFEPLASLQAQFNNLAAVIVFFSWVKLFKFINFNKTMSQLSTTMSRCAKDLTGFAIMFFIIFLAYAQLAYLVFGTQVNDFSTFQASVFTQFRITLGDFDFSEIEQSNPVLGPVYFTTFVFFIFFILMNMFLAIINDTYSEVKADMSQQRCEMEMTDLIKKGCNKALMKLRLKKTPVDEISSGLQQASSKMNFDELRQDLKGKGHTDAEIQAIFAKYDQDGDPELTEHEHQQMRDDLEKEREDLELERNSLTRPSSGRSFPRTQDDSEEDDDEDSGHSSRRRGSSSGGVSYEEFQVLVRRVDRMEHSIGSIVSKIDAVIVKLEGMERAKLKRRDVLGRLLDGVMEDERLGRDTDVHREQTERLVREELERWESDDLGSQVSHTPVGPRARPPSSLSVDGLDVGSNGPVHV

>*Oryzias latipes*

MSATRVRPQSQAGTDKPPRTLDSSEGIEMENIQHQDPGLGGVAGSPSPPSRQAWSRDNPVFEPEEEIMQADWPQASPGRRSAFTASGGSSCSSGHGGYTRAGSSTQIPRGGLYPTQTLNGQQQDRHEHPGCMKRIFQNIRILWGTELMEDGDSNRERYLWNVLRELLTYIAFLITICILTYGMISTNMYYYTKAMSQLFLDAPLSSENPKTFRSLSTMEDFWKYTQGPFLGGMYWEVWYNNQSLPGNQSFIYYENILLGVPRLRQVRVHNETCSIHEDLRDEVQGCYGVYTPSNEDNSPFGPQNGTAWVHTTESKMNASTHWGQVSKYGGGGYYQDLSRTREESGLQLQFLKDHLWLDRGTRAIFLDFSVYNGNVNLVCIARLLVEFPATGGVLTSWQFQTLRLIKYVSSWDYFVGVCEVAFCLFVLYYVVEEVLEIHIHRLHYFKNLWNCLDVLIVTLSVVAIIMSITRAAMGGDLLKGLENYTSHTSFDSLANLQVQFNNMAAVIVFFCWVKLFKFINFNKTMSQLSTTMSRCAKDLVGFAIMFFIIFLAYAQLAYLVFGTQVNDFSTFQGSVFTQFRIILGDFDFFEIKEANPVLGPIYFITFVLFIFFILMNMFLAIINDTYSEVKADMAQHRSEMEMTDFIKKGCTKALVKLRLKKTTVDDISDSLRQAGGKLNYDELLQDLKEKGHTEAEIQAIFAKYDQDGDLELTEHDHQQMRDDLEKEREDLDLERNSLTRPSSGRSFPRTQDDSEEDDDEDSGHSSRRRGSSSGGVSYEEFQVLVRRVDRMEHSIGSIVSKIDAVIVKLEGMERAKLKRRDVLVRLLDGVMEDERLGRDADTHREQMERLVKEELERWETDDVASQVSHPQPATPIGPRPRPPSSLSTDGLDTSANGGTHV

>*Ictalurus punctatus* OX=7998 GN=pkd2 PE=3 SV=1

MSSSRARCQTARAPSSAHAHKPPPAEGLEMERMEPQGEVGLGVPEDQSSPSSNSSSRQAWSRDNPGFEPEDGMEANWSPGRRSLSDTSSSGSSGLGSFTGGSNARIHRGLYPTPPADGLHHEPPSLGARILEKIRLLWGTRLLEERDSSREMYLKNVLREMITYVLFLVTLCILTYGMVSTNMYYYTKVMSQLFLDTPLSRGDATTFRSLSTMEDFWKYTEGPFLNGMYWEVWYNNKSLPENQTLIYYENLLLGVPRLRQVKVLNESCPVHEELKDEVYDCYGVYAAAYEDKKSFGLKNGTAWVYSEESSLGESSYSGEVATYRGGGFYQDLSRTRDESGRQLQELKANLWLDRGTRAVFLDFSVYNGNINLFCIIRLLVEFPATGGAVTSWQFQTVRLVRYVSSWDHFVGMCEVIFCFFVLYYMVEEVLEIRLHRLRYFKSLWNCLDVLIVMLSVPAIIMNIYRTSAVSNGLKFLLENHSTYPNFSPLARLQVQFNNLAAVIVFLACVKLFKFINFNKTMSQLSSTVSRCAKDLLGFAIMFFIVFLAYAQLAYLVFGTQLNDFSTFQACIFTQFRIILGDFDFSEIEESDRVLGPIYFTTFVFFMFMILLNMFLAIINDTYAEVKADMAQQRSEMEITDLIKKGYHKAMVKLRLRKTAVDDISDSLRQAGGKLNFDELRQDLRGKGHSDAEIEAIFAKYDLDGDQELTEHEHQQMRDDLEKEREDLDLERSSLPRPLSGRSFSRSQDDSEEDDDEDSGHSSRRRGSSSGGVSYEEFQVLVRRVDRMEHSIGSIVSKIDAVIVKLEAMERAKLKRREVLSRLLDGVMEDERMGRDTEAHREQMERLVREELERWESDDAISQVSHQQATPVGPGAQPRPRSTRPSSSLSTEGPDTGGNGGGHV

>*Takifugu rubripes* OX=31033 GN=pkd2 PE=3 SV=2

MSSSRVRPQQSPAARMPHGLESGEGIEMENIRHQDLGLGGVIGTPSPPSRQAWSRDNPGFEHEDGIMEADWPPASPGRRSASTASGSSCSSGLGSFTGGGGGGTHAPRGGLYPTPTVDTQQQESHQRRSCMKQILQKIRILWGTELMEDRDSSRERYLRNILREMITYIAFLVTICILTYGMVSANMYYYTKVMSQLFLDTPLSPGDPATFRSLATMEEFWKFTEGPFLNGMYWEVWYNNKSLPENQSLIYYENLLLGVPRLRQVKVRNESCSIHQDLKGEVLECYNLYTPSNEDTASFGPKNGTAWVYASETEVNGSSYWGQVSKYGGGGYYQDLSRTKEESMTKLQLLREHLWLDRGTRAVFLDFSVYNGNINLFCIVRLLVEFPATGGVLTSWQFQTVRLIRYMSSWDYFVGLCEVAFSLFILYYIVEEVLEIRIHRLHYFRSLWNCLDVLIIVLSVIAIIMNITRTAMVGKLLKGLLENHTAHPSFGSLAHLQVQFNNVAAIIVFFSWVKLFKFINLNKTMSQLTTTMSRCAKDLVGFAIMFFIIFLAYAQLAYLVFGTQVDDFSTFQASIFTQFRIILGDFEFSEIEEANPVLGPIYFTTFVFFIFFILMNMFLAIINDTYSEVKADMSQQRSEMEMTDFIKKGCNKALMKLRLKKTAVDDITDSLRQAGGKLNLDELRQDLKGKGHTDAEIQAIFAKYDQEGDQELTEHEHQQMRDDLEKEREDLELERNSLTRPISGRSFPRTQEDSEEDDDEDSGHSSRRRGSSSGGVSYEEFQVLVRRVDRMEHSIGSIVSKIDAVIVKLEAMERAKVKRRDVLGRLLDGVMEDERLGRDTDAHREQMERLVREELERWECDDMGSQVSHPQPATPVGPRPRPSSSMSTDGLDTVTNGSSHV

>*Liparis tanakae* OX=230148 GN=pkd2 PE=3 SV=1

MSSSRVKPQQPPQSQTAMSPHTLDSGEGIEMENIQHQDLGLGGVAGTPSPPSRQAWSRDNPGFEAEEEIMEADWPPASPGRRSVSTASSSSCSSGPGNYTGAGSSTQVPRGGLYPTPTVDARQQDSHDHRSCMKQILQRIRILWGTELMEDSDSSREKYLRNVLREMITYLTFLITICILTYGMVSANMYYYTKVMSQLFLDTPLSAGDPSSFRSLSTMEDFWKFTEGPFLNGMYWEVWYNNKSLPENQSLIYYENLLLGVPRLRQVKVRNESCSVHEDLRDEVLDCFNMYTPTNEETTSFGPKNGTAWVHTAENDMNGSGYVGQISKYRGGGYYQDLSRTKEESATQLQFLKDQLWLDRGTRAVFLDFSVYNGNINLFCIARLLVEFPATGGVVTSWQFQTVRLIRYVSGWDYFVGLCEVAFCLFILYYMVEEVLEIRIHRLHYFKSLWNCLDVLIVTLSVVAIIMNITRTAMVGNRLKGLLENHTAHPSFEPLANLQIQFNNMAAVIVFFSWIKLFKFINFNKTMSQLSGTMSRCAKDLVGFAIMFFIIFLAYAQLAYLVFGTQVNDFSTFQASIFTQFRIILGDFEFSEIEEANPVLGPIYYTTFVFFIFFILMNMFLAIINDTYSEVKADMSQQRSEMEMTDLLKKGCNKALMKLRLKKTAVDDISDSLRQAGGKLNFNELRQDLKGKGHTDAEIQAIFAKYDHDGDQELTEHEHQQMRDDLEKEREDLDLERNSLTRPSSGRSFPRTQEDSEEDDDEDSGHSSRRRGSSSGGVSYEEFQVLVRRVDRMEHSIGSIVSKIDAVIVKLETMERAVLRRRDVLGRLLDGVMEDERLGRDTDVHSELMERLVREELERCESVDVVSQVSHSQAATPVGPRPRPPSSLSTDGLDTSTNGSSHV

>*Sphaeramia orbicularis* OX=375764 GN=pkd2 PE=3 SV=1

MSSSRVKPQQLPQSQTGRVPNRLDSSEGIEMENIQHQDLGLGGVIGTPSPPSRQAWSRDNPGFEPEDEIMEADWPPASPGRRSVSTASSSSCSSGLGSYTGGGSSTHIPRGGVYPTPTVDAQQQDRHEHRSCMKQILHNLRILWGTELMEDSDSSRERYLRNVLREMLTYVAFLITLCILTYGMVSSNMYYYTKVMSQLFLDTPLSVGDPLTFRSLSTMEDFWKFTEGPFLNGMYWEVWYNNKSLQENQSLIYYENLLLGVPRLRQVKVHNESCSVHEDLRVEVQDCYNMYTPSNEDTAPFGPKNGTAWVYTSESEMSGSSHWGQVSTYGGGGYYQDLSRTKEESAVQLQFLKDHLWLDRGTRAVFLDFSVYNGNINLFCIARLLVEFPATGGVVTSWQFQTVRLIRYMSSWDYFVGVCEVAFCLFILYYVVEEVLEIRIHRLHYFKSLWNCLDVLIVVLSAVAIIMNITRTAMVGNRLKDLLENHTAHPSFESLANLQVQFNTVAAVIVFLSWVKLFKFINFNKTMSQLSSTMSRCAKDLVGFAIMFFIIFLAYAQLAYLVFGTQVNDFSTFQASIFTQFRIILGDFEFSEIEEANPVLGPIYFTTFVFFIFFILMNMFLAIINDTYSEVKADMSQHRSEMEMTDLIKKGCNKALMKLRLKKTAVDDISDSLRQAGGKLNFDELRQDLKGKGHTDAEIQAIFAKYDHDGDQELTEHEHQQMRDDLEKEREDLELERNSLTRPSSGRSFPRTQDDSEEDDDEDSGHSSRRRGSSSGGVSYEEFQVLVRRVDRMEHSIGSIVSKIDAVIVKLETMERAKLKRRDVLGRLLDGVMEDERQGRDTDAHREQMERLVREELERWESDDMVSQVSHPQLATPVGPRPRPSSSLSTDGVDNSTNGSSHV

>*Myripristis murdjan* OX=586833 GN=pkd2 PE=3 SV=1.

MSSTRVRSQQTPQTQTARLPNRFDSSEGIEMENIQHQDLGLGGVIGTPSPPSRQAWSRDNPGFEPEDEIMEADWPPASPGRRSVSTASSSSCSSGLGSYTGGGSSTHIPRGGTFKLKYDPFLWGTELMEDSDNSRERYLRNILREMLTYITFLITLCILTYGMVSSNMYYYTKVMSQLFLDTQLSAGDPSTFRSLSTMEDFWKFTEGPFLTGMYWEVWYNNKSLPENQSLIYYENLLLGVPRLRQVRVRNESCSIHEDLRDEVQDCYSMYTPSNEDTASFGPKNGTAWKYTAEGKLNGSSYSGQVSKYGGGGYYQDLSRTKEESAIQLRFLKDHLWLDRGTRAVFLDFSVYNSNINLFCIARLLVEFPATGGVVTSWQFQTVRLIRYVSSWDYFVGMCEVAFCLFILYYVVEEVLEIRIHRLHYFKSLWNSLDVLIVVLSVVAIIMNVTRTAMVSNLLKGLLENHNTHPTFEPLANLQVQFNNVAAVIVFFSWIKLFKFINFNKTMSQLSSTMSRCAKDLVGFAIMFFIIFLAYAQLAYLVFGTQVNDFSTFQASIFTQFRIILGDFEFSEIEEANPVLGPIYFTTFVFFIFFILLNMFLAIINDTYSEVKADMSQQRSEMEMTDLIKKGCNKALMKLRLKKTAVDDISDSLRQARGKLNFDELRQDLKGKGHTDAEIQAIFAKYDHDGDQELTEHEHQQMRDDLEKEREDLDLERNSLTRPSSGRSFPRTQDDSEEDDDEDSGHSSRRRGSSSGGVSYEEFQVLVRRVDRMEHSIGSIVSKIDAVIVKLEAMERAKLKRRDVLGRLLDGVMEDEKLGRDTDGHREQMERLVREELERWESDDTVSQVSHPQPATPVGPRPRPSSSLSTDGLDTSANGSGHM

>*Nothobranchius furzeri*

MSSPRVRPQQPPQSLRSPHKLDPSEGIEMESIRHQDLGLGGAIGTPSPPSRQAWSRDNPGFEPEEGIVEADWPPASSGRRSVSTASSSSCSSGLGSSYNRGGNSGQIPRRGLFSTPTLNTQQQDRHEHRSCMKQILQKIRNLWGTELMEASDSSRELYLRNVLREMLTYITFLITICILTYGMVNANMYYYTKVMSQLFLDTPLSPGNPTTFRSLSTMEDFWKFTQGPFLRGMYWEVWYNKDRLPENQSLIYYENLLLGVPRLRQVKVHNESCSIHEDLQDEVRDCYNIYTSSNEDTSSFGPKNGTAWVYTTESGMNGSSYWGHVSKYGGGGYYQDLFRTKEESASQLQFLKDHLWLDRGTRAVFLDFSVYNRNINLFCVTRLLVEFPATGGVETSWQFQTVRLIRYVSSWDYFVGVCEVVFCLFILYYVVEEVLEIHIHRLHYFKSMWNCLDVLIVALSVVAIVMNITRTAMARNLLSGLLENHTAHPRFEPLANLQVQFNNMAAAIVFFSWVKLFKFINFNKTMNQLSSTMSRCAKDLVGFAIMFFIIFLAYAQLAYLVFGTQVNDFSSFQASILTQFRIILGDFNFPEIEENPVLGPIYFTTFVFFIFFILMNMFLAIINDTYSEVKADMSQQRSEMEMTDLIKKGCNKALMKLRLKKTAVDDISDSLRQAGGKVNFDELRHDLKGKGHTDAEIQAIFAKYDQEGDPELTEHEHQQMRDDLEKEREDLELERNSLTRPSSGRSFPRTQEDSEEDDDEDSGHSSRRRGSSSGGVSYEEFQVLVRRVDRMEHSIGSIVSKIDAVIVKLEGIERAKLKRRDVLGRLLDGVMEDERLGRDTDAHREQMDRLVRDELERWESDDTASQVSHPQLATPVGLRPRPPSSLSTDGLDASANV

**Used for the Weblogo**:

O.latipes LDSSEGIEMENIQHQ

S.orbicularis LDSSEGIEMENIQHQ

P.prolifica PDSSEGIEMENIQHQ

M.murdjan FDSSEGIEMENIQHQ

L.tanakae LDSGEGIEMENIQHQ

T.rubripes LESGEGIEMENIRHQ

N.furzeri LDPSEGIEMESIRHQ

D.rerio PPPHEGIEMEKMHHE

I.punctatus PPPAEGLEMERMEPQ

. **:*** :. :

**Input sequences: *Caenorhabditis***

>*Caenorhabditis remanei*

MSFRTTLVQNISDLLTVDILDRGSTTKILFKKNFTFEFNRSDAPGNSKMRNVPKIQKIFWNLLEDSRIFEFPPKWKILNSLQNSKVFLATLSLSCRYIYATSKELENFDTVGTISTYGGGGFVQRLPVSGSTEAQSAIATLKANRWIDRGTRAIVVDFALYNANINLFCVVKLLFELPASGGVITTPKIMTYNLMTYQASSGTRMIVFEGIFCGFILFFIFEELFAIARHRLHYLTQFWNLVDVALLGFSVATIILSMKRTKTATNRVNSVIENGLTNAPFDDVTSAENAYLNIKACAIFIAWVKVFKFISVNKTMSQLSSTLTRSAKDIGGFAVMFAVFFFAFAQFGYLCFGTQIADYSNLYNSAFALLRLILGDFNFSALENCNRFFGPAFFVAYVFFVSFILLNMFLAIINDSYVEVKAELARKKDGEGILDWFMNKVRGLTKRGKRPDAPGEDATYEDYKIMLYRAGYAEKDINEAFTRFNVTTMTEHIPEKMAEDIADEVARVTEQKRNYMENHRDYANLNRRVDQMQESVFSIVDRIENVNVTLQTIEKQRIQQQDGGNLMDLSALLTSQVRNRESARRQTITSIADKKEE

*>Caenorhabditis brenneri*(*Caenorhabditis sp. CB5161*)

MSSSEVSEFRMIEDDDDVSKDVTWLESDSSRLREKQFLLSCERGDIGSVRKLLAGISTENFNINCLDPLGRNALLIAIENENIEMIELLLDHNIETGDAILYAIGEENVEAVEIIVEHLEKMDKFDAERQGVEITEHSAFTPDITPIVLAAHKDNYECIKLFLDKKGTVPHPHDVRCSCPECYVAREEDSLRLSRSRINAYRALTSPSLICLSARDPILYAFELSWELKRLSFIENEFRTDYEELSQKCQKFCVHMLDQVRGSKELEVVLNHTTNAWHDVTSANYGNPEKLARLKLAIQLSQKRFVAHPNCQQLLLDIWYEGVESVRCTNFIYKTIFYFLGMMSFPLFSLVYLLAPHSSMGQFAKKPFIKFLSHSGSYIFFLILLIMASQRMNVIDNLLRTDDVDRKETRGPPPTVIECAIFLWVLGLIWVEIKQLWECGLYNYCRNLWNILDFITNSLYLCTTALRVVSYVQVEKEARQANSVHIARHLPRRDWDAWDPTLLSECFFATANIFSSLKLVHIFTVSPHLGPLKISLGRMVIDIVKFFMVYALVLFAFACGLNQLLWYYASMRQNECNLYEQYKNEKSLSYKYEHLKESCDDKYKSCSSIYHTAETLFWALFGLVDLTHFRLKEDHFLSEWTGKTIFGSYCCCSIIVLLNMLIAMMSNSYQYISDQADIEWKFARSRLFLEYFDDTATLPPPFNIVPSPKSIYYCLNYLTKKLCNCTKLQQPSKQKSMRNQKILRSVNDRENNYRDISAFRYELLGILRNAGYQTGHTDINQKTSSRNKKKTAMAERRLKNSALLHQEFPVPQMFQSGQRNMSISSIQSNGRLKPSFLPSTSKLSWNNLRVKASRLSKSKSIDTTHLDVTRLQALSKKSPLQKQAHTSFETTSLNDDTDELL

*>*Caenorhabditis briggsae

MNYDAAGDRWANPPQPVAAAEHAPAAFDHSMVSEEYEHDKKKNPAPKQGVSFTQALLASGHEKSDGKIKLTASSFMEVGSYAVFLIVLVYVAFAQNSIQSYYYTKVMSDLFVSASGANGAPAFGSCTSMDNIWDWFSQVLVPGIYWTETSNSTDNENMIYYENRILGEPRIRMLKVTNDSCTVMKSFQREIKECFANYEEKFEDREMVADGSVDAFLYATAKELENYDTVGTISTYGGGGFVQKLPVSGSTEAESAIATLKSKRWIDRGTRAIVVDFALYNANINLFCVIKLLFELPASGGVITTPKIMTYNLMTYQSSAGTRMIVFEGIFCGFVLFFIFEELFAIARHRLHYLTQFWNLVDVALLGFSVATIILSMKRTKTATNRVNSVIENGLTNAPFDDVTSAENAYLNIKAVVVFIAWVKVFKFISVNKTMSQLSSTLTRSAKDIGGFAVMFAVFFFAFAQFGYLCFGTQIADYSNLYNSAFALLRLILGDFNFSALENCNRFFGPAFFIAYVFFVSFILLNMFLAIINDSYVEVKAELARKKDGEGILDWFMNIVRGLTKRGKRPDGPSEDATYEDYKIMLYRAGYAEKDINEAFTRFNVTTMTEHIPEKMAEDIADEVARTTEQKRHYMENHRDYANLNRRVDQMQESVFSIVDRIENVSATLQTIEKQRIQQQDGGNLMDLSNLLTKQVRNRE

SARCQTITALADKKEE

*>Caenorhabditis elegans* MNYGAADERWANPPQPVAAAEHGPSFDHSMVSEEYEHDKKKNPAQKEGISFSQALLASGHEKSDGKIKLTARSFMEVGGYAVFLIVLVYVAFAQNSIQSYYYSKVMSDLFVASTGASGAPAFGSCTSMDNIWDWLSQVLIPGIYWTETSNSTDNENMIYYENRLLGEPRIRMLKVTNDSCTVMKSFQREIKECFANYEEKLEDKTMVGDGSVDAFIYATAKELENLKTVGTIASYGGGGFVQRLPVAGSTEAQSAIATLKANRWIDRGSRAIIVDFALYNANINLFCVVKLLFELPASGGVITTPKLMTYDLLTYQTSGGTRMMIFEGIFCGFILYFIFEELFAIGRHRLHYLTQFWNLVDVVLLGFSVATIILSVNRTKTGVNRVNSVIENGLTNAPFDDVTSSENSYLNIKACVVFVAWVKVFKFISVNKTMSQLSSTLTRSAKDIGGFAVMFAVFFFAFAQFGYLCFGTQIADYSNLYNSAFALLRLILGDFNFSALESCNRFFGPAFFIAYVFFVSFILLNMFLAIINDSYVEVKAELARKKDGEGILDWFMNKVRGLTKRGKRPDAPGEDATYEDYKLMLYRAGYAEKDINEAFTRFNVTSMTEHVPEKVAEDIADEVARMTEQKRNYMENHRDYANLNRRVDQMQESVFSIVDRIEGVNATLQTIEKQRVQQQDGGNLMDLSALLTNQVRNRESAARRPTITSIADKKEE

**Used for the Weblogo**:

C.remanei TQFWNLVDVALLGFS

C.brigsae TQFWNLVDVALLGFS

brenneri AIENENIEMIELLLD

C.elegans LTARSFMEVGGYAVF

::.: :

**Used for PhyML:**

C.remanei WNLVDVALL

C.brigsae WNLVDVALL

brenneri NENIEMIEL

C.elegans RSFMEVGGY

. :::

**Input sequences: *Drosophila***

>*Drosophila melanogaster* OX=7227 GN=brv1 PE=2 SV=1. IPR013122﻿, PKD1_2_channel. IPR003915﻿, PKD_2

MAKKFSNIIFYLTLIIIAICVACIWMFSGFRHEYGKFNMILVAFLAVTLLQILIFTPIKFTIMSLDAAFWPAHQAPETPDENANVDTFMDNLRLRLRTLRSELMITERHRNERVNLKYRLITEELWLTGKLFLVYFFMALAFFDELLYFNTEATELLFQCNHGDAFGLLSVGDVPDIYFFVVSSLVLAFTDGKNTSGGAPWIHAEGTRLLGVVRLRQLRTESNRLGLSLPVFTERDFSESWTLPYERVPYTDKYWPIYTPWLPSVSVARDNLLMGINHVGHMFNYPESKGYKVLLSDTRHKSLKIIDYLMKKNWLDANTTALFMDFSLYNADANTFTVCTLWVEKFPYQYPDGHTRIESHTFVEQLREFTKFGMLMVFVFVVTWLQFTKAFFLKVWYDPRQLKTLWVQVDAMIVALSLVVGMIMSVRDNLVQKMIKSVEIAVVVDFLDFREPAQLAYLEDVVTGLAVALVTMRLWKVLQFSATFQLFTKTIAMAWDALLCTFVITVIFIIAIGIATVTINGNYTSNFRDFPKGIVTVTCFAFGYTNLVYPPDIFYGGEWLGILLYTIMGFVVKYMLINLIVSMMRDQMASVKADRDKKVRQRITFWQFLRVEYAHFINYIVKVFHCQKGYQRKNRTVAQNIQRKLNSNELKRRKTKISSIYSETIYVMPIDKDLEQMKYRERIERTFTLAAILHTQMELMERLMFGDEEGNLPSLDQEDEQVPETEED

>*Drosophila ananassae*

MQKIKGGPFLKKMFDEMQAKRNGTLKPSSRKLFIYTGHDSTVVNILSALKVWERQLPRYSSMILFELHKNKTTGCAFCIWKFSGFSHEPVKVNKMMIVFISVCLVQIVIVVPIKFAIISLDAALWPPHQATISTDSIARVETLMGNLRRKLQSLKSQLMITERHRYERLNLKYRLITEELRVFGRLFFILLIFVLVIFDNLLYYNNKITQDLFQHNHTGTVGLSTVFLLPNIYLFLESSIIRAFTERGDFGGAPWVHAESTKMLGSVRLRQLRTVDRHVGLQDPVFDVRDYSEGWKLPYAREAYTDKFWKIHQPWVMRESTVSEHILFRINHYGHFISYPESIGYQNLLSDSRKKSLIILRYLKRKSWLDRNTSALFIDFTLYNADGNVFSVCTLWLEQFPFGSSVTHMTVDSVVFVERMRDFSSFGMLVLFIFLICWLQFAKLFFAKVWYEPGRLRNIWTKLDAITLTFSVLLVGAMARRDKLVQQMIRDLETSVNVEFIDFQRPLRFNDACNVFMGFTVALITLRLWRVMQFARTFQLFNKTLRMAGGTLVSTFIVTAVFLMSIGTSAVVTNGNFSPTVNNLKTGIISVASFSFGYSIALRDFLRGGKWLGIVLYAVLGFVVKALLLNMIMSMLERHLADAKAQRDRKNIHRITYWEFLRVEYADAIKFIMKLFHRKRGYRRNNRTVAQNIQRKLYNQERIGPKAKRRKNFSFTFESREPVDQKFLQLLYRERIEKTFVVFAILKTQIELLERLKFGDEEGNLSESEEENISEEEMDPEPDPEPPPPPPPPPPPWHHGIVINR

>*Drosophila mojavensis*

MWSKVVGLLKRSVLLIATIIIMVVCLICVSLLSGYNHKTKKVEGIVIHSFLVMIFLFVLCDPIKFLILAVDRAWWPRRRDSYSVDRTAMIHTRLHYLKMRLKILRSQLVATDKHCNESLNLLYQSIANDLWLYGRYFFLLMCLVLVTWDQLLCYNTKHMESLFVRNNSHGWGLEKVYSFDDFYKFLESTLVEPFDPNFEESGSRWWIYGDHTIKLGGVRLRQLRVKKGYHMGWKDLKYEDRDYMSQWELPYQRLPYTNKYWKIFTPWLSIYPTLTSEEKFFLNFGNQGYAHDFSELYGYVTLLARTAKSSRLVIQYLKDNKWLTHSKTCAIFMDFTLFNMDANMFTVATLALEMTAYGTIEYAVHIESSKLLMLDEFDSLLKILIVFLYILVFVQFGKSVIIMLWCEPKKLRSTWNKLDLVIILLNIALICLLITHQILLNNRLLSVENASVLVFLDFRIPVRVHIAIEVILGFLVCLTTLRLWRVLQFARVFQLIAATLYAAWKALASTAMLILIFLFAFGMAVGIINGNYTIGFASLTRSIISSVCFALGFKRQLNPADLLHGGVYLGLIFYVILAFFIVIVMMNLFITTLRDYFGHIRRKMEARFVFNQITFLQFLRAEYAPIFRYFLELPCFKRGYKRHNRTVHQNIEIALQARSKGYKPKLVFHAQSEDHLNQLRNIEKKEEQLKHDKYKERIERMHTIAALMQTQLELLSHLLFTEAMLARDSEDSMYEASDSASEQEAKYRR

>Drosophila pseudoobscura pseudoobscura

MGALWHLSRGQKIQGDPGVVHFAIDLACWPPRMRMTKPDEAQAKRHNRMDYLNLRLRSLRSQLMITERHRNEKLNLKYRTIVHELWVYGKVFLIHLFLNVALDRDDLYYNYKSIQTLITYNTTSTIGLSHVLFIHQLYPFIEHTLVNAFLSDDHHSLNANWVNSNSTKLLGVVRMRLLRNTGEQYGLNDPEFTEKEYSHGWSPSYFDEPYTDKSWSIYLPWLPMEDKHTFWEKIFLNIKQYGFFAAYPELKGYVVTLRDKKIDSIKVLTYLFQNNWLDGRTAAIFFDFTLYNVDADLFSVCSIRCENLPFGIIRSSMDINTVSMTMSAETVPWTIFIWFIAYVAVLLQLGVAVIVPLWFEPKRFKSFWVKVDVLILLLSMSVLIMKGVKSGITTKLLALLEVASTHDFIDFRVPARLNDWATIAMGCLICLVTLRLWKVMQFARIFQIFTMTLSLAWSALFSTACVICILMMAFGIVMVCINGNNRKNFRSLLHALATIMCFTFGFSSHVDPEDLFSGGELIGIVIYTIMAFTISILLVNVFVSILNNYFTAAKAVRDRQRKDEISFWEFLRVEYADAIRIVREIFLLQPSYKRRNRTVSENIKRKLDLQERANSSARLKRGYNYVATPRWKSVIESEEHEHKQQALHRDRISRTYTIAAILQTQLEIAERSIFGDKDGNLRNESDEDEGEAPSDDQNFPRQYQG

>*Drosophila erecta* OX=7220 GN=Dere\GG15821 PE=3 SV=1

MSFKNISRKDLTCFGVTATVLLVCFILVSMFSGFMHEPQRFKTMLVTIVLVFFFQYLVLEPFRFFVLGIDYATWPQEDQPYRPEEGAPTLNHIGYLKIRLRSLRSELLITEGHTHETLNQRYKHIAGDLLLYGSYFVALMLLVVLQEDQSHYFNTHNMQRLFWDNTSVTYGLSQVFFLYQVHSYVYITMIDAFFTSEYYGYEGWWAMEQWRNIGVVRLRQVRPVNCHIGLGTPKWDTNTYAPEWLLPYHRLHYTEKFWRIYDPFVPAKYEPSFLNGVLLNYDHHGYMLNYPEVGGYVVLMMSTKVNCVKQIQYLQSSYWLDKNTSALFIDLTMYNADANLFTLITLRVENTPFGIQLPRVHVDTVRMLGSLETRSNLVLFILFVHTILVILFARGLLSKIWHHPASVHEAWTMVDLTICVLNVVLMVLAIMRDIETSTLLEMVETSTKGQYLDFQRPLRLHQLLSIVKGFLVCITTLRLWKVLQFASVFQHFTHTLFSAWRAVVSMGVIIMVVLMAIGITLAVPNGNNSVVFSHMVQSVVTCMWYSMGFNGDIRPADFFHGGRILGIIVYLTLVFLLAIILMNVFASVIYDYFNETSRNLKEQSHRSSITFLEFLRVEYADIFGATFRCLRKTYNRRGHTVAENVELELNRRELIKAKSDSLKSTKELKRARLSAEEQSADYRIRGEKLFKLRAILKLQIEILERLVFGDKDGNLPTPPGSDSDPEDMPEMYRKRR

**Used for the Weblogo:**

D.melanogaster FMDNLRLRLRTLRSE

D.pseudoobscura RMDYLNLRLRSLRSQ

D.erecta HIGYLKIRLRSLRSE

D.mojavensis RLHYLKMRLKILRSQ

D.anassae GFSHEPVKVNKMMIV

: :::. :

**Used for PhyML:**

D.melanogaster NLRLRLRTL

D.pseudoobscura YLNLRLRSL

D.erecta YLKIRLRSL

D.mojavensis YLKMRLKIL

D.anassae HEPVKVNKM

:::. :

**Input sequences: *Schizosaccharomyces***

>*S.pombe*

MRLWRSPLLLLVVVVELFSWADALTRFISADSLSTCMTDSQLSASKLYASYFPDNQSVAFDISIQSLVSTNVSIDVDISAYGIEIKKVIDPCDMEISGFCPMQTGNIALSGSHTLTGEALSILDSIPSIAYTVPDLDAVVTINIYESDTNTQLACVRTTVQNGRSVYHRAVYWVMCMVIGIPLLIFLLISPVLQTPALWEIVETMITLFQFAQIQALYSMMATSLPAIIYSWGRNFMWSMGIIRIGFMQDVFTWYVKSTGGTPSTLVDLGIHANVALAKRGIDLGSLAKRATTTVTTSTSDSITLRGIKRISYMMGIETTNFFATGFSFFIILLFFSLLVAMASRFIVEMVLLASRNQALKKQRIRLYWKSISKGFFYRVIFVGFTQMSVLSMWEIYTRDSSALAFLSMYVIVDMAVLLCYAFVRTIQIIRKTGPYSHPDVLYNLYSDTQHLMRWGFMYVQLDVRFFYFTFPLLLITLVRSMFIGFGQGSPKVQGCAMFGISVVVFALMVILRPYATKHMNTLHIGVALMNLISGSFILVMCQAFYVEELARQVIGIIFFALNAITMLLLILGIFIRTLIVLFRKSGHGTYYRILDDQSEKATSYNKSIKDMSSSDMAFSDPAYSGTTLRSSVDLNTPEYPFSNRNDSDSTFTNNKYVSPWDAIEEASYANLRGNTDVEQPFMESDYTRISENNNNAERRRKPLPNNAFR*

>*Schizosaccharomyces cryophilus*

MRLLQSPILSLFLIFFAIQSVRAKPKQLYVKSLTTCMTDSQFSASTLDAVYYPGNTSIYFDVSARSLVSSNVSIHVNVSAYGFKLEKTIDPCDMNIAGFCPMQAGNIILQGNHDLTGEVTKWVNELPSIAYTVPDLDAKVTVNMYDTSSNERIACVKTNVENGRSVYYHAVYWILCMVIGVPLLLFLLLSPIFQTPAMWEVFENMVALFQFAQLQGMYAMMASPLPGIVFSWGRNFIWSMGIIKIGFMQDVFTWYVKSTGGDPSVLVEMGSHGNVGLAKRGLDSASLFSKRDSSSGSSSSSNSGTITLRGIRRLSYMMGIETTNFFITGFSFFIILLGFTLCIAILSRIVLELYYLIAKDRALKRQRAREYWKAIAKGVFYRAIFLGFWQMSALCMWEIYTRDSSALAFLSMYVIVDMAILLLYAFARTMQIIRKTGAISDPDALFNLYSDTQHLMRWGFMYVSYRHRFFFLSFFLLIVILIRSMFIGFGQDSPVVQGCAMFAISVVVFFFMVVARPYATKHLNSMHIGISIVNMISGVFLLIMCKAFTVNELARQVIAIVFFAINAITMLVLIVGIFIRNIVVLLRRTKNGTYYRILDESNEKSSSLKDFPKHPSSEMNVFHDPGFTGTTLRGSTDLRTTDNPFSSHDDSTNTYSNYKYNSPWQAIEEDSYAKLHGTNTNEFYRDPSAIESLGYNGGHRRRPLPEPSFY

>*Schizosaccharomyces octosporus*

MRLLQGLFFSFFLFFCIVQSTVAKSRQLYVKSLTTCMPNSQFSASTLDAVYYPDNTSIYFDVSARSLVSTNVSIHVNVSAYGFTLEKTIDPCNMNIAGFCPMQAGNIILEGNHNLTGEVIQWIDDLPGIAYLVPDLDAKVTVNIYDTDNNQRIACVMTNVENGRSVYYHVIYWVMCMVIGIPLLLSLLLSPVLQTPAMWEVFENMVVLFQFAQMQALYAMMASPLPAIIFSWGRNFIWSMGIIKIGFMQKVFTWYVKSTGGDPSVLVEMGPHANVGLAKRGLETASYFSKRASNSTLGTGSSSSSNNGTVALRGIKRISYLMGIETTNFFITGFSFFIILLGFTVCIAILSRIVLEVYYLVARDKTLKRQRIREYWKAITKGFFYRAIYLGFWQMSVLCMWEIYTRDSSALAFLSMYVIVDMAVLLLYAFVRTMQIIRKTGAFSDPDALFNLYSDTQHLMRWGFMYVSYRHRFFFLSFFLLVVILIRSMFIGFGQSSPIVQGCAMFAISVVEFFFMIFARPYATKHLNSLHIGIALVNLISGIFILIMCKAFTINELARQVIGIVFFAINAIAMLLLILGIFIRNVIVLLRRSKNGTYYRILDDSNKKSTSIQDFSKHPSSEINVYHDSGLTGTTARGSTDIRTTDNPFSSGDDRFPNHKNMPWQAIEEESFARLHETKPNELYSDPSDVASSRGYRRRPLPETPFY

>*Schizosaccharomyces japonicus*

MYLRYALISITAYFFAFLSPASAVRVLRSRSLATCSSNSQLSASTFNVDFFPANQSIVFDLSLQSSMSSNITIVANIYAYGISISPITVNPCNLKFSGFCPITTGNIDLEGSYTISGSALDTLKSIPGIAYMVPDLDASVIVRINSTDTGEELACVEASFSNTRSVHSRAVYWVMCVIIGVPLIVFLFFSSAFQTPEMWEICASFLAMFHFSQSQALYGFLAIKLPIVVESWAQNFNWALGIIRLNFMQSIFTWYIKSTGGTPSVLTKLGDHANVILYKRAIEPAVSLWKRTSYSSSSSTSSTTLHGIDRVAYRAGIETTNLFMTGLAFFVILLGFTLILVIVFRVLLELGLLFRSIHDSRALELRVRCNAISKGFFYRVVYVSFTQMAVLSMWEIYTRDSTGAAILGMYIIVDIAILLLWAFIRTVQAVRKMGPFLHNEAVYNLYSDIQHLMRWGFMYVPYKVRYFFFALPILIVSLVDAMFIGFGQGHPVVQGIARFVISIIVFLVYLVLRPFATKHMNAMHCGIAFMGLVSGVFILIMCEAFEVNELVRQVIGIVMFALNALTLLFMILGVYIRALVLLCRRHPKGTYSRMIEVPTDSSETKVSTGFDNSDPFSADMHGKEGPLEPSSRSHGSSDVRRVDAARTYPWEVVEDASYASLHGPAELQRPHVRNAEYSESSVTGRMSDAGNSSAPSYTNRDPYTRTANR

**Used for the Weblogo:**

>S.pombe DISAYGIEIKKVIDP

>S.cryophilus IWSMGIIKIGFMQDV

>S.octosporus IWSMGIIKIGFMQKV

>S.japonicus LYGFLAIKLPIVVES

**Used for PhyML:**

S.cryophilus MGIIKIGFM

S.octosporus MGIIKIGFM

S.pombe AYGIEIKKV

S.japonicus FLAIKLPIV

*:: :
