# Supplementary material for: Analysis of the SNARE Stx8 recycling reveals that the retromer-sorting motif has undergone evolutionary divergence
Source: PLoS Genet. 2021 Mar 31;17(3):e1009463. doi: 10.1371/journal.pgen.1009463 (PMC8041195; doi:10.1371/journal.pgen.1009463)
Supplement: S6 File — Sequence alignment of Vps26 and Snx3 sequences from S. pombe, S. cerevisiae and H. sapiens. The conserved residues are highlighted (DOCX) [file pgen.1009463.s014.docx]

**S6 File. Conservation of the Vps26 and Snx3 residues relevant for interactions.** The conserved residues, according to Lucas et al., [1], are highlighted.

**Vps26**

S.cerevisiae      ---MSIFFKPPIDIEILFDNEESRKHVDIATRSSNSSYKSMKESLPVYEDGESLGGIVTL         57

H.sapiens         MSFLGGFFGPICEIDIVLNDGETRKMAEMKTEDG------KVEKHYLFYDGESVSGKVNL         54

S.pombe           ---MDYFFKSPIDVDLHLDNEEERTFVDYEFEQG------RKDKAPIYESDETVKGTVMI         51

                     :. **    :::: ::: * *. .:   ...        :.  :: ..*:: * * :

S.cerevisiae      RV-RDSKKVDHLGIKVSVIGSIDMLKSHGSGNSSSKKVTSSTSSSSSNGSVDVRKNSVDQ         116

H.sapiens         AFKQPGKRLEHQGIRIEFVGQIELFNDKSN---------------------------THE         87

S.pombe           RL-KDGRKLDHDGVKIEFIGQIENTYDKGN---------------------------IHE         83

                   . : .::::* *:::..:*.*:   .:..                            .:

S.cerevisiae      FLCQSYDLCPAGELQHSQSFPFLFRDLSKRYESYKGKNVDVAYYVKVTVMRKSTDISKIK         176

H.sapiens         FVNLVKELALPGELTQSRSYDFEFMQVEKPYESYIGANVRLRYFLKVTIVRRLTDLVKEY         147

S.pombe           FTRSVQELASPGEMRHAQMFEFEFKHVDKPYESYIGKNVKLRYICRVTVSRKMKDVIREK         143

                  *     :*.  **: ::: : * * .:.* **** * ** : *  :**: *: .*: :

S.cerevisiae      RFWVYLYNSVTTAPNTLSANETKATTNDIAGGNYAADNASDNTQTKSTQGEAADVNQVLP         236

H.sapiens         DLIVHQLATYPD------------------------------------------------         159

S.pombe           DLWVYRFENEPE------------------------------------------------         155

                   : *:   .

S.cerevisiae      ISHSNNEPKPVRLD**I**G**I**ENCLHIEFEYAKSQYSLKEVIVGRIYFLLTRLRIKHMELSLIT         296

H.sapiens         ------VNNSIKME**V**G**I**EDCLHIEFEYNKSKYHLKDVIVGKIYFLLVRIKIQHMELQLIK         213

S.pombe           ------TNSLIRMD**V**G**I**DECLHIEFEYSKNKYHLKDVIIGKIYFILVRIKVQRMEVSIIR         209

                          . :::::**::******** *.:* **:**:*:***:*.*:::::**:.:*

S.cerevisiae      RESSGLQTSNVMTDSTAIRYEIMDGSSVKGETIPIRLFLSGYDLTPNMSC--NYFNVKNY         354

H.sapiens         KEITGIGPSTTTETETIAKYEIMDGAPVKGESIPIRLFLAGYDPTPTMRDVNKKFSVRYF         273

S.pombe           RETIGTSPNQYSNSETITRFQIMDGNPNRGETIPLRMFLNGYALTPTFRDVNKKFSVRYY         269

                  :*  *   .     .*  :::****   :**:**:*:** **  **.:    : *.*: :

S.cerevisiae      LSLVIIDEDGRRY**F**KQSEITLYRTR-----------------------------    379

H.sapiens         LNLVLVDEEDRRY**F**KQQEIILWRKAPEKLRKQRTNFHQRFESPESQASAEQPEM    327

S.pombe           LSLILVDEDQRRY**F**KQSEITLWRRRDEHE-------------------------    298

                  *.*:::**: ******.** *:*

**Snx3**

S.cerevisiae      MPREFKSFGSTEKSLLSKGHGEPSYSEIYAEPENFLEIEVHNPKTHIPNGMDSKGMFTDY         60

H.sapiens         -------MAETVADTRRLITKPQNLNDAYGPPSNFLEIDVSNPQTVG----VGRGRFTTY         49

S.pombe           -----------MDKLSRPEIRQQTTQQMYDVPENILEIDVINPQTHG----IGRNMFTTY         45

                               .         . .: *  *.*:***:* **:*       .:. ** *

S.cerevisiae      EIICRTNLPSFHKRVSKVRRRYSDFEFFRKCLIKEISMLNHPKVMVPHLPGKILLS----         116

H.sapiens         EIRVKTNLPIFKLKESTVRRRYSDFEWLRSELERES------KVVVPPLPGKAFLRQLPF         103

S.pombe           EIVCRTNMPYFRLHNSSVRRRYSEFEKFHDMLERES-----GRVSIPPLPGKIFTQ----         96

                  **  :**:* *: : *.******:** ::. * :*       :* :* **** :

S.cerevisiae      ----NRFSNEVIEERRQGLNTWMQSVAG**HPL**LQSGSKVLVRFIEAEKFVG----------        162

H.sapiens         RGDDGIFDDNFIEERKQGLEQFINKVAG**HPL**AQNER-CLHMFLQDEIIDKSYTPSKIRHA        162

S.pombe           -----RFRDDVIEERRQGLENFLRLVAG**HPL**IQTHSRVLSSFLQSPEF----KPTP----        143

                        * ::.****:***: ::. ****** *.    *  *::   :

R^9^R^10^Y^22^: Vps26-Vps35 Interface

H^132^P^133^L^134^: Vps26-Snx3 Interface. Important for cargo binding

E^30^D^32^: Vps35-Snx3 Interface

1. Lucas M, Gershlick DC, Vidaurrazaga A, Rojas AL, Bonifacino JS, Hierro A. Structural Mechanism for Cargo Recognition by the Retromer Complex. Cell. 2016 Dec 1;167(6):1623-35 e14.
