# Supplementary material for: Analysis of the SNARE Stx8 recycling reveals that the retromer-sorting motif has undergone evolutionary divergence
Source: PLoS Genet. 2021 Mar 31;17(3):e1009463. doi: 10.1371/journal.pgen.1009463 (PMC8041195; doi:10.1371/journal.pgen.1009463)
Supplement: S7 File — Relevant sequence information about the two-hybrid analyses is provided. (DOCX) [file pgen.1009463.s015.docx]

**S7 File. Sequence information related to the two-hybrid analysis.**

- Primers used to amplify Stx8 without the transmembrane helix (*stx8**), as an *Nde*I/*Sma*I fragment

**FsvNde-F**: atatatatCATATGTCAAATTTGCTTCTGATCATTG

**FsvWOtmSm-R:** atatatatCCCGGGCTATTTAGCCTTTCGGCTGACCTTGTT

Protein (Stx8*)

MSNLLLIIDSVSQKIRDRRKLEEFGQNPDEEIESSLKDVRQELQKLNEEQSRLEKNAQIPEYRVRESEAFLIRMQRRLESAEEEFEKQRRASSIPADGTSAFSANPQVASTNNKLTPLPSLQKTTSSSEGSDIEMEAMYPVDGNDPDPINVNVLAQMHQQMLNEQEESLGGIEASVQRQKRMGYAMNTELSEQNVLLDNMNNDADRIERRFDHAKNRLNKVSRKAK

- Primers used to amplify Snx3, as an *Nde*I/*Sma*I fragment

**Snx3Nde-F:** atatatatCATATGGATAAATTAAGTAGACCGGAAATC

**Snx3Sma-R:** aattaattaaCCCGGGTTATGGCGTAGGCTTGAATTCCGG

- Primers used to amplify Vps26, as an *Nde*I/*Sma*I fragment

**Vps26Nde-F:** atatatatCATATGGATTACTTCTTTAAAAGTCCAATC

**Vps26Sma-R:** aattaattaaCCCGGGTCACTCATGCTCATCCCTTCTTCT

- vps29-vps35-vps26 custom gene

tttatataCC**ATG**GTTGTTTTAGTTATTGGGGATTTTCATATCCCAGACCGAGCTCCTAAGCTCTCTGAGAAGTTTCGACAACTTCTAATTCCCGGAAAAATCAGTCAAATCATATGCTTGGGAAACCTAACTAGCACAAGTGTATATGAATATTTGAAGCATGTTTGTTCTGATTTGAAGCTAGTTAAAGGTGCTTTTGATATTAGCAGTAAGGCTCCAATCGCCGGGAAAATAACTCTTGGTTCTTTTAAAATCGGGTACACAAACGGTCACTTGGTAGTACCTCAGGACAGTCCAGAGGCACTAAGTATTTTAGCTCGAGAAATGGACGCTGATATCCTTTTGTTTGGAGGCACACATAAATTTGCAGCATATGAACTTGACGGCTGTTTTTTTGTTAACCCTGGATCAGCTACAGGTGCACCTAATGTTTCTGCTGTAGAAGATGATGAAAAAATCGTTCCAAGCTTTGTTTTAATGGATGTTCAAGGAGCAGTTTTAATACTTTATGTTTATAGGATTTTTGATGGAGAGGTCCGGGTTGAAAAAATGCAGTACAGAAAACCAGAA***gctgcaggtggagctgcagctgcaggtgga*ATG**AACGGAATTAATACTGCTAACGAAGAAATTACAAGGAGCTTGGAAGAGTCCTTAAATATTTGCAAGCAGTCTAGTAGGCTGATGCAAAGAAACTTGCAGACAGGACGATTGATGGATGCATTTCGTAATTGCAGTATTAGTCTCGTGGAAATGAGAAATTCTGCACTTACTCCAAAACAATACTATGAGCTGTATATGTTTAATATGGAAAGCTTGCGTTTACTTGGGGGCACATTGCTGGAAACGCATCTGAACGGTACTCACAACCTAATGGACCTTTATGAACTTGTTCAATATGCCGGTAGTATTGTCCCTCGTTTATATCTCATGATAACCGTTGGAAGCGCTTATTTGGAAACGCCTAATGCCCTTGTACGTGAAATTATGAATGATTTGTTAGATATGTGTAGAGGTGTTCAGCATCCGTTAAGAGGTCTTTTTTTGCGCCATTATCTGCTTACCCAAACAAGAAAAGGATTACCTTTGGGATCAGAAGATGAGGAAGATGCATCTCGCAAAGGAACCGTACTCGATTCTGTTAAATTTTTAGTTATCAACTTTACGGAAATGAATAAATTGTGGGTTAGAATCCAACATCTTGGTCCAATTAAAGAATTTTCCAAACGTACCCAAGAAAGGAATGAGCTAAAGGTTCTTGTTGGGCTGAATTTGGTTCGATTATCTCAGTTAAACCTCGATATAGACACTTACCGAGATCATGTCCTTCCTGCCATAATCGAGCAAATTATTGAATGTCGGGACTCACTTGCACAAGAGTATCTTGTTGAAGTAATTTGTCAGGCGTTTTCTGATAATATGCATCTTCAAACCCTCGATACATATTTCGGTACGGTCATAAAGCTTTCTCCAAGCGTAAACGTAACCCAACTTGTGGTCGCTATGTTGAATCGCCTTACGGACTATGTCCAACGTGAATATGAATCTGATTCTTCTAATGAAGATGAATCTGAGACTGTAACCGAAAAATTAGGAGACATAAAAATTAATGAGGAAGTACAGCAAAAAGACGAGCAAGAATGCCCTGGAGATAAAGTAATTCCTCCAGAATATGCTATTCAAGAGGTTTTATGGTCACATGTTGTTGAAGTTATTCAATCTCGTAGTGGTCTTCCACTAGATTGTATTGTTTCTATACTTTCTAGCATCCTCAATTTTTTCTTGCGGTGTTACCCTTATAAACCCCAGTATGCAGATCGTGTTTTTCAATACATTAACGAACATATCATTAACCAGCCCTCCTTACGAAGCGCTCTTCATGAACGTCCATTACAAAAAAGTCTTTGCGCAATTTTATTGCTTCCTCTTACATATTTCCCTTCCTTTTCTTACTGTCTTGAGCTACAGAATTTCCTTCCTGTATTTAATGCGCAAGATCCAAACCTTCGTTACGATATTGCAAGAATGATTGTTCAAAAGATAATTGAAAAAGGCCACTCATTAAGTGAGCTTACAGAAGCACAAGAGCTACTAGGTTTTGTGTCTGTCATTATTGAAAAAAAGGGAGTTGATAGCCTTGATGACTTACAAAACGTTGCTTTGATGGTTCATTATTTGAATAATGATGACCCGCAAATTCAAATAGAGATACTTCGATCCTTGAAAGATACATTTATAAAAGCCGGTGAGAATGTCAAATATCTTTTGCCGGTCGTTGTTAATAGATGCATATTCCTTGCACGAAACTTTCGAATATTTAAGTGTATGGATTGGGCTGAGAAAGTTCGTCTTCTTTGGGAATTTGTAAACACTTGTATCAATGTTCTTTACAAAAACGGAGATTCTTTAGAGCTGTGCCTTGCATTATATTTAAGTGCCGCTGAAATGGCTGATCAAGAAAATTATCCTGATTTTGCTTACGAATTTTTCACACAGGCGTTTTCTATTTACGAGGAGTCTGTCCTTGACTCTGAATTGCAATATCAACAATTACTAATGATTATCGGGAAATTACAGAAAACTCGTAATTTCTCTGTTGATGATTATGACACGTTAATTACAAAGTGCACTTTATATGCTTCAAAGCTTTTGAAAAAGCCTGATCAATGTTGTGGGATTTACTTGGCTAGTCATCTATGGTGGCAGGTCGCATCAGGAGAAGATTCAAGGCCTTTTCAGGATCCAAAACGGGTACTCGAATGTCTGCAAAAAAGTCTCAAGATTGCTGATGCTTGTATGGATCAGCTTACTAGCCTGAAGCTTTTTATCAATATATTGGAACGTTACTTTTATTATTATGATCAGCATTGCGAATCTATCATTGCCAAACATATAAGTGGACTAATTGACCTTACAGAGCAGAACATGAGATCCATTTTAATATCCTCGCCTGCCGACCTTATTGCTAGTGATCCAAGAGCGTATGCTAGTTCGATCTGGGAGGTTGCTAATGTATCGGTAATTGATTCTCTTAAAAATCATTTAGAAAGAGCAACTGCTTACGCAGAGAAACGTTCTGAAGATGAACGTTGGTCTAGTATTTTTCAA***gctgcaggtggagctgcagctgcaggtgga*ATG**GATTACTTCTTTAAAAGTCCAATCGACGTTGATCTTCATTTGGACAATGAAGAAGAACGAACGTTCGTTGACTATGAGTTTGAACAGGGCCGAAAAGACAAGGCGCCAATTTACGAAAGTGATGAGACTGTGAAAGGAACAGTGATGATTCGTTTAAAAGATGGCCGCAAGTTGGACCACGATGGTGTGAAAATTGAGTTTATTGGACAGATCGAAAATACTTACGACAAAGGAAATATTCATGAATTTACTAGAAGCGTACAGGAGTTAGCTTCTCCTGGGGAAATGAGGCATGCCCAAATGTTTGAATTTGAGTTCAAGCATGTTGATAAACCTTATGAATCCTATATTGGCAAAAACGTTAAACTCCGCTATATATGTCGCGTTACGGTTTCCAGAAAGATGAAAGATGTCATTCGTGAAAAAGATCTGTGGGTCTATCGATTTGAGAATGAACCAGAGACAAACAGCTTAATCAGAATGGATGTGGGCATTGATGAATGCCTACACATAGAATTTGAATACAGCAAAAACAAATATCATTTAAAAGATGTTATTATTGGGAAAATCTACTTTATCCTTGTACGCATCAAGGTTCAGCGGATGGAAGTCAGTATCATACGGCGTGAAACGATCGGGACATCACCAAACCAGTACAGTAACAGTGAAACGATTACCAGATTTCAAATTATGGATGGTAATCCTAATCGTGGGGAAACAATTCCTTTACGGATGTTTTTGAATGGCTACGCTTTAACACCAACATTTCGAGACGTTAATAAAAAATTTAGTGTGAGATATTATTTGAGTCTAATTTTGGTGGATGAGGATCAAAGGAGATATTTTAAACAATCTGAAATAACCTTATGGAGAAGAAGGGATGAGCATGAG**TGA**CCCGGGatattata

The three proteins are separated by hinges of 10 alanine and glycine residues

**Protein (CSC)**

MVVLVIGDFHIPDRAPKLSEKFRQLLIPGKISQIICLGNLTSTSVYEYLKHVCSDLKLVKGAFDISSKAPIAGKITLGSFKIGYTNGHLVVPQDSPEALSILAREMDADILLFGGTHKFAAYELDGCFFVNPGSATGAPNVSAVEDDEKIVPSFVLMDVQGAVLILYVYRIFDGEVRVEKMQYRKPE**AAGGAAAAGG**MNGINTANEEITRSLEESLNICKQSSRLMQRNLQTGRLMDAFRNCSISLVEMRNSALTPKQYYELYMFNMESLRLLGGTLLETHLNGTHNLMDLYELVQYAGSIVPRLYLMITVGSAYLETPNALVREIMNDLLDMCRGVQHPLRGLFLRHYLLTQTRKGLPLGSEDEEDASRKGTVLDSVKFLVINFTEMNKLWVRIQHLGPIKEFSKRTQERNELKVLVGLNLVRLSQLNLDIDTYRDHVLPAIIEQIIECRDSLAQEYLVEVICQAFSDNMHLQTLDTYFGTVIKLSPSVNVTQLVVAMLNRLTDYVQREYESDSSNEDESETVTEKLGDIKINEEVQQKDEQECPGDKVIPPEYAIQEVLWSHVVEVIQSRSGLPLDCIVSILSSILNFFLRCYPYKPQYADRVFQYINEHIINQPSLRSALHERPLQKSLCAILLLPLTYFPSFSYCLELQNFLPVFNAQDPNLRYDIARMIVQKIIEKGHSLSELTEAQELLGFVSVIIEKKGVDSLDDLQNVALMVHYLNNDDPQIQIEILRSLKDTFIKAGENVKYLLPVVVNRCIFLARNFRIFKCMDWAEKVRLLWEFVNTCINVLYKNGDSLELCLALYLSAAEMADQENYPDFAYEFFTQAFSIYEESVLDSELQYQQLLMIIGKLQKTRNFSVDDYDTLITKCTLYASKLLKKPDQCCGIYLASHLWWQVASGEDSRPFQDPKRVLECLQKSLKIADACMDQLTSLKLFINILERYFYYYDQHCESIIAKHISGLIDLTEQNMRSILISSPADLIASDPRAYASSIWEVANVSVIDSLKNHLERATAYAEKRSEDERWSSIFQ**AAGGAAAAGG**MDYFFKSPIDVDLHLDNEEERTFVDYEFEQGRKDKAPIYESDETVKGTVMIRLKDGRKLDHDGVKIEFIGQIENTYDKGNIHEFTRSVQELASPGEMRHAQMFEFEFKHVDKPYESYIGKNVKLRYICRVTVSRKMKDVIREKDLWVYRFENEPETNSLIRMDVGIDECLHIEFEYSKNKYHLKDVIIGKIYFILVRIKVQRMEVSIIRRETIGTSPNQYSNSETITRFQIMDGNPNRGETIPLRMFLNGYALTPTFRDVNKKFSVRYYLSLILVDEDQRRYFKQSEITLWRRRDEHE-
