# Supplementary material for: Analysis of the SNARE Stx8 recycling reveals that the retromer-sorting motif has undergone evolutionary divergence
Source: PLoS Genet. 2021 Mar 31;17(3):e1009463. doi: 10.1371/journal.pgen.1009463 (PMC8041195; doi:10.1371/journal.pgen.1009463)
Supplement: S8 File — Relevant sequence information about the BiFluorescence Complementation (BiFC) analyses is provided. (DOCX) [file pgen.1009463.s016.docx]

**S8 File. Information related to the BiFC analysis**

- Construction of VN-Stx8

The Venus YFP N-terminal half was amplified as an *Apa*I*/Mlu*I DNA fragment using the following primers:

**VN-ApaF:** tatatataGGGCCCATGGTGAGCAAGGGCGAGGA

**VN-MluR:** tatatataACGCGTaccagtaccaccagaaccCTCGATGTTGTGGCGGATCTT

The underlined sequence corresponds to the linker

This fragment was used to replace the GFP in the pINTH+GFP-Stx8 plasmid

- Construction of Snx3-VC

- The following primers were used to tag Snx3 at its C-terminal end using the PCR-amplified module insertion method described by Bähler (1998)

**Snx3CTtag-F:**

TTGCTGGACATCCTCTTATTCAAACCCATTCTCGTGTTTTATCTTCTTTTTTACAAAGCCCGGAATTCAAGCCTACGCCACGGATCCCCGGGTTAATTAA

**Snx3CTtag-R:**

AAATGGTAATTAAACTTTGTAACAGATTGATTTCCAAATTTTTCTTTTAAAATAAAAAAAAAGATTGACAAAAATTAACAGAATTCGAGCTCGTTTAAAC

- Plasmid **pFA6A+VC155:KANMX6** (a generous gift from S. Labbé, Université de Sherbrooke. Canada) was used to amplify the DNA fragment used to transform *S. pombe*.
