# Supplementary material for: Analysis of the SNARE Stx8 recycling reveals that the retromer-sorting motif has undergone evolutionary divergence
Source: PLoS Genet. 2021 Mar 31;17(3):e1009463. doi: 10.1371/journal.pgen.1009463 (PMC8041195; doi:10.1371/journal.pgen.1009463)
Supplement: Attachment — Submitted filename: Point-by-point answer to Reviewers.docx [file pgen.1009463.s017.docx]

**Responses to the Reviewers’Questions**

**Reviewer #1:** In the study by Yanguas and Valdivieso a raft of data detailing the localization of the pombe Syntaxin 8 protein (Syt 8) is presented. The authors report that Syt 8 functions at the endosome and that the retromer complex mediates its trafficking between the endosome and the Golgi complex. They go on to show that a sorting motif in the Syt 8 protein is necessary for its localization and that the sorting motif is similar to one on the mammalian polycystin protein that has been reported to be a cargo of retromer.
I think the data, as presented, is largely solid and the conclusions are mostly well supported. I have two specific suggestions for experiments that, I feel, could strengthen this study:

**1.** The authors claim that Snx3-retromer is required for localizing Syt 8. This is not an unreasonable claim but I believe that the authors could enhance their study if they were to perform a native IP (as they have in figure 5 in the background of a snx3Δ (and possibly vps5Δ also). This would determine if it could be Snx3 making a direct contribution to cargo (i.e. syt 8) recognition as may be occurring. At present, it is not clear which of the retromer subunits is actually binding to Syt 8 (Vps35p? Vps26p? Vps5p? or perhaps Snx3p?) and investigating a little further which of the retromer proteins is responsible for binding to Syt 8 would be a worthwhile experiment.

In the new figure 4 we show that the amount of Vps35 that coimmunoprecipitates with Stx8 is reduced in the absence of Snx3 and in the absence of Vps5. We also show that Stx8 interacts with Snx3 by Bimolecular fluorescence complementation (BiFC). In our opinion, these results strengthen our hypothesis that Stx8 is recycled by the Snx3-retromer

**2.** Do the stx8 mutants that do not bind retromer (and therefore do not localize) possess any ability to rescue the salt-sensitivity phenotypes demonstrated by the stx8Δ cells in figure 1? By examining if the mutants that can't bind retromer are salt-sensitive, the importance of the correct trafficking of Syt 8 to its function can be established and this should be a relatively simple experiment to carry out.

We had performed this experiment before. We crossed the *stx8Δ* null mutant with strains expressing GFP-Stx8, GFP-Stx8(ΔSN), GFP-Stx8(132-141)A, and some of the point mutants (I133A, E134A and M135A). All the *stx8Δ* GFP-Stx8 clones analyzed were resistant, as the WT control. All the *stx8Δ* GFP-Stx8(ΔSN) clones were sensitive, as the *stx8Δ* control. Surprisingly, in the case of *stx8Δ* GFP-Stx8(132-141)A, *stx8Δ* GFP-Stx8(I133A), *stx8Δ* GFP-Stx8(E134A), and *stx8Δ* GFP-Stx8(M135)A we found differences in the sensitivity between clones.

The figure shows some examples.

This variability suggests that there is either a suppressor or an enhancer of the salt sensitivity when Stx8 recycling is compromised. Nevertheless, the sensitivity is never as drastic as that shown by cells bearing a deletion in the SNARE domain. These results suggest that once the SNARE has mediated vesicle fusion to the target membrane at the PVE, its retromer-mediated recycling is not essential. This recycling might contribute to full efficiency, but the cell would be able to cope with a reduced recycling. The strong potassium sensitivity exhibited by the *vps35Δ* mutant must be produced by the defective recycling of Stx8 and other proteins.

We had not included these results in the manuscript because we thought that they might add confusion. However, since it is a reasonable questions that the readers may come up with, we now include a supplementary figure showing drop tests with clones that fully or partially complement the sensitivity (new S5 Fig), and discuss this results in the new text.

**Reviewer #2:** The cellular trafficking of transmembrane proteins is orchestrated through a variety of sorting motifs that interact with evolutionarily conserved protein complexes. Failure to interact with the correct sorting complex will lead to the miss-trafficking of the integral proteins (cargoes). The manuscript by Yanguas and Valdivieso explores the molecular evolution of a sorting motif for the retromer complex, a coat complex that contributes to the retrograde trafficking of cargoes from the endocytic system to the trans-Golgi network (TGN). The authors dissect the retromer sorting motif of the SNARE protein Stx8 in S. pombe and reveal that such a motif has undergone evolutionary divergence. The SNARE protein Stx8, homologue of the mammalian Syntaxin 8, functions in vesicle fusion at the pre-vacuolar endosome (PVE), this being the equivalent of late endosomes/lysosomes in mammalian cells. It is shown that Stx8 cycles between the PVE and the TGN, the yeast equivalent of early endosomes, and that this retrograde recycling is mediated by retromer subunits Vps35, Vps5, Vps17 and Snx3. The authors use truncation mutants and site directed mutants to identify the essential residues (133IEMeaM138) that compose the peptide sequence for the interaction with the retromer complex. Intriguingly, the retromer-binding sequence of Stx8 is distinct to the canonical retromer sorting motif Φx[L/M/V], but it shares similarities with that of Polycistin-2 (PC2), this being a retromer cargo with a unique GLEIEMQRI sorting signal. Based on the alignment of the two motifs the authors establish a common Gx[-]IEMxx(I/M) signal, and a degenerated (loose) Ψ[+/-]ΨxxΨP motif that could also accommodate the canonical retromer signal. Finally, bioinformatic analysis reveals the presence of such motif in few fungal Stx8 and showed that the divergence of these motifs matches the phylogenetic divergence of Schizosaccharomyces species.

We have changed “degenerated” for “loose” in the new text.

Overall, the experiments appear technically sound, and the results are generally convincing (especially the identification and validation of the Stx8 sorting motif by use of truncation mutants and point mutants). In a few cases, additional controls may be needed to support specific conclusions, and some caveats may need to be stated. There are a number of issues with the paper that need to be clarified, most importantly the authors should avoid extending their conclusions beyond their own data, and avoid generalisations. For example, the identified Stx8 motif for retromer sorting has undergone evolutionary divergence, but this might not be the case for other retromer cargoes (Pkd2) or other retromer-binding motifs. The diversity of retromer sorting motifs (and their evolution) is an important aspect of the intracellular trafficking field that has long been underlooked. The findings in the present study are clearly of interest to understand the retrograde trafficking in yeast and, more broadly in eukaryotic cells, and thus provides important and relevant insight. We recommend publication if the caveats are addressed.

Major points:

**1.** The Ψ(E/D)ΨxxΨ motif is present in S. pombe Stx8 and H.sapiens PC2, but not in PC2 from D. melanogaster and C. elegans. The authors conclude that Ψ(E/D)ΨxxΨ has undergone evolutive divergence through metazoan and that the mammalian PC2 (almost-identical to that of Stx8) is a case of convergent evolution. How can the authors exclude that D. melanogaster and C. elegans have lost their strict Ψ(E/D)ΨxxΨ motif and are hence exceptions? The authors should comment on this.

According to Tilley et al. (2018), the sequence of PC2 N-terminal region from *H. sapiens* and the same region in *Drosophila melanogaster* and *Caenorhabditis elegans* is highly divergent. Keeping this into account, and following the Reviewer’s suggestions, we have used Scanprosite to detect possible [ILMV]-[EDHKR]-[ILMV]-X-X-[ILMV] loose motifs in several metazoan, including different *Drosophila* and *Caenorhabditis* species. We found that the GLEIEMERI motif is conserved in mammals, with the E at position 7 being the only variable residue. At this position, a Q instead of an E is present in 8 out of the 16 sequences analyzed.

The motif was more variable in fish; we analyzed 9 sequences (including *Danio rerio*) and the comparison of the motifs detected in the scan led to an XEG(I/L)EMEX(I/M) consensus motif, with an I at position 4 in eight sequences and an I at position 9 in seven sequences. The analysis of four *Caenorhabditis* species produced hits that led to a loose XXΦΨ[-]ΨxxΦ motif. The motifs in the most closely-related species (*C. brigsae* and *C. remanei*) are identical (WNLVDVALL), while those in *C. brenneri* (NENIEMIEL) and *C. elegans* (RSFMEVGGY) are more divergent. The scan produced hits in the five *Drosophila* sequences that we analyzed, and their comparison led to a loose XL[+]Ψ[+]L[+]X(L/M) motif. We also analyzed the *Schizosaccharomyces* species; the comparison of the Scanprosite hits led to a loose ΦXXI[+-]ΨXXΨ motif, with the sequences from the closely-related species *S. octosporus* and *S. cryophilus* being identical (MGIIKIGFM) and those from *S. japonicus* (FLAIKLPIV) and *S. pombe* (AYGIEIKKV ) more distant.

When we compared all the sequences at one time, the most represented residues led to the XXXΨ[+-]ΨXXΨ consensus, which might accommodate the ΦX(L/M/V) canonical sorting motif, although no aromatic (Ω) residues were present. We think that the more sequences we compare, the looser the motif is. It is when we analyze sequences from closer organisms when we can see a phylogenetic relationship between them.

The motifs in *D. melanogaster* (NLRLRLRTL) and *C. elegans* (RSFMEVGGY) are quite divergent from that in *H. sapiens*, and probably this is way they had not been detected by Tilley et al. (2018).

The results are included in the new figure 9 and discussed. They still indicate certain degree of evolutionary divergence into each group and between groups. Additionally, they support the notion that the presence of almost-identical motifs in two cargoes from distant organisms (*S. pombe* Stx8 and human PC2) is a case of evolutionary convergence.

**2.** The lack of a broader molecular phylogenetics analysis of retromer sorting motif(s) across several cargoes and other organisms makes it premature to draw conclusions regarding the evolutionary trend of the retromer motif(s). What the data of Yanguas and Valdivieso show is that the retromer motif is probably looser than what was thought before, and that some cargoes have more similar retromer sequences than others. We fully appreciate that the retromer-binding sequence of Stx8 has undergone evolutionary divergence, but a more systematic bioinformatic approach will be required to investigate whether this is an exception or whether this is the case for all other retromer-binding sequences. We suggest that the authors provide stronger evidence supporting the molecular evolution of the retromer motif or alternatively tone down their conclusions.

Although the results described above favor the idea that, at least the Ψ(E/D)ΨxxΨ sorting motif in PC2 and Stx8 have undergone evolutionary divergence, we agree with the Reviewer. Without extensive and more specialized phylogenetic analyses of this and other retromer sorting motifs in many other organisms, we cannot conclude that this is a general phenomenon. The results point to evolutionary divergence, but they are not conclusive. We have changed the abstract and author summary to soften our conclusion that the motif has undergone a general evolutionary divergence. We tell that our initial phylogenetic analyses suggest that this motif might have undergone evolutionary divergence. At the end of the discussion we also state that more specific analyses, and analyses that include other retromer sorting motifs, will be required to determine whether the evolutionary divergence of the retromer-binding sequence is a general rule.

**3.** Figure 1 general. Have the protein levels of Stx8 and Vps35 (in the Stx8Δ and Vps35Δ) been validated with western blotting? Does the reduced intensity of Vps10-GFP signal in Stx8Δ correlate to an increased turnover/degradation of such protein in vacuoles. The authors should use quantitative western blotting to compare the total levels of Vps10-GFP in wt and Δ mutant lines.

We think that the Reviewer was concerned about Stx8 level in *vps35Δ* and the level of Vps35 in *stx8Δ*. We have performed this analysis and found no differences in the level of each protein in the other mutant. We include this information in a supplemental figure (S1 Fig).

We now include a western blot showing that the level of Vps10-GFP in the WT and *stx8Δ* and *vps35Δ* strains is similar (new figure 1B).

**4.** Figure 1H. The results showed here are robust but the cleavage of free GFP could be an indirect effect (e.g. overexpression level). To make sure that the observation is indeed a vacuolar degradation phenotype, please rescue the phenotype with inhibitors of the vacuolar pathway (e.g. inhibitors of the vacuolar ATPase).

To show that the GFP accumulation is due to vacuolar cleavage, we have introduced the Ub:GFP-Cps1 construct in *stx8^+^*cells bearing deletions in the *isp6^+^* and/or *psp3^+^*genes, coding for the major *S. pombe* vacuolar proteases. Deletion of both proteases is required for full cleavage in the control. Similarly, the GFP band does not accumulate in the *stx8Δ* *isp6Δ* *psp3Δ* strain. These results are now included in the new S2 figure, and commented in the text.

**5.** Figure 2. To confirm the specificity of the phenotype the authors should rescue the localisation of Vps10-GFP in the stx8Δ cells by rescuing the phenotype with re-expression of Stx8 in the Δ cells.

In the new figure 2C, we show that reintroducing Stx8 but not Stx8(ΔSN) to a *stx8Δ* mutant suppresses potassium sensitivity. In the new figure 2E we show that a plasmid bearing Cherry-Stx8 suppresses the defect in Vps10-GFP distribution. Additionally, this strain allowed the observation of Vps10 and Stx8 colocalization.

**6.** Figure 3. Authors should confirm that the altered steady-state distribution of GFP-Stx8 in *vps35Δ* cells, also correspond to a decreased co-localisation with a TGN marker.

We have analyzed the colocalization between GFP-Stx8 and Cfr1-RFP in *vps35Δ* and have found that it is significantly reduced with respect to their colocalization in the WT. We have included this result in the new figure 3D

**7.** Figure 5D and G. The biochemical experiments exploring the interaction between retromer and Stx8 should be solidified. We believe that it is important to repeat and quantify the degree of this interaction over at least n = 3 independent experiments, or alternatively strengthen the results by showing direct binding between Stx8 132-141 and the retromer complex.

We have repeated the coimmunoprecipitation between different Stx8 variants and Vps35. The two additional experiments are shown in the supplemental figure S6. The degree of interaction between exomer and the Stx8(122-131)A and Stx8(142-151)A forms varied between experiments. On the other hand interaction between Vps35 and Stx8(132-141)A was always lower than that Vps35-Stx8 interaction. The statistical significance is shown in the new Fig 5F.

Nevertheless, to strengthen those results we have performed two-hybrid analyses using a Stx8 variant that lacks the transmembrane helix (we have termed it Stx8*). Initially, we could not detect direct interaction (growth on YNB-His plates) when the *Saccharomyces cerevisiae* tester strain (AH109) bore plasmids with Stx8* and Vps26, or with Stx8* and Snx3. However, the fact that interaction between Snx3 and Vps26 could not be detected although this interaction has been reported using other methods (Lucas et al., 2016), pointed either to a technical problem or to a weak interaction. Trying to overcome the caveat, we purchased a fusion *vps29^+^*-*vps35^+^*-*vps26^+^* custom gene, consisting on the three retromer cargo-selective complex (CSC) genes separated by hinges of 10 A+G residues. We observed growth on YNB-His plates when AH109 was cotransformed with Stx8* and CSC. It is still possible that additional proteins (maybe Snx3) stabilize the binding of the retromer CSC to cargoes because growth on YNB-His plates was slower than on YNB-Leu-Trp. To get more solid results we used an alternative technique, Bimolecular fluorescence complementation (BiFC), which allowed us observing Stx8-Snx3 interaction. Thus, these additional experiments confirmed that Stx8 is a Snx3-retromer cargo.

The fact that the Stx8* variants Stx8(132-142)A* and Stx8(E134)A* failed to promote growth on YNB-His plates confirmed that these residues are involved in the direct Stx8-CSC interaction (new Figure 5G).

**8.** Figure 8A. Please note that the sequence of PC2 is G**L**EIEMQRI instead of GIEIEMQRI. Also, the consensus between Stx8 and PC2 should be Gx[-]IEMxx(I/M) (both D and E are negatively charged). Please be consistent with the use of symbols +/- in the consensus sequences throughout the manuscript and figures.

We thank the Reviewer for pointing this out. We have corrected the mistake in the PC2 sorting motif (figure 8). Regarding the [+-] symbols, we have changed to [-] in the text and figures when there is an E or D at that position and we have kept [+-] when the position is occupied by E, D, K, H or R.

Minor points:

**1.** It has been reported by Bean et al., 2017 that the retromer cargoes Ear1, Ymd8 and Ymr010w share a Ψ-E-(F/L) that could adhere to the first part of the Ψ[-]ΨxxΨ motif. Have the authors considered whether all these retromer binding sequences are just variation of a very loose and general retromer binding motif?.

Yes, as we mention in the discussion, “the IEM block slightly resembles the ΩEΩ motif of Kex2, Ear1, Ymd8 and Any1 (Bean, 2017), although the hydrophobic residues in these proteins (Y/F at the first position and F at the third position in the case of Ste13, Kex2 and Ear1) are larger because they bear aromatic chains. The IEM block also resembles the INL (ΨNΨ) motif in Ear1, where I and L are essential for recycling. Nevertheless, this motif is part of the bipartite ^453^PPGFEF^458^–^473^INL^475^ signal, while the IEMeaM motif is the Stx8 only sorting signal”.

We also discussed that …”Different cargoes interact with different sites at the retromer (Suzuki, 2019), an eventuality that helps to explain the diversity in sorting motifs. The determinant for the binding of these residues to the cargo might be a general aspect of their nature, which would permit certain variability”, and that another possibility would be that, “the binding determinant is a structural feature, which might be achieved by different residue combinations in the cargo motif”…. In other words, all the variations of a loose binding motif that allow their interaction with the key residues in the retromer subunits will be functional. We think that we are giving the same message with different words. Nevertheless, we have rewritten this paragraph, which now states that “Different cargoes interact with different sites at the retromer (Suzuki, 2019), an eventuality that helps to explain the diversity in sorting motifs, which seem to be variations of a very loose and general binding motif.”.

**2.** Have the authors considered testing some of the Vps26 mutants from Suzuki et al. 2019 to explore whether the retromer binding-sequence that they have identified in Stx8 binds the same surface of Vps26 that is involved in the interaction with Vps10 and/or Ear1?

Yes, we had considered this possibility but we have finally decided that this would be an extensive analysis, and that it might be the subject of an independent study in the future.

**3.** The authors state that “it is possible that the IEMeaM motif and the TM domain have to be separated by a minimum number of amino acids”. This would be consistent with the recent structure by Kovtun et al., 2018 showing the core retromer subunits Vps26/35/29 are not directly in contact with the lipid membrane but layered on the top of Vps5 oligomers. Can the authors comment on the possibility that the linker between the TM domain and the retromer sorting motif is required so that the residues composing the sorting motif can reach the distal retromer subunits?.

We thank the Reviewer for this suggestion. We have included this comment on the discussion.

-------------------
 Bean BD, Davey M, Conibear E. Cargo selectivity of yeast sorting nexins. Traffic. 2017;18(2):110-122. doi:10.1111/tra.12459.

Kovtun O, Leneva N, Bykov YS, et al. Structure of the membrane-assembled retromer coat determined by cryo-electron tomography. Nature. 2018;561(7724):561-564. doi:10.1038/s41586-018-0526-z.
 Suzuki SW, Chuang YS, Li M, Seaman MNJ, Emr SD. A bipartite sorting signal ensures specificity of retromer complex in membrane protein recycling. J Cell Biol. 2019;218(9):2876-2886. doi:10.1083/jcb.201901019
 Tilley FC, Gallon M, Luo C, Danson CM, Zhou J and Cullen PJ. Retromer associates with the cytoplasmic amino-terminus of polycystin-2. J Cell Sci. 2018; 131(11): jcs211342. doi: 10.1242/jcs.211342.
